# Quality of life, mental health, and socio-demographic differences across sex work settings: implications for specialized healthcare and support services

**DOI:** 10.3389/fpubh.2025.1703735

**Published:** 2025-12-04

**Authors:** Olivia Kalinowski, Franziska Kroehn-Liedtke, Gizem Kaya, Anastasiia Lotysh, Hristiana Mihaylova, Krisztina Sipos, Lena Karoline Zerbe, Meryam Schouler-Ocak, Wulf Rössler

**Affiliations:** Department of Psychiatry and Neurosciences, Psychiatric University Clinic of Charité at St. Hedwig Hospital, Berlin, Germany

**Keywords:** sex work, prostitution, trafficking, mental health, setting, healthcare service, quality of life, support

## Abstract

**Background:**

Existing research shows that quality of life (QoL), mental health, and working conditions vary widely across sex work settings. Street-based work, for example, involves different risks than, for example, online sex work. Little is known about what drives individuals into specific environments. This study examines whether work setting predicts working conditions, QoL, mental health, and reported socio-medical needs, and explores factors predicting the work setting. The aim is to inform recommendations for more tailored healthcare and support services.

**Methods:**

Data come from *PSYCHSEX*, a cross-sectional study conducted at the Department of Psychiatry and Neurosciences, Charité University Hospital Berlin (August 2021–August 2024), funded by the German Research Association (DFG). Structured quantitative interviews were conducted with 403 sex workers recruited through quota sampling to represent diverse settings, including private apartments, studios, brothels, massage parlors, clubs, clients' homes, vehicles, guesthouses/hotels, outdoor locations, and online platforms.

**Results:**

Work setting was significantly associated with QoL, mental health, and working conditions. Specific settings (e.g., studios, escort work, and online platforms) were linked to better QoL, mental health, and working conditions than others. Socio-demographic factors like migration status and homelessness, along with distinct self-reported reasons for entering sex work, such as personal preference, lack of alternatives, or funding one's education, predicted work setting. Reported socio-medical needs differed across settings.

**Discussion:**

Sex work is shaped by diverse settings, conditions, and individual pathways. Variation in QoL, mental health, working conditions, and unmet support needs underscores the need for setting-specific healthcare and social services. Structural factors such as housing instability and migration status play a key role and should be addressed through targeted policies and interventions. These findings highlight the importance of context-sensitive, evidence-based support to improve the wellbeing of sex workers across different environments.

## 1 Introduction

While definitions for sex work may vary across cultural and legal contexts, the term generally refers to participation in activities where sexual services are provided in return for economic compensation. Despite its widespread nature, sex work remains under-researched, with significant gaps in our understanding of it, including the accurate number of individuals involved. In Germany, estimates of the sex worker population range from approximately 90,000 to significantly higher numbers (1, 2), highlighting the challenge of capturing the full scope of this industry. This is due to varying definitions of sex work (e.g., whether working online is also considered sex work) and also due to the oftentimes missing distinction between working as a sex worker every day vs. working occasionally or periodically. Further methodological issues, such as high mobility among sex workers between districts, cities, and nations, complicate correct estimates, besides the fact that sex workers are generally harder to reach than other groups. About 90% of sex workers are said to be female (3).

Previous research has been conducted globally, reflecting diverse socio-cultural contexts (4, 5). This study centers on female sex workers in Germany, where sex work is legalized on several levels. It is important to underscore that sex work does not inherently equate to sex trafficking or coercion, and it should be noted that the concept of “agency” in this context remains a subject of academic debate. “Coercion” can span from economic necessity or a lack of other opportunities (e.g., due to work permits) to the involvement of a third party. This should be kept in mind when we, for example, write about “choice” throughout this article.

### 1.1 Sex work settings

For the purpose of quantitative research, sex work settings are often dichotomized into indoor and outdoor categories. However, this distinction fails to adequately reflect the complexity of sex work environments, which bear varying working conditions (4, 5). The oversimplification inherent in this binary framework may obscure crucial differences and thus limit the validity of findings. Given the limited empirical documentation of many sex work settings, a detailed account is not feasible (6). The subsequent overview aims instead to delineate principal differences across settings and establish a conceptual foundation for the readers.

In some settings (e.g., brothels), sex workers typically rent the facilities (5, 6). Financial gain, therefore, depends on the rent price, requiring workers to accommodate usually more than one client. In contrast, apartment- or hotel-based arrangements, where clients provide the venue, reduce the worker's financial risk. Specialized environments, such as BDSM studios and tantra or erotic bodywork practices, provide distinct forms of sex work (6, 7). Dominatrices engage in practices centered on power dynamics, often with minimal or no physical contact, whereas tantra and bodywork typically involve direct tactile interaction (7, 8). Sexual assistance represents a separate domain, offering sexual contact to individuals with disabilities (9). The classification of apartment-based sex work remains complex, as safety and working conditions may vary depending on whether the location is the worker's own residence, the client's home, or a rented space (5). Similarly, escort work blurs categorical boundaries: activities may range from companionship at social events to sexual encounters that sometimes simulate emotional intimacy (often referred to as the “girlfriend experience”), either self-organized or managed by agencies (6).

Pornographic performance constitutes another domain with heterogeneous working conditions (5). Performers may work independently or within structured productions, and the rise of digital platforms such as OnlyFans has enabled direct engagement with clients, monetized communication, and varying levels of anonymity through pseudonyms or partial concealment (6). Finally, outdoor sex work, including street-based encounters or those occurring in cars, parks, or public restrooms, typically involves brief, non-prearranged interactions. However, the boundary between indoor and outdoor work remains fluid, as some outdoor workers accompany clients to hotels or private residences (4, 5).

This variability underscores the need for a nuanced exploration of how different sex work environments impact the overall quality of life (QoL), mental health, and working conditions. Furthermore, it is necessary to investigate the factors that might drive individuals toward riskier settings to formulate supportive policies and interventions tailored to the needs and rights of sex workers while keeping in mind that there are various motivations for working as a sex worker.

### 1.2 Quality of life (QoL) and working conditions among sex workers

Qualitative findings show that the QoL of sex workers is a complex construct, influenced by a range of factors including income, work environment, health, access to health services, exposure to different types of violence, housing, personal relationships, competition, religious beliefs, reading level, and internalized or anticipated sex work stigma (10–14).

Certain subgroups of sex workers, such as those living with human immunodeficiency virus (HIV) (14), who inject drugs (15, 16), and victims of sex trafficking (17), have been found to experience a lower QoL. Some studies show that the overall QoL of sex workers is worse than that of the general public (18–20), while other studies, focusing on, for example, adult film performers (21), showed that the sex workers' QoL was even better than that of a socio-economically matched comparison group. This indicates that QoL correlates not only with individual characteristics but also with the type of sex work, which is plausible, as working conditions, such as experiences of physical violence (4, 22) and salary, are known to vary immensely between (but also within) the specific fields (23–25). Specific working conditions also correlate with socio-demographic characteristics. Income, for instance, was shown to be associated with the sex workers' age in one study. It was found that a sex worker's hourly earnings are reduced by 4.5% for each year of age (26). There are no established frameworks concerning working conditions in sex work settings, but the literature indicates that the conditions are determined by factors such as autonomy, safety, exposure to different types of violence, police interaction, stigma, discrimination, and factors such as the availability and quality of sexual health services, mental health care, and community support (10–14). Working conditions might also entail the sexual services offered (27, 28).

Street-based sex work is closely associated with numerous adverse working conditions, including a significantly higher risk of human immunodeficiency virus (HIV) and other sexually transmitted diseases (STDs) compared to other forms of sex work. Additionally, street-based sex workers often lack family support and face the highest levels of physical and sexual violence, abuse, and stigma among sex workers, contributing to a heightened risk of premature mortality (29, 30). The QoL was found to be worse among street-based sex workers compared to other sex workers (16).

### 1.3 Mental health of sex workers

Similar to the QoL, it has been shown that the mental health of sex workers varies significantly across occupational settings. Sex workers experience mental disorders more frequently than other professionals, such as social workers (31); however, many studies display methodological flaws or, for example, focus on specific subgroups of sex workers, such as street-based sex workers, and generalize their findings (5). Mental health issues like depression, anxiety, and post-traumatic stress disorder (PTSD) are notably prevalent among street-based sex workers, and a high incidence of intravenous drug use is reported (4, 32, 33). Studies that compare the mental health of sex workers across diverse work settings are scarce (22, 34–36). Some studies also focus on the mental health of survivors of sex trafficking (37). In accordance with the World Health Organization (38), mental health is understood as “a state of mental wellbeing that enables people to cope with the stresses of life, realize their abilities, learn and work well, and contribute to their community” in this study. We use the term mental disorders to refer to clinically relevant disturbances in cognition, emotion, or behavior, consistent with diagnostic frameworks such as the ICD-10 and DSM-5. While it is plausible that sex work or a specific sex work Setting can contribute to the development of mental illnesses, it is also possible that individuals with mental illnesses enter the sex work industry, as it is well-established through the social drift hypothesis that people with mental illnesses struggle within the regular job market, leading to economic constraints (39). The following example should illustrate this. A close connection between substance-use disorder, homelessness, and working on the streets has been found (5). While it is not possible to determine causality due to study design, it seems plausible that individuals with substance-use disorder might engage in sex work to finance their substance use. However, it also seems plausible that individuals develop substance-use disorders due to the exploitative conditions of street-based sex work (4, 5). There are no studies examining the causal relationship between sex work and mental illnesses, as establishing causality would require methods that are largely infeasible in this population for the reasons outlined at the beginning of the introduction.

### 1.4 Reasons for starting sex work and pathways into specific work settings

It was shown that the motivations for entering the sex work industry are multifaceted. Consistent across the literature is the prominence of financial incentives. These include reasons such as being incapable of other work (40, 41), making more money than with other work (40–42), or using sex work as supplemental income (40, 43). Other studies found that providing for a child was another finance-related reason for entering sex work (41, 42, 44–46). Poverty due to widowhood or separation (43, 47), family debt (47), lack of family support, as well as lack of education, are also known economic reasons for being a sex worker (48). Social reasons for being a sex worker include a lack of legal or social protection (and therefore vulnerability to exploitative third parties in the sex work industry), negative social circumstances in life, such as eviction and homelessness (49), vulnerabilities due to migration (45, 50), or religious–cultural traditions, such as in the case of “Devadasis,” a historical religious practice in India that became increasingly entangled with sexual exploitation, a shift inflected by colonial power dynamics (8, 51). Drug use is also mentioned as one of the reasons for entering sex work (45–47, 52). On the other hand, being a sex worker out of personal choice (50), for pleasure (46), because of the freedom and autonomy over their bodies, and because of flexible working hours is described in the literature (42, 43). These studies refer to self-reported reasons. There is also literature that indicates an association between childhood sexual abuse and entering sex work (53–55), suggesting unconscious pathways that might, similar to other types of trauma, create cycles of repetition and therefore lead to specific types of sex work. However, this hypothesis is not sufficiently explored yet.

In terms of entry reasons into a specific sex work branch, only a few settings were investigated. It was found that for the adult film industry and online sex work, financial considerations were the main reasons to start sex work in this field (56, 57). However, the narrative expands with the emergence of less conventional motivators. Social networking has surfaced as a considerable factor in entering the adult film industry. Curiosity or chance encounters with the industry also play a noteworthy role. A smaller fraction identified artistic expression, freedom, and even revenge as their motivators (56).

### 1.5 Research questions and study aims

In summary, the literature suggests that QoL, the prevalence of mental diseases, and working conditions vary across work settings. This means that some workplaces bear more or different work-related risks than others, raising the question of which factors lead individuals into specific, and especially riskier, work settings. The existing literature depicts multiple reasons for starting sex work and entering specific workplaces, but there is no extensive comparative overview across multiple work settings. The research landscape appears patchy, with several large research gaps. By acknowledging the vast spectrum of sex work, from street-based to online platforms, and recognizing the diverse motivations behind entering this industry—ranging from economic necessity to personal preference—our study aims to fill several crucial gaps by investigating the following research questions:

1. How is sex work setting associated with quality of life, mental health, and working conditions among sex workers?
  a. Is sex work setting a predictor of quality of life?
  b. Is sex work setting a predictor of mental diseases?
  c. Is sex work setting a predictor of working conditions?
2. Which factors are associated with engagement in specific sex work settings?
  a. Do socio-demographic factors predict specific work settings?
  b. Do mental diseases predict specific work settings?
  c. Do self-reported reasons for sex work predict specific work settings?
3. How are reported healthcare and support needs associated with sex work setting?
  a. Does work setting predict the reported needs?

## 2 Methods

### 2.1 Study design and ethical considerations

This study draws data from PSYCHSEX, a comprehensive cross-sectional investigation conducted from August 2021 to August 2024 at the Department of Psychiatry and Neurosciences at Charité University Hospital Berlin, with funding from the German Research Association (DFG: GZ: SCHO 772/4–1 and GZ: RO 948/7–1). The research employed structured quantitative interviews with a sample of 403 sex workers. A prior statistical sample size estimation was conducted for the parent study. To address the challenge of linguistic diversity, the questionnaires were translated into an array of languages, including German, English, Polish, Russian, Ukrainian, Turkish, Romanian, Bulgarian, and Hungarian. To guarantee the precision of these translations, they underwent a reverse translation process conducted by independent native speakers. These individuals were not only fluent in the languages concerned but also served as professional healthcare providers, ensuring a high level of expertise and understanding in the translation task. Interviews were performed both in person in Berlin (in a research office based on the hospital campus, in NGOs, or directly in the work settings, such as brothels and studios) and via video call. Ethical clearance was secured from the Charité University Medicine Ethics Committee, with stringent measures implemented to protect participant confidentiality and privacy. Data were collected anonymously, linking consent forms to anonymized IDs, thereby enabling participants to withdraw their data at any stage without compromising personal information. Informed consent was obtained from all participants. Furthermore, to support participants experiencing stress or in need of counseling, a psychiatric consultation session was established. Each participant was compensated with a remuneration of 50 euros for their involvement. The study adhered to the Declaration of Helsinki (2004) principles and the guidelines for good scientific practice (58).

### 2.2 Inclusion and exclusion criteria

The study focused on women aged 18–70 who were engaged in sexual activities (voluntarily or under coercion) for financial or material compensation. Recruitment was facilitated through outreach programs, online platforms, and collaborations with pertinent organizations. Only women who stated they had (or have had) clients in Berlin were included. The imposition of geographical constraints originated from the clients' perspective, due to the high levels of both national and international mobility observed among sex workers in Germany. “Woman” encompasses individuals who identify as female, those assigned female at birth, or those perceived as female by their clientele. This gender-specific approach was chosen to diminish population heterogeneity. Eligible participants were required to demonstrate proficiency in at least one of the interview languages and to be active in one or more specified areas of sex work. Participants who visibly appeared to be under the severe influence of alcohol or other drugs, and participants who showed signs of psychotic symptoms such as hallucinations, were excluded from this analysis.

### 2.3 Data collection

We included settings such as personal apartments, studios, brothels, massage parlors, clubs, clients' apartments, vehicles, guesthouses/hotels, outdoor spaces, and online platforms. To ensure an equal representation of different sex work settings, quota sampling was used to recruit participants. They were directly invited to participate in various locations, as well as through online platforms and social media. Flyers were distributed at these venues to promote the study. Recruitment efforts were further supported by collaboration with NGOs. Interviews were conducted by trained clinical psychologists and graduate research assistants studying and training in medicine, psychology, public health, and social work.

### 2.4 Measures

The Short-Form-12 (SF-12) Health Survey was employed to assess health-related quality of life. The SF-12 is an abbreviated version of the SF-36 and includes twelve questions covering eight (physical and mental) health domains (59). The Mini International Neuropsychiatric Interview (MINI) DIPS Open Access (60) was used to assess the psychiatric diagnoses according to ICD-10 and DSM-5 criteria. A description of other variables can be found in Supplementary file S1.

### 2.5 Data analysis and statistical considerations

To stabilize the statistical models, several adjustments were made. First, a correlation matrix of work settings was created. Highly correlated settings were merged into new variables if participants reported working in at least one. Six initial settings were recoded into three categories: “Street and/or car,” “Hotel and/or client's apartment,” and “Escort and/or diverse places (girlfriend experience).” Due to small sample sizes, “Massage Parlor” (*n* = 15) and “Caravan/Camper” (*n* = 4) were excluded. Diagnoses assessed via the MINI were grouped into broader categories: anxiety disorders, affective disorders, obsessive-compulsive disorders, PTSD, eating disorders, somatization, sleeping disorders, and addiction. All analyses were conducted using SPSS (version 29) (61) and R (version 4.5.1) (62) using the lme4 and lmerTest packages (63, 64). Statistical significance was set at *p* < 0.05. A total of three interviews (*n* = 3) were excluded from the analysis due to indications of deliberate response falsification. We used a range of regression models to examine associations between workplace type and various outcomes, including classical linear models for continuous outcomes, logistic regression for binary outcomes, and ordinal or partially parallel ordinal regression models for ordered categorical outcomes. A full description of the statistical protocol is provided in Supplementary file S2. Since participants could report working at multiple settings, mixed-effects models were considered but ultimately not used. Because we have only a single measurement of mental health and other outcomes, there is no within-person variance to exploit. Consequently, attempting to “control” for individual characteristics across multiple settings would not isolate the effect of the workplace but would instead obscure meaningful between-person differences. For this reason, we opted for simpler models, acknowledging that associations reflect both workplace-related factors and pre-existing differences between participants. Overall, this analytical strategy acknowledges that participants may work across multiple settings but ensures that associations reflect meaningful between-person differences. It appropriately addresses the study's research questions within the limitations of cross-sectional data. Additional descriptive statistics were conducted to evaluate all the named variables across work settings. For a detailed description of the statistical protocol, including model diagnostics and assessment of assumptions, please refer to Supplementary file S2. The significance threshold for each predictor was adjusted using the Bonferroni correction per outcome to account for the increased risk of Type I error due to multiple testing. For details on significance threshold adjustments and the application of Bonferroni corrections, please refer to Supplementary file S2.

## 3 Results

**Figure 1:**
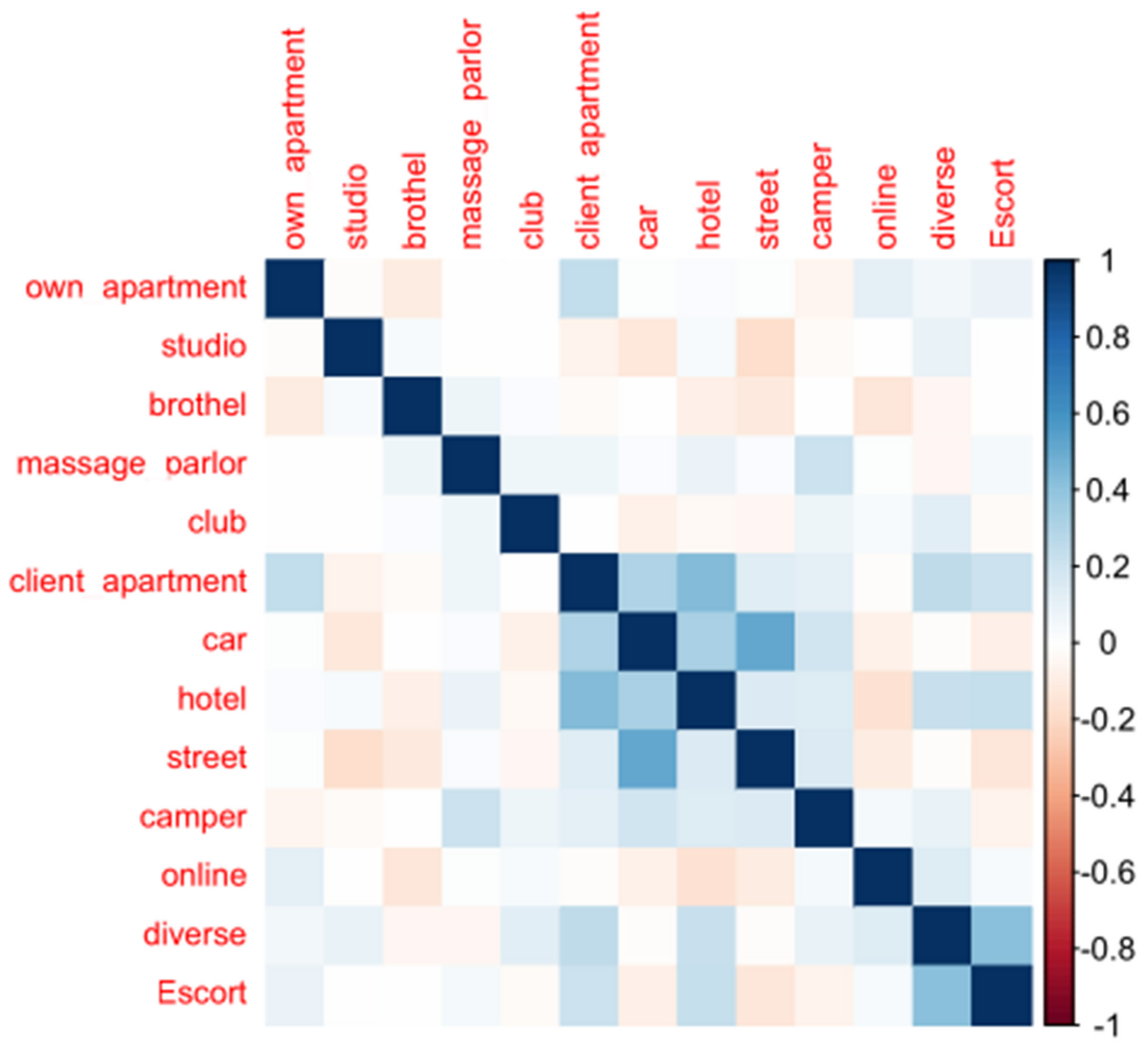
Correlation patterns across sex work settings.

The respondents reported a wide range of work settings, with hotels being the most common setting (18.72%), followed by online work (16.78%) and studios (14.11%) in the weighted analysis. Other frequently mentioned locations included the street (9.73%) and the client's apartment (8.71%), while brothels accounted for 7.03% of reported work settings. Less commonly reported locations were escort services (4.65%), own apartments and (strip) clubs (each 3.98%), cars (5.05%), and diverse or mixed locations (5.29%). Rarely mentioned were massage parlors (1.51%) and campers (0.46%). Other workplaces included atelier, train station, film studio, garage, cabin, cinema, public restroom, nursing home, sex shop, telephone, forest, and tent. The correlation matrix (see Figure 1) indicates that the correlation between workplaces was very diverse, ranging from low correlations to moderate and higher values, namely between hotel and client's apartment, escort and diverse places, as well as street- and car-based sex work. Negative correlations were observed, for example, between online work and working in a brothel setting. The socio-demographic characteristics of the overall sample can be found in Table 1. Descriptive analysis showed that the majority of participants held German citizenship (56.5%). The second most common citizenship was Bulgarian (7.09%) and Hungarian (5.44%).

**Table 1:** Sociodemographic and sex work–related characteristics of the study sample.

**Variable**	**Street/Car (*n* = 115)**	**Escort/Diverse (*n* = 103)**	**Client's apartment/Hotel (*n* = 227)**	**Online (*n* = 121)**	**Club (*n* = 34)**	**Brothel (*n* = 47)**	**Studio (*n* = 117)**	**Own apartment (*n* = 47)**
Mean age (SD)	35.17 (9.77)	30.14 (7.02)	33.92 (9.31)	30.21 (6.8)	32.5 (7.80)	37.17 (7.5)	33.70 (8.21)	31.28 (9.28)
Mean age at beginning (SD)	23.87 (8.56)	23.88 (5.67)	24.38 (7.85)	24.92 (6.86)	24.76 (7.05)	25.34 (6.39)	25.65 (6.90)	23.83 (7.3)
**Gender**								
Female	94.78 (*n* = 109)	90.29 (*n* = 93)	90.31 (*n* = 205)	91.74 (*n* = 111)	85.29 (*n* = 29)	97.87 (*n* = 46)	96.58 (*n* = 113)	87.23 (*n* = 41)
Male	0.87 (*n* = 1)	0	0.88 (*n* = 2)	1.65 (*n* = 2)	0	0	0.85 (*n* = 1)	0
Diverse	4.35 (*n* = 5)	9.71 (*n* = 9)	8.37 (*n* = 19)	6.61 (*n* = 8)	14.71 (*n* = 5)	2.13 (*n* = 1)	2.56 (*n* = 3)	10.64 (*n* = 5)
**Nationality and migration**								
German citizenship	38.26 (*n* = 44)	71.84 (*n* = 74)	58.59 (*n* = 133)	80.17 (*n* = 97)	61.76 (*n* = 21)	0.47 (*n* = 22)	65.81 (*n* = 77)	68.09 (*n* = 32)
Migration background	81.74 (*n* = 94)	53.40 (*n* = 55)	63.44 (*n* = 144)	46.28 (*n* = 56)	67.65 (*n* = 23)	0.53 (*n* = 25)	50.43 (*n* = 59)	59.57 (*n* = 28)
**Social network**								
Children	43.48 (n = 50)	19.42 (*n* = 20)	27.31 (*n* = 62)	23.97 (*n* = 29)	17.65 (*n* = 6)	0.83 (*n* = 39)	29.06 (*n* = 34)	21.28 (*n* = 10)
Mean number of children (SD)	2.24 (2.24)	1.85 (1.14)	2.14 (1.05)	1.97 (1.02)	2.17 (1.17)	1.73 (0.88)	1.79 (0.8)	1.8 (1.03)
Relationship	32.17 (*n* = 37)	56.31 (*n* = 58)	43.61 (*n* = 99)	61.16 (*n* = 74)	52.94 (*n* = 18)	46.81 (*n* = 22)	58.97 (*n* = 69)	51.06 (*n* = 24)
Does partner know about sex work?	91.89 (*n* = 34)	92.98 (*n* = 53)	88.78 (*n* = 87)	97.26 (*n* = 71)	100 (*n* = 18)	86.36 (*n* = 19)	91.18 (*n* = 62)	95.83 (*n* = 23)
Homelessness	51.30 (*n* = 59)	13.59 (*n* = 14)	34.36 (*n* = 78)	9.92 (*n* = 12)	20.59 (*n* = 7)	21.28 (*n* = 10)	14.53 (*n* = 17)	12.77 (*n* = 6)
**Education**								
Analphabetic	6.96 (*n* = 8)	0	2.64 (*n* = 6)	0	0	0	0.85 (*n* = 1)	2.13 (*n* = 1)
No qualification	28.70 (*n* = 33)	2.91 (*n* = 3)	14.98 (*n* = 34)	0.83 (*n* = 1)	5.88 (*n* = 2)	10.64 (*n* = 5)	0.1 (*n* = 12)	6.38 (*n* = 3)
Still a student	0	0	0	0	0	0	0	0
Elementary school	17.39 (*n* = 20)	4.85 (*n* = 5)	5.73 (*n* = 13)	5.79 (*n* = 7)	0	6.39 (*n* = 3)	0.85 (*n* = 1)	12.77 (*n* = 6)
Secondary school leaving certificate	22.61 (*n* = 26)	15.53 (*n* = 16)	22.03 (*n* = 50)	25.62 (*n* = 31)	17.65 (*n* = 6)	21.28 (*n* = 10)	16.24 (*n* = 19)	19.15 (*n* = 9)
Completed apprenticeship	8.70 (*n* = 10)	12.62 (*n* = 13)	7.93 (*n* = 18)	16.53 (*n* = 20)	2.94 (*n* = 1)	6.38 (*n* = 3)	11.11 (*n* = 13)	6.38 (*n* = 3)
Fachabitur	4.35 (*n* = 5)	4.85 (*n* = 5)	4.85 (*n* = 11)	7.44 (*n* = 9)	5.88 (*n* = 2)	10.64 (*n* = 5)	8.55 (*n* = 10)	6.38 (*n* = 3)
Abitur	13.91 (*n* = 16)	31.07 (*n* = 32)	20.70 (*n* = 47)	21.49 (*n* = 26)	41.18 (*n* = 14)	31.91 (*n* = 15)	17.09 (*n* = 20)	14.89 (*n* = 7)
University degree	4.35 (*n* = 5)	25.24 (*n* = 26)	21.59 (*n* = 49)	22.31 (*n* = 27)	20.59 (*n* = 7)	10.64 (*n* = 5)	33.33 (*n* = 39)	31.91 (*n* = 15)
Other degree	0	2.91 (*n* = 3)	1.76 (*n* = 4)	0	5.88 (*n* = 2)	2.13 (*n* = 1)	2.56 (*n* = 3)	0
**Sex work**								
Is sex work main source of income?	78.26 (*n* = 90)	68.93 (*n* = 71)	68.72 (*n* = 137)	69.42 (*n* = 84)	61.76 (*n* = 21)	65.96 (*n* = 31)	63.25 (*n* = 74)	68.09 (*n* = 32)
Are there other sex workers in the work location?	73.04 (*n* = 84)	59.22 (*n* = 61)	60.35 (*n* = 137)	78.51 (*n* = 95)	85.29 (*n* = 29)	91.49 (*n* = 43)	76.07 (*n* = 89)	42.55 (*n* = 20)
Involvement of third persons?	13.91 (*n* = 16)	18.45 (*n* = 19)	18.50 (*n* = 42)	19.83 (*n* = 24)	23.52 (*n* = 8)	21.28 (*n* = 10)	25.64 (*n* = 30)	21.28 (*n* = 10)
Social contacts beyond sex work?	59.13 (*n* = 68)	93.20 (*n* = 96)	79.74 (*n* = 181)	94.21 (*n* = 114)	88.23 (*n* = 30)	80.85 (*n* = 38)	90.6 (*n* = 106)	89.36 (*n* = 42)
Do they know about sex work?	80.30 (*n* = 53)	82.61 (*n* = 76)	79.65 (*n* = 137)	77.69 (*n* = 94)	89.66 (*n* = 26)	52.5 (*n* = 21)	82.29 (*n* = 79)	87.8 (*n* = 36)
Exclusion of specific services	90.43 (*n* = 104)	96.12 (*n* = 99)	90.31 (*n* = 205)	95.87 (*n* = 116)	97.05 (*n* = 33)	82.99 (*n* = 39)	85.47 (*n* = 100)	93.62 (*n* = 44)
Did a client ever break a rule?	70.43 (*n* = 81)	57.28 (*n* = 59)	65.64 (*n* = 149)	41.32 (*n* = 50)	55.88 (*n* = 19)	70.21 (*n* = 33)	43.6 (*n* = 51)	51.06 (*n* = 24)
Exit wish	62.61 (*n* = 72)	26.21 (*n* = 27)	45.37 (*n* = 103)	20.66 (*n* = 25)	29.41 (*n* = 10)	53.19 (*n* = 25)	30.77 (*n* = 36)	34.04 (*n* = 16)
Mean amount of work days per week (SD)	5.23 (1.90)	3.66 (2.21)	4.05 (2.20)	4.84 (2.16)	3.41 (1.84)	4.19 (1.9)	4 (2.26)	4.54 (2.24)
**Number of clients**								
< 5 per day	45.2 (*n* = 52)	37.9 (*n* = 39)	42.7 (*n* = 97)	20.7 (*n* = 25)	41.2 (*n* = 14)	48.9 (*n* = 23)	48.7 (*n* = 57)	27.7 (*n* = 13)
>5 per day	12.2 (*n* = 14)	7.8 (*n* = 8)	8.4 (*n* = 19)	11.6 (*n* = 14)	17.6 (*n* = 6)	21.3 (*n* = 10)	6.0 (*n* = 7)	6.4 (*n* = 3)
< 5 per week	13.9 (*n* = 16)	18.4 (*n* = 19)	13.7 (*n* = 31)	7.4 (*n* = 9)	5.9 (*n* = 2)	8.5 (*n* = 4)	16.2 (*n* = 19)	19.1 (*n* = 9)
>5 per week	18.3 (*n* = 21)	9.7 (*n* = 10)	12.3 (*n* = 28)	17.4 (*n* = 21)	11.8 (*n* = 4)	10.6 (*n* = 5)	10.3 (*n* = 12)	14.9 (*n* = 7)
< 5 per month	3.5 (*n* = 4)	19.4 (*n* = 20)	13.2 (*n* = 30)	13.2 (*n* = 16)	8.8 (*n* = 3)	4.3 (*n* = 2)	6.8 (*n* = 8)	12.8 (*n* = 6)
>5 per month	0.9 (*n* = 1)	2.9 (*n* = 3)	1.8 (*n* = 4)	1.7 (*n* = 2)	2.9 (*n* = 1)	0	1.7 (*n* = 2)	0
N.A.	6.1 (*n* = 7)	3.9 (*n* = 4)	7.5 (*n* = 17)	28.1 (*n* = 34)	11.8 (*n* = 4)	6.4 (*n* = 3)	9.4 (*n* = 11)	19.1 (*n* = 9)

### 3.1 How is sex work setting associated with quality of life, mental health, and working conditions among sex workers?

#### 3.1.1 Is sex work setting a predictor of quality of life (QoL)?

We first examined the association between workplace settings and both the mental and physical components of QoL (see Table 2). The model output of the linear mixed models can be seen in Supplementary file S4, while model diagnostics are provided in Supplementary file S2.

**Table 2:** Physical and mental component of quality of life.

**Physical component of QoL score**							**Mental component of QoL score**					
**Term**	**Estimate**	**Std. error**	**Statistic**	***p*** **-value**	**95 % CI (lower)**	**95 % CI (upper)**	**Estimate**	**Std. error**	**Statistic**	***p*** **-value**	**95 % CI (lower)**	**95 % CI (upper)**
**Unadjusted model**												
(Intercept)	52.9	0.903	58.6	**< 0.001**	51.1	54.6	45.40	1.19	38.04	**< 0.001**	43.05	47.74
Car/Street	−3.41	1.01	−3.38	**0.0007**	−5.39	−1.43	−3.26	1.33	−2.44	**0.02**	−5.88	−0.64
Diverse/Escort	0.773	1.05	0.733	0.46	−1.29	2.84	0.23	1.39	0.17	0.87	−2.51	2.97
Client's apartment/Hotel	−1.50	0.966	−1.56	0.12	−3.40	0.390	−3.52	1.28	−2.76	**0.006**	−6.04	−1.01
Online	−0.849	0.984	−0.863	0.39	−2.78	1.08	−0.75	1.30	−0.58	0.56	−3.31	1.80
Club	−1.88	1.55	−1.22	0.22	−4.92	1.15	−6.04	2.05	−2.95	**0.003**	−10.07	−2.02
Brothel	−1.26	1.37	−0.916	0.36	−3.95	1.43	0.98	1.82	0.54	0.59	−2.60	4.55
Studio	−0.456	0.970	−0.470	0.63	−2.36	1.44	2.94	1.28	2.30	0.02	0.42	5.46
Own apartment	0.0823	1.37	0.0601	0.95	−2.60	2.76	−1.65	1.81	−0.91	0.36	−5.20	1.91
**Adjusted model**												
(Intercept)	48.39	9.92	4.88	**< 0.001**	28.43	68.36	62.67	14.44	4.34	**< 0.001**	33.59	91.74
Working days per week	−0.18	0.51	−0.36	0.72	−1.21	0.85	0.49	0.74	0.66	0.51	−1.01	1.99
Burden activity	−2.23	1.49	−1.49	0.14	−5.24	0.77	−2.78	2.18	−1.28	0.2	−7.16	1.60
Burden working hours	2.34	1.09	2.14	**0.04**	0.14	4.54	0.62	1.59	0.39	0.69	−2.58	3.83
Burden circumstances	−2.56	1.26	−2.03	**< 0.05**	−5.11	−0.02	−0.38	1.84	−0.21	0.83	−4.09	3.32
Burden financial exploitation	0.12	1.10	0.11	0.91	−2.10	2.33	−0.38	1.60	−0.23	0.82	−3.60	2.85
Burden coercion	−1.73	1.30	−1.33	0.19	−4.36	0.90	2.08	1.90	1.10	0.28	−1.74	5.91
Burden violence	−3.86	1.22	3.15	**< 0.003**	1.40	6.32	−1.11	1.78	−0.62	0.54	−4.70	2.48
Burden demands	−3.60	1.38	−2.60	**0.01**	−6.37	−0.82	−1.42	2.01	−0.71	0.48	−5.47	2.63
Burden double life	1.48	0.99	1.49	0.14	−0.52	3.47	−0.79	1.44	−0.55	0.59	−3.69	2.11
Burden relationship	−2.98	1.29	−2.30	**0.03**	−5.58	−0.37	−3.12	1.88	−1.66	0.1	−6.91	0.67
Burden financial dependence	1.60	1.39	1.14	0.26	−1.21	4.40	−0.14	2.03	−0.07	0.95	−4.22	3.95
Burden arrest	−0.15	1.20	−0.12	0.9	−2.57	2.28	2.38	1.75	1.36	0.18	−1.15	5.91
Burden sexual difficulties	−0.61	1.10	−0.55	0.58	−2.82	1.60	0.83	1.60	0.52	0.6	−2.39	4.05
Burden guilt or shame	2.34	1.12	2.08	**0.04**	0.08	4.59	−0.37	1.63	−0.23	0.82	−3.66	2.92
Burden health	−2.94	1.16	−2.53	**0.01**	−5.28	−0.60	−3.23	1.69	−1.91	0.06	−6.64	0.17
Wellbeing in sex work	−0.06	0.83	−0.07	0.94	−1.74	1.62	0.42	1.21	0.34	0.73	−2.02	2.86
Experience clients	−0.57	0.74	−0.78	0.44	−2.05	0.91	−0.32	1.07	−0.30	0.77	−2.48	1.83
Income monthly	1.15	1.29	0.89	0.38	−1.44	3.74	−0.28	1.88	−0.15	0.88	−4.05	3.50
Car/Street	6.35	3.14	2.02	**< 0.05**	0.03	12.66	2.57	4.57	0.56	0.58	−6.63	11.76
Diverse/Escort	0.86	3.33	0.26	0.8	−5.83	7.55	−6.30	4.84	−1.30	0.2	−16.05	3.45
Client's apartment/Hotel	2.37	2.17	1.09	0.28	−1.99	6.74	0.38	3.16	0.12	0.9	−5.98	6.73
Online	−0.95	2.72	−0.35	0.72	−6.42	4.51	−0.39	3.96	−0.10	0.92	−8.35	7.58
Club	0.08	4.88	0.02	0.99	−9.74	9.89	8.34	7.10	1.17	0.25	−5.95	22.64
Brothel	0.15	3.24	0.05	0.96	−6.36	6.66	2.39	4.71	0.51	0.61	−7.09	11.87
Studio	1.08	2.90	0.37	0.71	−4.76	6.92	−5.00	4.23	−1.18	0.24	−13.51	3.50
Own apartment	−3.27	2.66	−1.23	0.22	−8.62	2.07	−3.47	3.87	−0.90	0.37	−11.25	4.32
Migration Background	9.51	6.03	1.58	0.12	−2.63	21.65	1.22	8.78	0.14	0.89	−16.46	18.90
Not holding German citizenship	3.72	4.56	0.82	0.42	−5.45	12.89	−7.39	6.63	−1.11	0.27	−20.74	5.97
Lower secondary school certificate	−3.12	3.01	−1.04	0.3	−9.18	2.93	5.64	4.38	1.29	0.2	−3.17	14.45
Intermediate secondary school leaving certificate	6.20	3.07	2.02	**< 0.05**	0.02	12.38	4.18	4.47	0.94	0.35	−4.82	13.18
Completed apprenticeship	6.19	4.49	1.38	0.17	−2.84	15.22	−0.28	6.53	−0.04	0.97	−13.43	12.88
Entrance qualification for universities of applied science (“Fachabitur”, “Fachhochschulreife”)	−1.37	11.06	−0.12	0.9	−23.64	20.89	18.21	16.11	1.13	0.26	−14.22	50.63
University entrance qualification. high school degree (“Abitur”)	−0.40	3.68	−0.11	0.91	−7.81	7.01	1.89	5.36	0.35	0.73	−8.90	12.69
University degree	1.23	3.17	0.39	0.7	−5.15	7.60	3.91	4.61	0.85	0.4	−5.38	13.20
Other degree	6.06	11.30	0.54	0.6	−16.68	28.81	−10.58	16.46	−0.64	0.52	−43.71	22.54
Not being homelessness	11.34	2.10	5.40	**< 0.001**	7.11	15.56	2.36	3.06	0.77	0.44	−3.79	8.52
Not having children	−4.60	2.36	−1.95	0.06	−9.34	0.14	−3.79	3.43	−1.10	0.28	−10.69	3.12
No stable relationship	−5.91	2.12	−2.78	**0.008**	−10.18	−1.64	−2.37	3.09	−0.77	0.45	−8.59	3.85
**Services offered**												
Vaginal intercourse	−1.36	3.33	−0.41	0.68	−8.07	5.34	−1.63	4.85	−0.34	0.74	−11.40	8.13
Anal intercourse	−0.28	2.44	−0.11	0.91	−5.19	4.64	4.51	3.55	1.27	0.21	−2.64	11.67
Oral sex	1.68	2.72	0.62	0.54	−3.79	7.14	−1.47	3.95	−0.37	0.71	−9.43	6.49
Others	0.32	3.01	0.11	0.92	−5.74	6.38	3.70	4.38	0.84	0.4	−5.13	12.52
Not applicable	2.55	7.23	0.35	0.73	−12.01	17.10	15.46	10.53	1.47	0.15	−5.74	36.66

All bolded values correspond to p-values below 0.05.

##### 3.1.1.1 Mental component of QoL

In the model predicting the mental component, working in the street and/or in a car [β = −3.26, 95% CI (−5.88, −0.64), *p* = 0.02], working in a hotel or the client's apartment [β = −3.52, 95% CI (−6.04, −1.01), *p* = 0.006], as well as in a club, was associated with significantly lower mental QoL [β = −6.04, 95% CI (−10.07, −2.02), *p* = 0.003], compared to no involvement in those places. Working in a studio was linked to an increase (β = 2.94, 95% CI [0.42, 5.46], *p* = 0.02) in QoL. After controlling for socio-demographic cofactors and perceived burden related to sex work, no work setting and none of the covariates showed a significant effect. After the Bonferroni correction, all results remained significant. The unadjusted model explained 8.6% of the variance (*R*^2^), while the adjusted model explained 61.3% (see Supplementary file S3).

##### 3.1.1.2 Physical component of QoL

For the physical component of the quality of life, the only significant setting effect emerged for working in the street and/or a car, with those working in this setting showing lower physical QoL [β = −3.41, CI (−5.39, −1.43), *p* < 0.001]. When the additional covariates were added to the physical score model, the street setting was not significant after the Bonferroni correction. In the adjusted and Bonferroni-corrected model, not being homeless [β = 11.33, 95% CI (15.56, 7.11), *p* < 0.001] was positively associated with the physical component of QoL, while burden related to violence, special demands by clients, relationship difficulties, health issues, and not having a stable relationship were significant predictors of worse QoL. The initial ordinary least squares model demonstrated a violation of the residual normality assumption (Shapiro–Wilk *p* < 0.001). To ensure the validity and robustness of the parameter estimates, the final model was estimated using a robust linear model. The robust regression model confirmed the initial findings, and the adjusted estimates are reported. While the unadjusted model explained 3.7% of the overall variance (*R*^2^), the adjusted model explained 75%.

#### 3.1.2 Is sex work setting a predictor of mental diseases?

A descriptive overview of the disease prevalence across work settings can be found in Table 3. The models for all eight outcomes showed statistical health (see Supplementary file S2). Setting was generally a weak predictor of mental diseases. The addiction model had the highest explanatory power (McFadden's pseudo-*R*^2^ = 0.15) while the somatization model showed a weak fit (pseudo-*R*^2^ = 0.02).

**Table 3:** One-year prevalence of mental diseases (according to ICD-10 and DSM-5 criteria) across sex work settings.

**Work setting**	**Agoraphobia**	**Anorexia nervosa**	**Bulimia nervosa/Binge eating disorder**	**Generalized anxiety disorder (GAD)**	**Hypersomnia**	**Insomnia**	**Internet use disorder/Internet addiction**	**Illness anxiety disorder**	**Body dysmorphia**	**Major depression**	**Manic episode/Hypomanic episode**	**Persistent depressive disorder**	**Post-traumatic stress disorder (PTSD)**	**Panic disorder**	**Gambling disorder**	**Substance use disorder**	**Obsessive-compulsive disorder (OCD)**	**Somatization disorder**	**Social anxiety disorder**	**Specific phobia**
Car/Street	14.8% (*n* = 17)	0.9% (*n* = 1)	6.1% (*n* = 7)	16.5% (*n* = 19)	7% (*n* = 8)	13.9% (*n* = 16)	0% (*n* = 0)	2.6% (*n* = 3)	5.2% (*n* = 6)	24.3% (*n* = 28)	4.3% (*n* = 5)	10.4% (*n* = 12)	19.1% (*n* = 22)	22.6% (*n* = 26)	7.8% (*n* = 9)	36.5% (*n* = 42)	6.1% (*n* = 7)	4.3% (*n* = 5)	11.3% (*n* = 13)	13% (*n* = 15)
Clie*n*t's apartme*n*t/ Hotel	13.2% (*n* = 30)	2.2% (*n* = 5)	7.9% (*n* = 18)	17.6% (*n* = 40)	6.6% (*n* = 15)	12.8% (*n* = 29)	0.4% (*n* = 1)	1.8% (*n* = 4)	7.5% (*n* = 17)	26.4% (*n* = 60)	8.4% (*n* = 19)	11.5% (*n* = 26)	20.7% (*n* = 47)	22.5% (*n* = 51)	4% (*n* = 9)	29.1% (*n* = 66)	7.5% (*n* = 17)	4.4% (*n* = 10)	12.8% (*n* = 29)	12.3% (*n* = 28)
Diverse/ Escort	8.7% (*n* = 9)	2.9% (*n* = 3)	10.7% (*n* = 11)	22.3% (*n* = 23)	8.7% (*n* = 9)	10.7% (*n* = 11)	1% (*n* = 1)	2.9% (*n* = 3)	11.7% (*n* = 12)	21.4% (*n* = 22)	6.8% (*n* = 7)	10.7% (*n* = 11)	22.3% (*n* = 23)	15.5% (*n* = 16)	0% (*n* = 0)	22.3% (*n* = 23)	4.9% (*n* = 5)	3.9% (*n* = 4)	15.5% (*n* = 16)	13.6% (*n* = 14)
Brothel	6.4% (*n* = 3)	2.1% (*n* = 1)	6.4% (*n* = 3)	17% (*n* = 8)	0% (*n* = 0)	10.6% (*n* = 5)	0% (*n* = 0)	2.1% (*n* = 1)	4.3% (*n* = 2)	17% (*n* = 8)	6.4% (*n* = 3)	6.4% (*n* = 3)	19.1% (*n* = 9)	17% (*n* = 8)	2.1% (*n* = 1)	8.5% (*n* = 4)	6.4% (*n* = 3)	2.1% (*n* = 1)	10.6% (*n* = 5)	4.3% (*n* = 2)
Club	5.9% (*n* = 2)	8.8% (*n* = 3)	8.8% (*n* = 3)	23.5% (*n* = 8)	8.8% (*n* = 3)	14.7% (*n* = 5)	2.9% (*n* = 1)	2.9% (*n* = 1)	11.8% (*n* = 4)	29.4% (*n* = 10)	8.8% (*n* = 3)	8.8% (*n* = 3)	29.4% (*n* = 10)	29.4% (*n* = 10)	0% (*n* = 0)	26.5% (*n* = 9)	20.6% (*n* = 7)	2.9% (*n* = 1)	20.6% (*n* = 7)	14.7% (*n* = 5)
O*n*li*n*e	16.5% (*n* = 20)	1.7% (*n* = 2)	7.4% (*n* = 9)	22.3% (*n* = 27)	10.7% (*n* = 13)	14.9% (*n* = 18)	1.7% (*n* = 2)	0.8% (*n* = 1)	7.4% (*n* = 9)	28.1% (*n* = 34)	8.3% (*n* = 10)	14% (*n* = 17)	18.2% (*n* = 22)	18.2% (*n* = 22)	1.7% (*n* = 2)	16.5% (*n* = 20)	3.3% (*n* = 4)	5% (*n* = 6)	20.7% (*n* = 25)	13.2% (*n* = 16)
Ow*n* apartme*n*t	8.5% (*n* = 4)	2.1% (*n* = 1)	19.1% (*n* = 9)	23.4% (*n* = 11)	14.9% (*n* = 7)	19.1% (*n* = 9)	0% (*n* = 0)	2.1% (*n* = 1)	14.9% (*n* = 7)	29.8% (*n* = 14)	14.9% (*n* = 7)	14.9% (*n* = 7)	31.9% (*n* = 15)	19.1% (*n* = 9)	2.1% (*n* = 1)	29.8% (*n* = 14)	10.6% (*n* = 5)	6.4% (*n* = 3)	23.4% (*n* = 11)	17% (*n* = 8)
Studio	7.7% (*n* = 9)	3.4% (*n* = 4)	5.1% (*n* = 6)	10.3% (*n* = 12)	2.6% (*n* = 3)	12% (*n* = 14)	0% (*n* = 0)	1.7% (*n* = 2)	6.8% (*n* = 8)	21.4% (*n* = 25)	9.4% (*n* = 11)	8.5% (*n* = 10)	17.1% (*n* = 20)	13.7% (*n* = 16)	0.9% (*n* = 1)	13.7% (*n* = 16)	5.1% (*n* = 6)	3.4% (*n* = 4)	7.7% (*n* = 9)	9.4% (*n* = 11)

##### 3.1.2.1 Anxiety disorders

In the model for anxiety disorders, several significant workplace associations emerged (see Supplementary file S4). Working in a hotel and/or client's apartment was positively associated with anxiety disorders [OR = 2.54, 95% CI (1.45, 4.43), *p* = 0.001], as was working online [OR = 2.7, 95% CI (1.53, 4.74), *p* ≤ 0.001] and in a club [OR = 2.74, 95% CI (1.12, 6.68), *p* ≤ 0.03]. Working in a studio demonstrated a significant protective effect [OR = 0.48, 95% CI (−0.27, −0.85), *p* = 0.01]. After the Bonferroni correction, club and studio did not remain significant.

##### 3.1.2.2 Affective disorders

In the model for affective disorders, several significant workplace associations emerged. Working in a hotel or client's apartment was positively associated with affective disorders [OR = 3.33, 95% CI (1.70, 6.49), *p* < 0.001], as was working online [OR = 3.71, 95% CI (1.87, 7.39), *p* < 0.001] and in a club [OR = 2.86, 95% CI (1.00, 8.14), *p* < 0.05]. By contrast, working as an escort demonstrated a significant protective effect [OR = 0.46, 95% CI (0.22, 0.96), *p* = 0.04]. After the Bonferroni correction, club and escort settings did not remain significant.

##### 3.1.2.3 Addiction

In the addiction model, several significant workplace associations emerged. Working on the street or in a car was positively associated with substance-use disorders [OR = 2.9, 95% CI (1.62, 5.19), *p* < 0.001], as was working in a hotel or client's apartment [OR = 4.51, 95% CI (2.27, 8.95), *p* < 0.001] and in a club [OR = 2.77, 95% CI (1.10, 6.98), *p* = 0.03]; however, the latter did not remain significant after Bonferroni correction.

##### 3.1.2.4 Obsessive–compulsive disorders (OCD)

Working in a club was positively associated with OCD [OR = 4.92, 95% CI (1.99, 12.19), *p* = 0.001].

##### 3.1.2.5 Eating disorders

Working as an escort was positively associated with eating disorders [OR = 2.61, 95% CI (1.06, 6.45), *p* = 0.04], as was working in one's own apartment [OR = 4.15, 95% CI (1.54, 11.15), *p* = 0.005]. Only the own apartment association remained significant after the Bonferroni correction.

##### 3.1.2.6 PTSD

In the PTSD model, working in one's own apartment was positively associated with PTSD [OR = 2.54, 95% CI (1.19, 5.41), *p* = 0.02], which was not significant after the Bonferroni correction.

##### 3.1.2.7 Sleep disorders, somatization

No significant associations were found for sleep disorders and somatization.

#### 3.1.3 Is sex work setting a predictor of working conditions?

The model output of the models across this section can be seen in Supplementary file S5.

##### 3.1.3.1 Burden variables

The analysis of perceived burden began with an initial proportional odds assumption check. The Brant test revealed that the proportional odds assumption was violated for four burden outcomes (work-related strain, burden due to STDs, financial exploitation, and client demands). To correct this, a partial proportional odds model was utilized. Crucially, the model for burden due to STDs subsequently failed diagnostics due to the Hauck–Donner effect (complete separation) and was therefore excluded from interpretation due to unreliable results; full diagnostic details are available in Supplementary file S2.

Working on the street [β = −1.03, 95% CI (−1.48, −0.58), *p* < 0.001] and in hotels [β = −0.76, 95% CI (−1.23, −0.29), *p* = 0.002] was associated with lower odds of reporting no burden at all due to the work itself, indicating that these sex workers experienced higher work-related strain. For burden related to working conditions, the ordinal regression model indicated that working on the street was associated with lower odds of reporting no burden [β = −1.72, 95% CI (−2.17, −1.26), *p* < 0.001], suggesting a higher overall burden, while working online and in studios [both, β = 0.52, 95% CI (0.08, 0.96), *p* = 0.02] was associated with higher odds of reporting no burden. Online and studio settings, however, did not remain significant after the Bonferroni correction.

Street-based work was also associated with a higher burden related to financial exploitation, with significantly lower odds of reporting no burden at all (β = −1.26 to −1.11, all *p* < 0.001). The Escort category showed a positive association with reporting no burden due to financial exploitation [β = 0.74, 95% CI (0.21, 1.27), *p* = 0.006]. Hotel-based work [β = −0.58, 95% CI (−1.04, −0.11), *p* = 0.014] was again associated with higher burden. Only the street setting remained significant after *p*-value correction. For burden related to coercion, working on the street was associated with lower odds of reporting no burden [β = −0.81, 95% CI (−1.44, −0.17), *p* = 0.01], indicating a higher overall burden. In contrast, working as an escort was linked to higher odds of reporting no burden [β = 1.07, 95% CI (0.21, 1.92), *p* = 0.01]. Neither of the findings remained significant after Bonferroni correction. For burden related to violence, working on the street was associated with lower odds of reporting no burden [β = −0.85, 95% CI (−1.33, −0.38), *p* < 0.001], indicating a higher perceived burden. Escort work was associated with higher odds of reporting no burden [β = 0.65, 95% CI (0.09, 1.21), *p* = 0.02], suggesting a lower perceived burden. Working in hotels [β = −0.66, 95% CI (−1.16, −0.17), *p* = 0.009] or clubs [β = −0.83, 95% CI (−1.59, −0.07), *p* = 0.03] was also linked to higher burden. Repeatedly, only the street setting remained significant.

The Car/Street setting was also associated with a significantly increased likelihood of reporting higher burden due to client demands. The odds of reporting no burden at all were highly decreased [β = −1.37, 95% CI (−2.08, −0.66), *p* < 0.001]. For burden related to relationship difficulties, working as an escort [β = −0.53, 95% CI (−0.98, −0.08), *p* = 0.02] and in hotels [β = −0.68, 95% CI (−1.11, −0.24), *p* = 0.002] was associated with higher burden, whereas working online showed lower burden [β = 0.51, 95% CI (0.07, 0.96), *p* = 0.03]. Only the hotel setting remained significant. For burden related to sexual difficulties, only working on the street or in a car was associated with higher burden [β = −0.78, 95% CI (−1.24, −0.32), *p* = 0.001].

**Figure 2:**
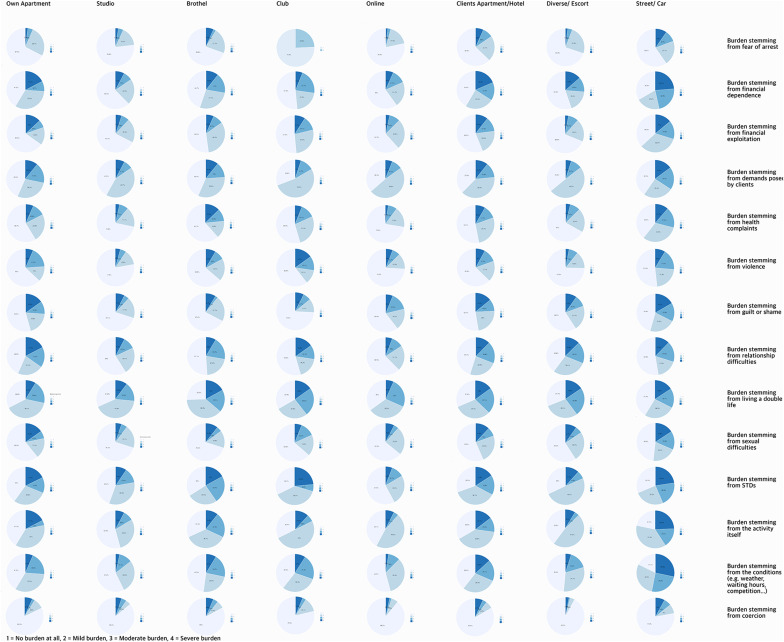
Descriptive overview of perceived burden across sex work settings.

For burden related to financial dependence, working on the street [β = −0.92, 95% CI (−1.36, −0.48), *p* < 0.001] or in hotels [β = −0.89, 95% CI (−1.32, −0.46), *p* < 0.001] was associated with higher burden, whereas working in a studio was associated with lower burden [β = 0.64, 95% CI (0.2, 1.09), *p* = 0.005]. The latter did not remain significant after Bonferroni correction. For burden related to fear of arrest, working in hotels or the client's apartment [β = −1.20, 95% CI (−1.75, −0.65), *p* < 0.001] was associated with higher burden. For burden related to guilt and shame, working on the street [β = −0.56, 95% CI (−1.01, −0.11), *p* = 0.02] and in hotels or the client's apartment [β = −0.46, 95% CI (−0.91, −0.01), *p* < 0.05] was associated with higher burden, while working online [β = 0.76, 95% CI (0.27, 1.26), *p* = 0.002] and in studios [β = 0.55, 95% CI (0.08, 1.01), *p* = 0.02] was associated with lower burden; only the online setting remained significant after Bonferroni correction. For burden related to health, working on the street [β = −1.03, 95% CI (−1.48, −0.58), *p* < 0.001] and in hotels [β = −0.76, 95% CI (−1.23, −0.29), *p* = 0.002] was associated with higher burden. The burden scores across all work settings can be seen in Figure 2.

##### 3.1.3.2 Desire to leave the industry

Working in a car or street setting was associated with a significantly higher likelihood of wanting to exit sex work [OR = 3.7, 95% CI (2.03, 6.75), *p* < 0.001]. The same applies for working in the client's apartment or a hotel [OR = 1.9, 95% CI (1.1, 3.3), *p* = 0.02] and for working in a brothel [OR = 2.56, 95% CI (1.23, 5.33), *p* = 0.01], while working as an escort [OR = 0.44, 95% CI (0.23, 0.8), *p* = 0.008] and working online was associated with a lower desire to exit [OR = 0.38, 95% CI (0.21 to 0.68), *p* = 0.001]. The model demonstrated adequate fit (see Supplementary file S2).

##### 3.1.3.3 Boundary-crossing behavior by clients

Working in a car or on the street was associated with 1.86 times higher odds of clients breaking rules [OR = 1.86, 95% CI (1.09, 3.18), *p* = 0.02]. Working in a client's apartment and/or hotel significantly increased the odds of clients breaking rules by more than twofold [OR = 2.57, 95% CI (1.53, 4.30), *p* < 0.001]. Working online significantly decreased the odds of clients breaking rules [OR = 0.57, 95% CI (0.34, 0.94), *p* = 0.03]. Working in a brothel was linked to significantly higher odds of clients breaking rules [OR = 2.27, 95% CI (1.09, 4.72), *p* = 0.03], while working in a studio significantly decreased the odds of clients breaking rules [OR = 0.48, 95% CI (0.29, 0.79), *p* = 0.004]. The model demonstrated adequate fit, as shown in Supplementary file S2.

##### 3.1.3.4 Sexual services offered and specialization

For sex workers on the street, specialization in BDSM and “other services” (not further specified) showed significantly lower odds [OR = 0.37, 95% CI (0.15, 0.95), *p* = 0.04 and OR = 0.27, 95% CI (0.11, 0.67), *p* =0.005]. In the escort category, fetish services were associated with higher odds [OR = 2.86, 95% CI (1.20, 6.78), *p* =0.02]. Among sex workers in hotels or the client's apartment, specialization as a dominatrix was linked to reduced odds [OR = 0.46, 95% CI (0.22, 0.94), *p* = 0.033]. In the studio category, BDSM specialization was associated with higher odds [β = 22.17, 95% CI (4.24, 5.09 × 10^11^), *p* < 0.001]. Nearly all individuals working in studio settings reported a specialization in BDSM services. A descriptive overview of sexual services can be found in Table 4. The model showed an adequate overall fit (see Supplementary file S2).

**Table 4:** Descriptive overview of sexual services provided across different sex work settings.

**Work setting**	**Sexual services offered**				
	**Vaginal sexual intercourse**	**Anal sexual intercourse**	**Oral sex**	**Other**	**Not applicable**
Street/Car (*n* = 115)	77.39 % (*n* = 89)	39.13% (*n* = 45)	83.48% (*n* = 96)	13.04% (*n* = 15)	2.61% (*n* = 3)
Diverse/Escort (*n* = 103)	76.70% (*n* = 79)	38.83% (*n* = 40)	79.61% (*n* = 82)	28.16% (*n* = 29)	6.80% (*n* = 7)
Client's apartment/Hotel (*n* = 227)	80.18% (*n* = 182)	42.73% (*n* = 97)	84.14% (*n* = 191)	24.67% (*n* = 56)	2.64% (*n* = 6)
Online (*n* = 121)	51.24% (*n* = 62)	32.23% (*n* = 44)	51.24% (*n* = 30)	36.36% (*n* = 34)	24.79% (*n* = 23)
Club (*n* = 34)	52.94% (*n* = 18)	32.35% (*n* = 11)	50% (*n* = 17)	41.18% (*n* = 14)	11.76% (*n* =4)
Brothel (*n* = 47)	82.98% (*n* = 39)	48.94% (*n* = 23)	85.12% (*n* = 40)	12.77% (*n* = 6)	2.13% (*n* = 1)
Studio ( *n* = 117)	62.39% (*n* = 73)	34.19% (*n* = 40)	62.39% (*n* = 73)	43.6% (*n* = 51)	9.4% (*n* = 11)
Own apartment (*n* = 47)	72.34% (*n* = 34)	48.94% (*n* = 23)	78.72% (*n* = 37)	29.79% (*n* = 14)	2.13%s (*n* = 1)

##### 3.1.3.5 Positive aspects related to sex work

**Figure 3:**
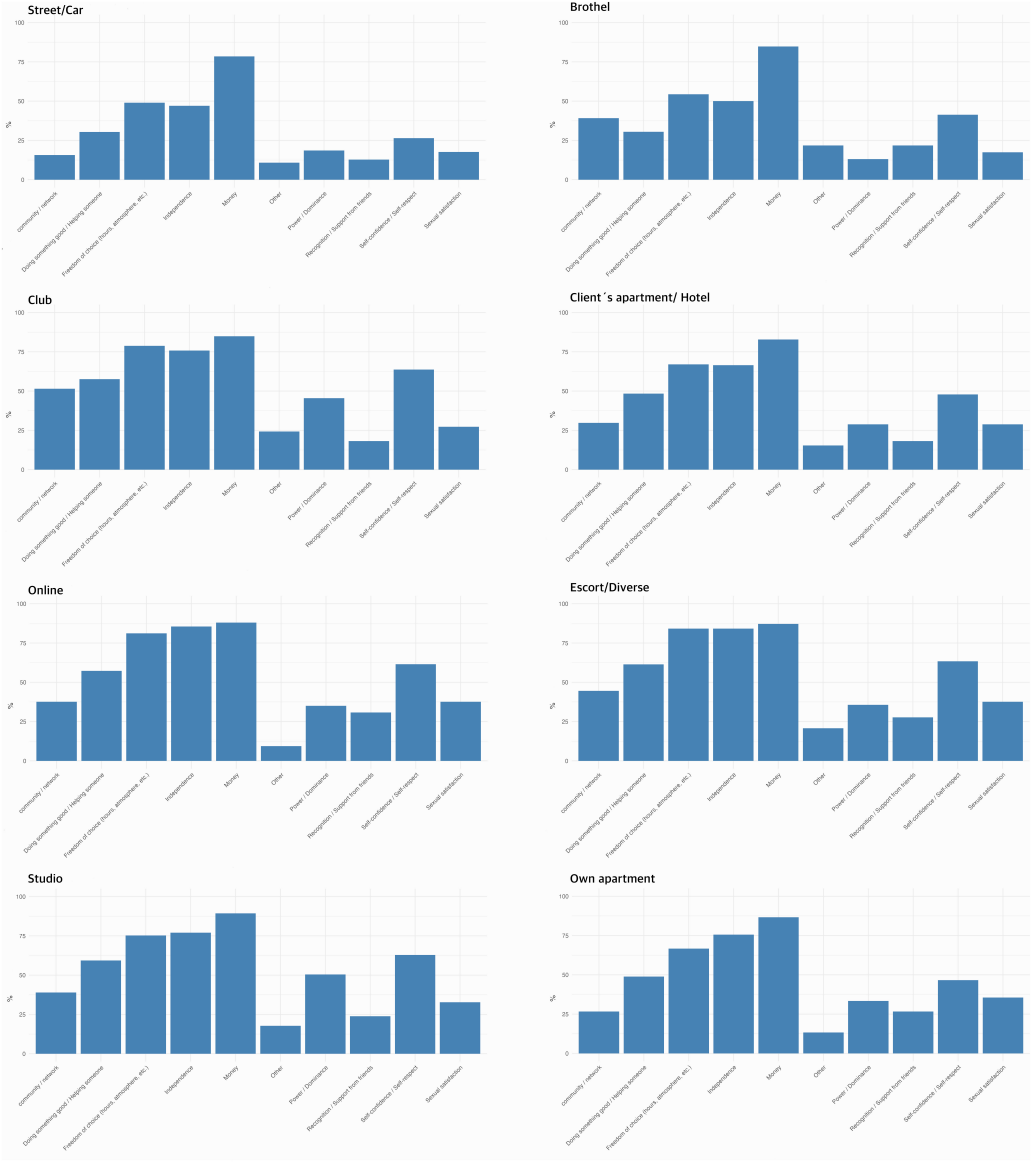
Descriptive overview of positive aspects of sex work across settings.

Please refer to Figure 3 for the descriptive figures. Stating power/dominance as a positive factor was negatively associated with car/street-based sex work [OR = 0.53, 95% CI (0.28, 1.00), *p* < 0.05]. Brothel-based work also showed a significant negative association [OR = 0.28, 95% CI (0.10, 0.77), *p* = 0.01]. In contrast, club-based sex work [OR = 2.55, 95% CI (1.11, 5.89), *p* = 0.03] and especially studio-based work [OR = 4.57, 95% CI (2.38, 8.79), *p* < 0.001] were positively associated with the factor. However, after applying the Bonferroni correction (threshold *p* < 0.005), only the effect of studio-based work remained statistically significant.

Reporting sexual satisfaction as a positive aspect was significantly associated with decreased odds [OR = 0.32, 95% CI (0.18, 0.55), *p* < 0.001] of working in the streets and/or in a car, while working online increased the odds of reporting this positive aspect [OR = 2.20, 95% CI (1.27, 3.81), *p* = 0.005]. Both effects remained significant after Bonferroni correction.

Street-based work was negatively associated with reporting doing something good or helping someone as a positive aspect of sex work [OR = 0.20, 95% CI (0.10, 0.41), *p* < 0.001]. Significant positive associations were observed for escort [OR = 3.93, 95% CI (1.85, 8.32), *p* < 0.001] and online work [OR = 2.93, 95% CI (1.55, 5.51), *p* = 0.001].

Independence was negatively associated with car/street-based work [OR = 0.29, 95% CI (0.16, 0.52), *p* < 0.001], while escort services showed a positive association [OR = 3.74, 95% CI (1.98, 7.04), *p* < 0.001]. Online [OR = 2.38, 95% CI (1.38, 4.10), *p* = 0.002] and studio work were also significantly positively associated [OR = 2.69, 95% CI (1.55, 4.67), *p* = 0.001].

Car/street-based work also showed a strong negative effect in terms of freedom of choice [working hours, atmosphere, etc.; OR = 0.32, 95% CI (0.17, 0.58), *p* < 0.001] as positive factor, while escort services [OR = 2.66, 95% CI (1.52, 4.66), *p* = 0.001], online work [OR = 2.39, 95% CI (1.42, 4.01), *p* = 0.001], and studio-based sex work [OR = 2.85, 95% CI (1.69, 4.82), *p* < 0.001] were positively associated with this aspect.

Stating self-confidence and/or self-respect as a positive aspect was again negatively associated [OR = 0.25, 95% CI (0.12, 0.49), *p* < 0.001] with car/street-based sex work. Escort services [OR = 2.39, 95% CI (1.35, 4.23), *p* = 0.003], online work [OR = 2.16, 95% CI (1.28, 3.67), *p* = 0.004] and studio-based work [OR = 2.81, 95% CI (1.65, 4.78), *p* < 0.001] showed significant positive effects.

Several predictors for reporting recognition and support from friends (common interests, experiences, protection, information flow, communication, and social support) as positive aspects were statistically significant, namely online work [OR = 2.15, 95% CI (1.21, 3.83), *p* = 0.009], studio work [OR = 2.61, 95% CI (1.46, 4.67), *p* = 0.001], and escort services [OR = 2.11, 95% CI (1.13, 3.92), *p* = 0.02]. Street-based work [OR = 0.33, 95% CI (0.16, 0.70), *p* = 0.004] was significantly associated with decreased odds of stating this aspect. Online work and escort services did not remain significant after Bonferroni correction. Finally, escort services [OR = 3.22, 95% CI (1.61, 6.44), *p* = 0.001] were positively associated with reporting money as a positive aspect. Model diagnostics indicated adequate fit across all models: McFadden's pseudo-*R*^2^ values ranged from 0.04 to 0.16, suggesting low to moderate explanatory power (see Supplementary file S2).

##### 3.1.3.6 Income categories

**Figure 4:**
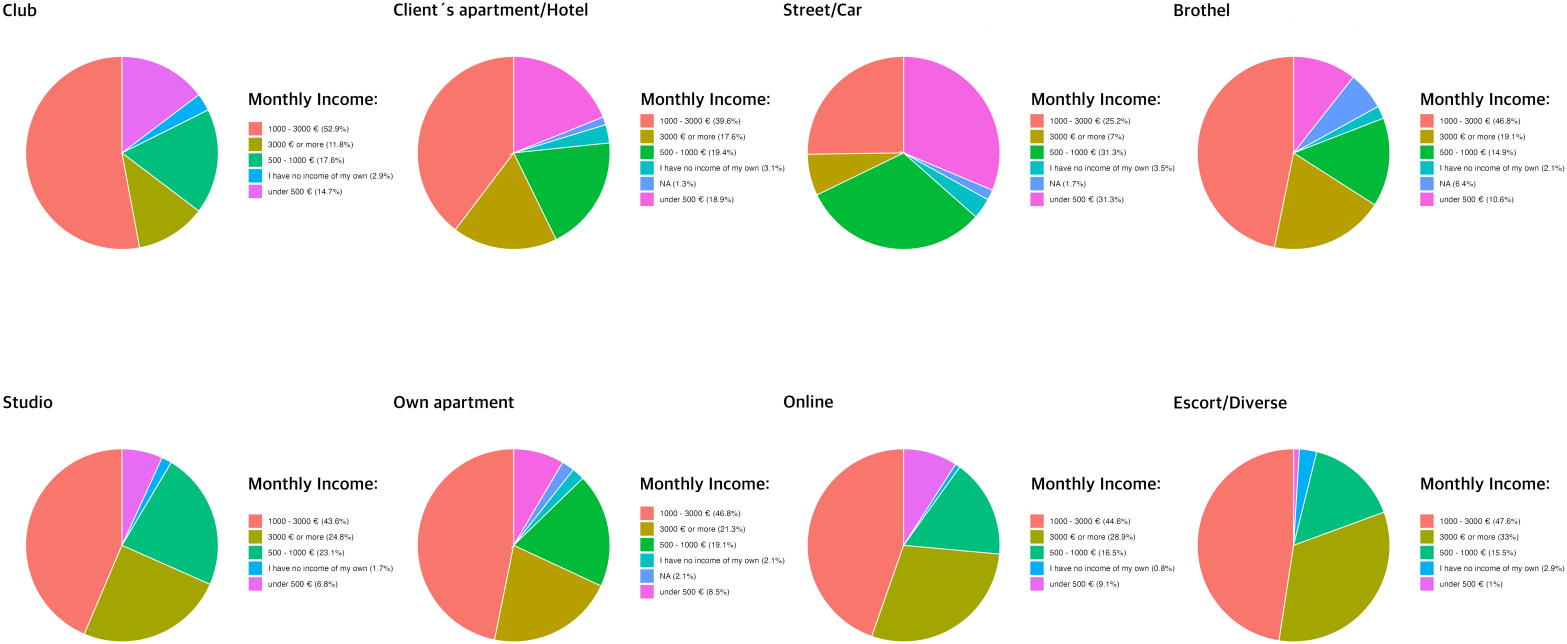
Overview of monthly income across sex work settings.

The ordinal regression model predicting monthly income showed low multicollinearity and no Hauck–Donner effect, although the Brant test revealed a violation of the parallel regression assumption for the escort predictor, necessitating the use of a partial proportional odds (PPO) model. The overall explanatory power was small (McFadden's pseudo-*R*^2^ = 0.07), with further details available in Supplementary file S2. Significant effects were observed for several work locations. Working on the street [β = −1.38, 95% CI (−1.82, −0.94), *p* < 0.001] and in hotels [β = −0.47, 95% CI (−0.89, −0.05), *p* = 0.03] was associated with lower odds of being in higher income categories. Working online [β = 0.59, 95% CI (0.17, 1.02), *p* = 0.006] was associated with higher monthly income. The escort setting was positively associated with earning €1,000–3,000 monthly [β = 0.68, 95% CI (0.23, 1.13), *p* = 0.003] and more than €3,000 monthly [β = 1.94, 95% CI (1.45, 2.44), *p* < 0.001], whereas having no personal income [β = −1.37, 95% CI (−1.97, −0.78), *p* < 0.001] was negatively associated. Please refer to Figure 4 for a descriptive overview.

### 3.2 Which factors are associated with engagement in specific sex work settings?

This analysis aimed to explore potential pathways into specific sex work settings by investigating whether socio-demographic factors, the presence of mental health diagnoses, and reasons for choosing to be a sex worker predict engagement in particular work settings.

#### 3.2.1 Do socio-demographic factors predict specific work settings?

**Figure 5:**
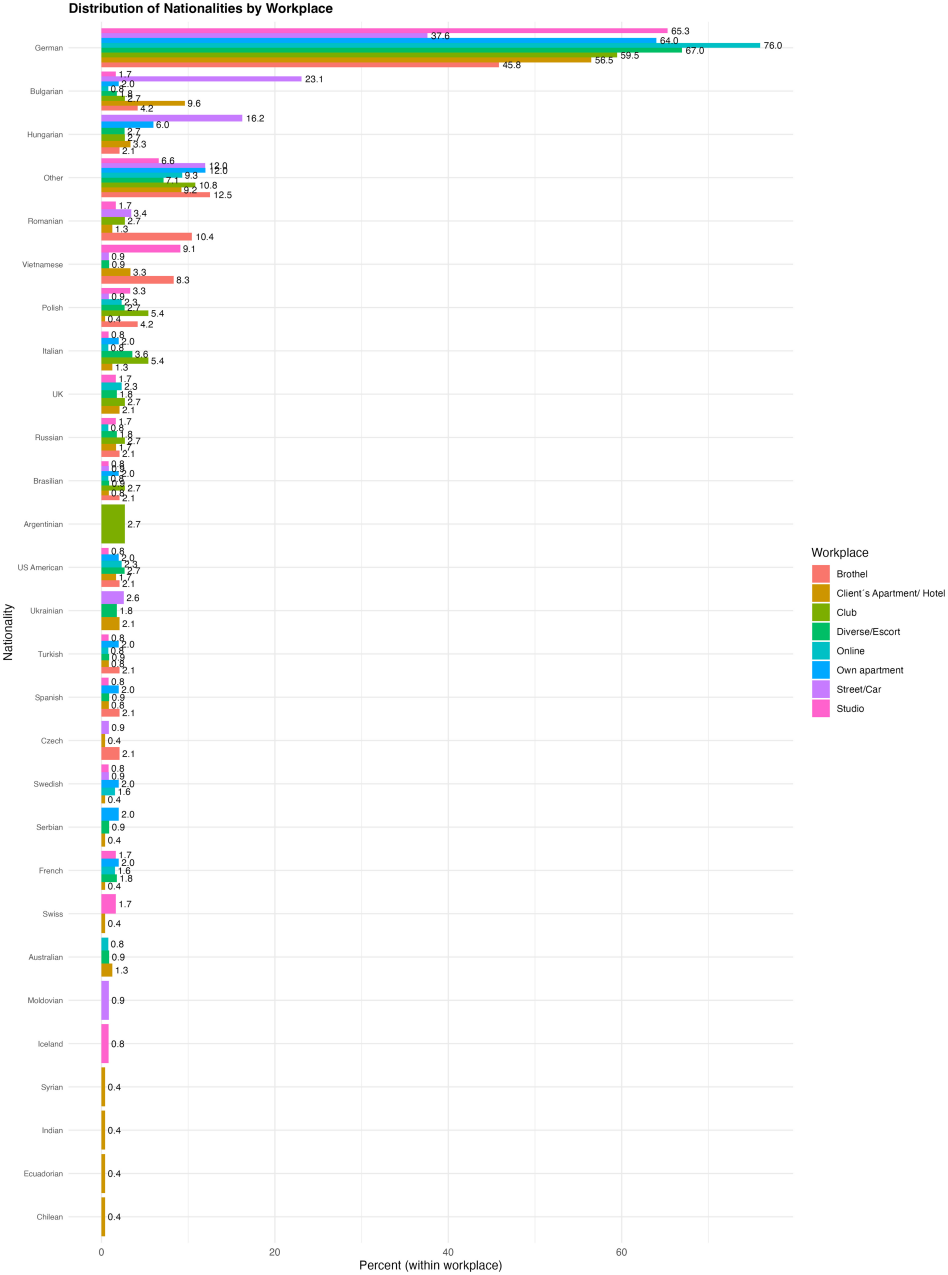
Overview of participant nationalities across sex work settings.

The model output of the fitted generalized linear mixed models across this section can be seen in Supplementary file S6. A descriptive overview of socio-demographic characteristics across work settings can be found in Table 1, while descriptive information on citizenship can be seen in Figure 5.

##### 3.2.1.1 Citizenship and migration background

Individuals who stated that at least one parent was not born in Germany showed significantly higher odds of working on the street [OR = 3.18, 95% CI (1.45, 6.98), *p* = 0.004] or in a brothel [OR = 3.42, 95% CI (1.33, 8.78), *p* = 0.01]. Conversely, for studio work, having a migration background is associated with significantly lower odds [OR = 0.5, 95% CI (0.26, 0.96), *p* = 0.04].

##### 3.2.1.2 Children, relationship status, social contacts outside of sex work, and involvement of others

Individuals with children had significantly higher odds of working in a brothel [OR = 3.15, 95% CI (1.40, 7.10), *p* = 0.006]. Not having any social contacts outside of sex work was associated with working on the street or in a car [OR = 2.33, 95% CI (1.07, 5.07), *p* = 0.03].

##### 3.2.1.3 Age at start in sex work

A higher entry age was significantly associated with lower odds of working as an escort [OR = 0.63, 95% CI (0.44, 0.82), *p* = 0.007]. None of the named factors up to this point remained significant after Bonferroni correction (corrected *p* = 0.002).

##### 3.2.1.4 Educational background

Individuals with a university degree showed significantly lower odds of working on the street [OR = 0.07, 95% CI (0.02, 0.32), *p* = 0.001]. Having completed an apprenticeship or similar vocational training was linked to significantly higher odds of working as an escort [OR = 4.52, 95% CI (1.01, 20.2), *p* < 0.05]. Additionally, having a high school degree, such as the German “Abitur,” also showed significantly higher odds for this setting [OR = 6.03, 95% CI (1.48, 24.6), *p* = 0.01]. Only the negative association of university degree and street setting remained significant after Bonferroni correction.

##### 3.2.1.5 Homelessness

Homelessness was significantly associated with higher odds of working on the street [OR = 2.82, 95% CI (1.43, 5.54), *p* = 0.002] and strongly associated with significantly higher odds of working at a hotel or the client's apartment [OR = 4.73, 95% CI (2.30, 9.73), *p* < 0.001]. In contrast, homelessness was significantly associated with lower odds of working online [OR = 0.41, 95% CI (0.19, 0.88), *p* = 0.02]. The latter did not remain significant after Bonferroni correction.

All models demonstrated strong statistical health, with low multicollinearity (VIFs < 1.61) and adequate fit confirmed by non-significant Hosmer–Lemeshow tests. The explanatory power of these models varied widely, ranging from low for the client's apartment/hotel setting to substantial for the street/car setting (pseudo-*R*^2^ = 0.33); full diagnostic details are provided in Supplementary file S2.

#### 3.2.2 Do mental diseases predict specific work settings?

The model output of the fitted generalized linear mixed models across this section can be seen in Supplementary file S7. The model exhibited robust statistical properties and an acceptable model fit with generally lower explanatory power (McFadden's pseudo-*R*^2^ < 0.08, see Supplementary file S2).

##### 3.2.2.1 Anxiety disorders

The analysis showed that anxiety disorders predicted working online [OR = 2.56, CI (1.50, 4.44), *p* = 0.001], while it was negatively associated with working in studios [OR = 0.51, CI (0.29, 0.89), *p* = 0.019], although the latter did not remain significant after Bonferroni correction.

##### 3.2.2.2 Addiction

Addiction emerged as a strong predictor for street- and/or car-based sex work, with individuals diagnosed with such disorders being significantly more likely to work in this setting compared to those without these diagnoses [OR = 4.07, 95% CI (2.16, 7.80), *p* < 0.001]. Addiction was also a significant predictor of hotel-based sex work [OR = 4.48, 95% CI (2.28, 8.62), *p* < 0.001].

##### 3.2.2.3 Affective disorders, eating disorders, PTSD, sleep disorders, somatization, and OCD

The presence of affective disorders, eating disorders, PTSD, sleep disorders, and somatization was not significantly associated with any specific work setting.

#### 3.2.3 Do self-reported reasons for sex work predict specific work settings?

**Figure 6:**
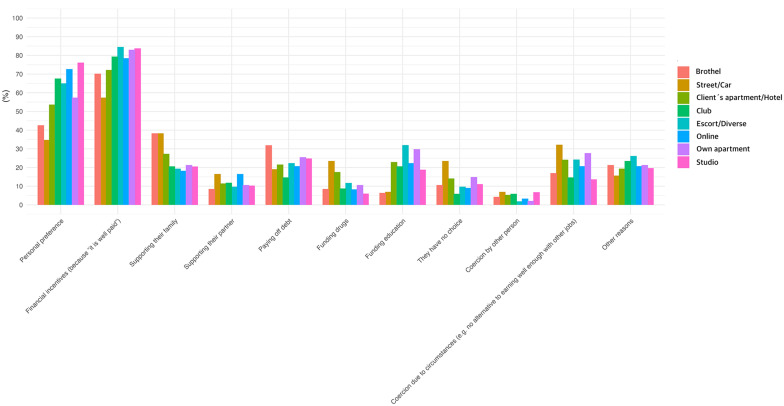
Frequencies of reported reasons for engaging in sex work across settings.

The model code can be seen in Supplementary file S8, while the output can be viewed in Table 5. A descriptive overview of the reasons for sex work can be found in Figure 6. Personal preference emerged as a positive predictor of online work [OR = 2.42, 95% CI (1.39, 4.25), *p* = 0.002] and studio-based work [OR = 3.8, 95% CI (1.99, 7.27), *p* < 0.001]. Sex workers who stated they work in this field because it is paid well were more likely to report working as escorts or in diverse places [OR = 2.9, 95% CI (1.45, 5.81), *p* = 0.003] or to work in a studio [OR = 2.61, 95% CI (1.31, 5.21), *p* = 0.006].

**Table 5:** Motivations for sex work engagement across work settings.

**Work setting**	**Term**	**Estimate (log odds)**	**Std.error**	**OR**	**95 % CI (lower)**	**95 % CI (upper)**	***p*-value**
Street/Car	(Intercept)	0.56	0.21	0.56	0.27	1.17	0.12
	Personal preference	0.38	0.13	0.38	0.19	0.76	0.01
	Financial incentives (because “it is well paid”)	0.65	0.22	0.65	0.34	1.24	0.19
	Supporting their family	1.48	0.47	1.48	0.79	2.77	0.22
	Supporting their partner	2.52	1.20	2.52	0.99	6.41	0.05
	Paying off debt	0.59	0.23	0.59	0.28	1.27	0.18
	Funding drugs	4.37	2.29	4.37	1.57	12.20	0.005
	Funding education	0.20	0.10	0.20	0.07	0.55	0.002
	They have no choice	3.44	1.84	3.44	1.20	9.83	0.02
	Coercion by other person	0.63	0.48	0.63	0.14	2.77	0.54
	Coercion due to circumstances (e.g. no alternative to earning well enough with other jobs)	2.14	0.84	2.14	0.99	4.62	0.05
	Other reasons	0.72	0.28	0.72	0.33	1.55	0.40
Escort/Diverse	(Intercept)	0.08	0.04	0.08	0.03	0.21	< 0.001
	Personal preference	1.41	0.40	1.41	0.81	2.44	0.22
	Financial incentives (because “it is well paid”)	2.90	1.03	2.90	1.45	5.81	0.003
	Supporting their family	0.54	0.17	0.54	0.29	1.02	0.06
	Supporting their partner	1.11	0.47	1.11	0.49	2.53	0.80
	Paying off debt	1.39	0.46	1.39	0.73	2.66	0.32
	Funding drugs	1.26	0.53	1.26	0.55	2.89	0.58
	Funding education	2.46	0.77	2.46	1.33	4.54	0.004
	They have no choice	0.77	0.37	0.77	0.30	2.00	0.59
	Coercion by other person	0.47	0.41	0.47	0.08	2.67	0.39
	Coercion due to circumstances (e.g. no alternative to earning well enough with other jobs)	1.91	0.67	1.91	0.96	3.79	0.06
	Other reasons	2.67	0.91	2.67	1.38	5.20	0.004
Client's apartment/ Hotel	(Intercept)	0.63	0.20	0.63	0.34	1.15	0.13
	Personal preference	0.93	0.23	0.93	0.57	1.51	0.76
	Financial incentives (because “it is well paid”)	1.50	0.41	1.50	0.87	2.56	0.14
	Supporting their family	0.81	0.21	0.81	0.49	1.36	0.43
	Supporting their partner	1.05	0.40	1.05	0.50	2.21	0.91
	Paying off debt	1.37	0.43	1.37	0.75	2.52	0.31
	Funding drugs	12.22	7.93	12.22	3.42	43.63	< 0.001
	Funding education	2.07	0.64	2.07	1.13	3.81	0.02
	They have no choice	1.41	0.64	1.41	0.58	3.44	0.45
	Coercion by other person	2.54	1.93	2.54	0.57	11.25	0.22
	Coercion due to circumstances (e.g. no alternative to earning well enough with other jobs)	1.61	0.53	1.61	0.84	3.09	0.15
	Other reasons	1.38	0.44	1.38	0.74	2.56	0.31
Online	(Intercept)	0.16	0.06	0.16	0.07	0.35	< 0.001
	Personal preference	2.43	0.69	2.43	1.39	4.25	0.002
	Financial incentives (because “it is well paid”)	1.40	0.42	1.40	0.78	2.52	0.26
	Supporting their family	0.44	0.14	0.44	0.24	0.81	0.008
	Supporting their partner	2.64	1.02	2.64	1.24	5.63	0.01
	Paying off debt	1.52	0.49	1.52	0.82	2.85	0.19
	Funding drugs	0.78	0.33	0.78	0.34	1.80	0.56
	Funding education	1.29	0.38	1.29	0.72	2.29	0.39
	They have no choice	0.85	0.39	0.85	0.34	2.10	0.72
	Coercion by other person	0.80	0.56	0.80	0.20	3.14	0.75
	Coercion due to circumstances (e.g. no alternative to earning well enough with other jobs)	1.62	0.55	1.62	0.83	3.13	0.16
	Other reasons	1.66	0.53	1.66	0.89	3.10	0.11
Club	(Intercept)	0.00	0.00	0.00	0.00	0.00	< 0.001
	Personal preference	1.38	3.21	1.38	0.02	131.30	0.89
	Financial incentives (because “it is well paid”)	1.70	4.38	1.70	0.01	267.57	0.84
	Supporting their family	0.75	1.98	0.75	0.00	135.64	0.91
	Supporting their partner	1.19	3.91	1.19	0.00	739.14	0.96
	Paying off debt	0.88	2.66	0.88	0.00	323.97	0.97
	Funding drugs	1.07	3.87	1.07	0.00	1,291.34	0.99
	Funding education	1.09	2.73	1.09	0.01	147.47	0.97
	They have no choice	0.48	2.06	0.48	0.00	2,095.60	0.86
	Coercion by other person	3.51	16.26	3.51	0.00	31,188.08	0.79
	Coercion due to circumstances (e.g. no alternative to earning well enough with other jobs)	0.71	2.25	0.71	0.00	364.86	0.91
	Other reasons	1.82	4.47	1.82	0.02	225.05	0.81
Brothel	(Intercept)	0.00	0.00	0.00	0.00	0.00	< 0.001
	Personal preference	0.53	0.97	0.53	0.01	19.84	0.73
	Financial incentives (because “it is well paid”)	1.41	2.83	1.41	0.03	72.70	0.87
	Supporting their family	1.65	3.06	1.65	0.04	62.13	0.79
	Supporting their partner	0.61	1.95	0.61	0.00	319.34	0.88
	Paying off debt	2.77	5.27	2.77	0.07	115.39	0.59
	Funding drugs	0.69	2.14	0.69	0.00	292.49	0.91
	Funding education	0.24	0.81	0.24	0.00	190.05	0.67
	They have no choice	0.83	2.41	0.83	0.00	254.32	0.95
	Coercion by other person	0.82	3.74	0.82	0.00	6,120.71	0.97
	Coercion due to circumstances (e.g. no alternative to earning well enough with other jobs)	0.62	1.50	0.62	0.01	69.56	0.84
	Other reasons	1.38	2.99	1.38	0.02	96.41	0.88
Studio	(Intercept)	0.08	0.04	0.08	0.03	0.20	< 0.001
	Personal preference	3.80	1.26	3.80	1.99	7.27	< 0.001
	Financial incentives (because “it is well paid”)	2.61	0.92	2.61	1.31	5.21	0.006
	Supporting their family	0.61	0.19	0.61	0.33	1.14	0.12
	Supporting their partner	0.58	0.25	0.58	0.26	1.33	0.20
	Paying off debt	2.86	1.02	2.86	1.42	5.77	0.003
	Funding drugs	0.56	0.28	0.56	0.21	1.50	0.25
	Funding education	0.84	0.26	0.84	0.45	1.56	0.58
	They have no choice	2.00	0.99	2.00	0.76	5.27	0.16
	Coercion by other person	7.56	5.17	7.56	1.98	28.86	0.003
	Coercion due to circumstances (e.g. no alternative to earning well enough with other jobs)	0.43	0.18	0.43	0.19	0.96	0.04
	Other reasons	1.77	0.61	1.77	0.90	3.49	0.10

Funding drugs emerged as a positive predictor of working in a hotel or client's apartment [OR = 12.22, 95% CI (3.42, 43.63), *p* < 0.001] and in a street setting [OR = 4.37, 95% CI (1.57, 12.2), *p* = 0.005]. Persons who work in sex work to fund their education were significantly less likely to report working in the streets [OR = 0.2, 95% CI (0.07, 0.55), *p* = 0.002]. They were, however, more likely to work as escorts [OR = 2.46, 95% CI (1.33, 4.54), *p* = 0.004] and in a hotel or the client's apartment [OR = 2.07, 95% CI (1.13, 3.81), *p* = 0.019]. The latter was not significant after Bonferroni correction.

Stating to “have no other choice” was a predictor of working in the streets or in a car [OR = 3.44, 95% CI (1.2, 9.83), *p* = 0.02]. Reporting being “forced by the circumstances” [OR = 0.43, 95% CI (0.19, 0.96), *p* = 0.04] was associated with lower odds of working in a studio. Only the studio setting remained significant after Bonferroni correction.

The logistic regression models predicting sex work settings based on self-reported reasons for working were statistically sound with low multicollinearity (VIFs < 1.44) and adequate model fit across all settings (all Hosmer–Lemeshow *p* > 0.15), except for the Own Apartment setting (Hosmer–Lemeshow *p* = 0.0006). Hence, it was excluded from the final analysis and results due to poor diagnostic performance (see Supplementary file S2).

### 3.3 How are reported healthcare and support needs associated with sex work setting?

#### 3.3.1 Does work setting predict the reported needs?

**Figure 7:**
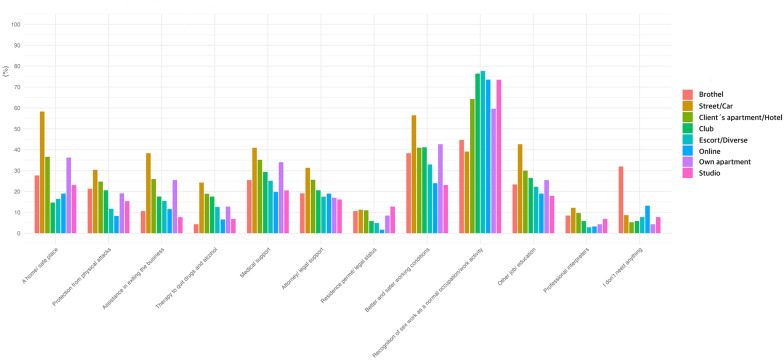
Frequencies of reported socio-medical needs across sex work settings.

A descriptive overview of the reported needs across work settings can be found in Figure 7. The model output and R code can be viewed in Supplementary file S9. Working in a street/car setting was strongly associated with a higher probability of reporting the need for a home or safe place [OR = 10.07, 95% CI (5.65, 17.96), *p* < 0.001]. Other significant positive associations included protection from physical attacks [OR = 3.6, 95% CI (1.34, 9.70), *p* = 0.01], assistance in exiting the business [OR = 5.98, 95% CI (2.26, 15.83), *p* < 0.001], therapy to quit drugs and alcohol [OR = 4.59, 95% CI (1.64, 12.83), *p* = 0.004], medical support [OR = 3.73, 95% CI (1.44, 9.63), *p* = 0.007], attorney/legal support [OR = 3.65, 95% CI (1.37, 9.68), *p* = 0.009], better and safer working conditions [OR = 6.36, 95% CI (2.49, 16.28), *p* < 0.001], and another job/education [OR = 4.31, 95% CI (1.66, 11.13), *p* = 0.003]. Recognition of sex work as a regular occupation was negatively associated [OR = 0.2, 95% CI (0.08, 0.51), *p* = 0.001]. Only “home or safe place,” “assistance in exiting the business,” “better and safer working conditions,” and “recognition of sex work as a regular occupation” remained significant.

Working as an escort was negatively associated with the need for a home or safe place [OR = 0.33, 95% CI (0.17, 0.65), *p* = 0.001], while recognition of sex work as a regular occupation was positively associated [OR = 3.5, 95% CI (1.18, 10.42), *p* = 0.02]. Only the negative association with a home or safe place remained significant.

Working in hotels or the client's apartment was positively associated with the need for a home or safe place [OR = 2.81, 95% CI (1.63, 4.86), *p* < 0.001], protection from physical attacks [OR = 3.88, 95% CI (1.45, 10.42), *p* = 0.007], assistance in exiting the business [OR = 3.16, 95% CI (1.21, 8.24), *p* = 0.02], therapy to quit drugs and alcohol [OR = 6.18, 95% CI (1.98, 19.29), *p* = 0.002], medical support [OR = 5.11, 95% CI (1.99, 13.14), *p* < 0.001], attorney/legal support [OR = 4.11, 95% CI (1.53, 10.98), *p* = 0.005], and better and safer working conditions [OR = 3.26, 95% CI (1.33, 7.97), *p* = 0.01]. Reporting that no support was needed was negatively associated [OR = 0.18, 95% CI (0.06, 0.50), *p* = 0.001]. Only “home or safe place,” “therapy to quit drugs and alcohol,” “medical support,” and “no support needed” remained significant.

Online work was negatively associated with a home or safe place [OR = 0.4, 95% CI (0.20, 0.67), *p* = 0.004], protection from physical attacks [OR = 0.27, 95% CI (0.09, 0.82), *p* = 0.02], and residence permit/legal status [OR = 0.10, 95% CI (0.02, 0.42), *p* = 0.008]. Only the negative association with a home or safe place remained significant.

Reporting that no support was needed was strongly positively associated with working in a brothel [OR = 6.71, 95% CI (2.03, 22.16), *p* = 0.007]; however, this did not remain significant after Bonferroni correction. Assistance in exiting the business was negatively associated with working in a studio [OR = 0.19, 95% CI (0.06, 0.55), *p* = 0.004], while recognition of sex work as a regular occupation was positively associated [OR = 2.69, 95% CI (1.00, 7.25), *p* < 0.05]. Only the first remained significant after Bonferroni correction. The binary logistic regression models predicting socio-medical needs based on work settings demonstrated adequate model fit (diagnostic details are available in Supplementary file S2).

## 4 Discussion

In this study, we aimed to analyze the impact that the workplace has on QoL, mental illnesses, and working conditions to identify the unique risks and positive resources that workplaces bear. In the second part, we analyzed whether socio-demographic characteristics, mental conditions, and individual reasons predicted workplace outcomes. In the last part, we analyzed whether workplaces predicted the reported needs of sex workers. In each analysis, we found significant findings that paint a unique picture of the workplace, the individuals involved, and the individual needs to improve healthcare and support situations. In the following sections, each workplace should be summarized and discussed in detail, followed by recommendations on how to improve medical and other support services for individuals involved.

### 4.1 Summary and recommendations

#### 4.1.1 Street- and/or car-based sex work

This setting predicted worse mental and physical scores for QoL. It predicted addiction and higher burden scores. It was also associated with increased rule-breaking by clients and a strong desire to exit sex work. All significant positive aspects were negatively associated with this setting. Additionally, this setting was associated with lower income. This supports the thesis that street-based work is a high-risk environment with multiple stressors and very limited positive resources, as depicted in previous publications (29).

For street- and/or car-based sex work, significant predictors included migration background, homelessness, having no social contacts beyond sex work, suffering from addiction, and having a lower educational background (6.96% reported being illiterate). An analysis of personal reasons for being involved as sex workers revealed that street- and/or car-based sex workers had significantly higher odds of reporting being involved in sex work to finance drugs and because they had no other choice. Although, in the statistical model, no significant association between street-based work and citizenship was found, it is evident from the descriptive statistics that the most frequent citizenships besides German were Bulgarian (22.7%), Hungarian (16%), and Romanian (3.36%). 2.52% held Ukrainian citizenship, followed by Moldovan, Polish, Czech, and Vietnamese citizenship (accounting for approximately 1% each). This shows that migration routes exist within and beyond Europe, with a strong focus on Eastern European countries, and include current conflict zones such as Ukraine, raising questions about how forced migration can intersect with engagement in sex work. It further supports the hypothesis that street-based sex workers are a highly vulnerable group due to several socio-demographic factors, such as language barriers, education, and other structural barriers to societal participation, as outlined above. In addition to that, several of these factors can contribute to experiencing stigma and discrimination beyond the discrimination related to sex work (65, 66). The setting was, for example, associated with several need, and the findings align with available literature (67, 68).

##### 4.1.1.1 Recommendations

A multifaceted strategy is crucial to adequately respond to the complex challenges faced by street-based sex workers. This includes expanding the housing capacities of low-barrier shelters and creating resting areas with 24/7 access. We recommend strengthening evidence-based exit and alternative livelihood programs, following approaches as outlined by Baker et al. (69). Enhancing opportunities for social networking with peers or non-sex workers could help mitigate social isolation and prevent entering street- and/or car-based sex work, since having no social contacts beyond sex work has emerged as a predictor of being involved in this setting (70). Accessible healthcare could be improved by offering free and anonymous medical check-ups alongside on-site or mobile mental health support, embedding trauma-informed counseling that addresses violence, coercion, and trafficking, as well as targeted therapies for substance-use disorder (71, 72). Peer-led outreach teams that build trust and connect sex workers, particularly those who distrust institutional providers, to essential services have proven to be valuable resources for enhancing outreach effectiveness (73, 74). Inclusive sex education should be delivered through accessible workshops on consent, safe practices, and sex workers' rights. Services must be complemented by multilingual materials, culturally sensitive information, and professional interpreters (75). Measures to combat human trafficking and other forms of violence should be strengthened in this setting (76). Finally, the results highlight the need to invest in prevention programs that begin in countries of origin, ensuring that education, economic opportunities, and information about the specific risks of sex work are available to prevent vulnerable girls and women from (involuntarily) entering street-based sex work in the first place.

#### 4.1.2 Escort services

Working as an escort predicted suffering from an eating disorder and lowered the probability of reporting affective disorders. It was linked to lower burden scores and was associated with lower odds of wanting to exit sex work, while it predicted reporting multiple positive aspects. It was associated with higher income categories and a specialization in fetish services. This shows that the setting is associated with multiple positive resources.

Educational attainment (such as having a high school degree), being involved in sex work to fund one's education, and financial incentives (because “it is well paid”) emerged as predictors of working in this setting. The specific connection between eating disorders and this work setting must be investigated further, as there is almost no literature on this topic, aside from a case study by Cooney (77). Escort work was associated with significantly lower odds of reporting the need for a place to live, while there were increased odds of reporting the need to recognize sex work as a regular occupation.

##### 4.1.2.1 Recommendations

To improve support services for escort sex workers, we recommend strengthening services that respect their autonomy and diverse reasons for engaging in this work and acknowledge the socio-economic characteristics, such as a higher educational background. The data imply that escort sex workers would benefit from recognizing this work as a legitimate occupation, for example, through targeted campaigns that highlight the diversity, agency, and informed choices of escorts. This includes training healthcare and social service providers to avoid moral judgments and to deliver non-discriminatory, confidential care, as well as supporting sex worker advocacy groups in co-creating public education initiatives and engaging in policy dialogue (78). Furthermore, accessible mental health resources should be provided to address mental conditions, such as eating disorders (71).

#### 4.1.3 Hotel-based sex work and working in the client's apartment

The setting emerged as a predictor of a lower mental component of QoL. It predicted anxiety disorders and affective disorders, as well as addiction, and was associated with a higher perceived burden related to the work itself. It was also associated with increased rule-breaking by clients, a higher desire to exit sex work, and lower income. The literature regarding hotel-based sex work is scarce, and the available publications highlight problems with violence, non-paying clients (79), and a high prevalence of STDs (80).

Financing one's education, suffering from addiction, as well as stating that one is involved in sex work to fund drugs were predictors of this setting. It was associated with a need for a home or safe place, needing assistance in exiting sex work, a need for protection from physical attacks, therapy to quit drugs, medical and legal support, better and safer working conditions, and another job/education.

Better working conditions have been described in the literature as including stronger legal protections, enforceable labor rights, and safer physical environments (81). This includes ensuring secure and hygienic workplaces, protection from violence and exploitation by clients or third parties, and the ability to negotiate services freely and safely (82). Practical improvements might include access to panic alarms, security staff, self-defense training, and clear workplace policies that prioritize sex workers' consent and autonomy (83). It must be further investigated, if and how these measures can be implemented in these settings since several factors, such as panic alarms and security staff, are not available in hotels and private apartments.

These results should be interpreted considering the moderate correlations between this setting and two other work environments: street-based sex work and working as an escort and/or in various locations (see Figure 1). This overlap makes it challenging to isolate the effects of these subgroups, as both appear to operate in hotels and/or clients' apartments. Notably, addiction emerged as a positive predictor, similar to findings in the street-based setting, while financing one's education was also identified as a predictor, consistent with the escort setting. Therefore, findings should be approached with caution, as they may pertain only to specific subsets within the broader group working in hotels and/or clients' apartments, potentially reflecting the influence of smaller subgroups. However, neither the street setting nor escort services predicted anxiety or affective disorders. Either the accumulated effects of both subgroups combined caused the significant effect for this setting, or it bears unique risks for developing anxiety and affective disorders. Further research is needed to identify where the effect stems from.

##### 4.1.3.1 Recommendations

Based on these findings, we recommend expanding support services to better reach sex workers who work in hotels or clients' apartments. Outreach efforts could be strengthened, for example, through peer-led initiatives (73, 74). The data highlight a clear need for secure housing, evidence-based exit assistance (69), and improved, safer working conditions. Further research is necessary to evaluate measures to improve working conditions as well as the efficacy of interventions and their implementation in this setting. We recommend strengthening medical services by integrating (mobile) drop-in clinics, peer-led health outreach, and tailored care pathways alongside destigmatization training for healthcare professionals (84–86).

#### 4.1.4 Online sex work

Online work was a predictor of anxiety disorders and affective disorders, while it was also associated with a lower desire to exit the industry and decreased rule-breaking by clients. The setting was positively associated with experiencing sexual satisfaction, reporting doing something good or helping someone, independence, freedom of choice, self-confidence, and recognition as positive aspects of the work. Online work was also associated with higher income categories. Previous literature reports that clients were more likely to display verbally abusive behavior given the anonymity of the internet (87). Mental burnout is also reported as a possible consequence of online work due to several reasons, such as the pressure of being online for an extensive time or the pressure to create online content while being involved in everyday activities (87). Several risks are mentioned, such as non-consensual sharing of online content or greater digital exposure that might pose safety concerns (87).

Anxiety disorders were predictors of working online. Specifically, the prevalence of social anxiety and agoraphobia tended to be higher than in other settings (see Section 3.1.2). It is plausible that individuals with these conditions might prefer an online setting instead of an in-person encounter. However, the direction of the effects has to be studied further in appropriate study designs to investigate causality. Personal preference emerged as a positive predictor of online work. Supporting their family emerged as a negative predictor, while supporting their partner was a positive predictor of online work. Possible explanations for the lower likelihood of supporting their families include that their families did not require financial support. It could also reflect strained or limited family relationships. Further research should investigate whether individuals in this group maintain regular contact with their families at all. Online sex workers tended to have a vocational baccalaureate diploma, and therefore, an intermediate educational background. The only significantly associated need was recognition of sex work as a regular occupation/work activity.

Finally, it is important to highlight that online sex work comprises a range of different work models, each with distinct structures and demands (88). These include the sale of prerecorded videos and images, live cam performances, and work as an adult film performer. The latter typically involves collaboration with directors, producers, and co-actors, and differs considerably in terms of working conditions (89). For example, adult film performer usually work only on specific shooting days but for extended hours. In contrast, cam performers tend to have more regular, scheduled working hours, during which they perform live for set durations. Creators on platforms like OnlyFans often spend a substantial amount of time producing and editing content while also maintaining ongoing communication with customers via messaging services. Given these significant differences, further research is needed to investigate how these various subgroups may be affected differently in terms of psychosocial and occupational outcomes (90).

##### 4.1.4.1 Recommendations

We recommend expanding accessible mental health support tailored to the online environment, including specialized counseling services, online forums, and trained social workers who understand the digital sex work industry's unique dynamics. In addition, targeted advice and training on privacy protection, dealing with blackmail or hackers, and safe online practices could be offered to empower workers to protect their identities and digital security (87). The data show that online sex workers would benefit from recognizing online sex work as paid labor. Given that online sex work spans various models, from live camming to prerecorded content and professional adult film production, future interventions and research should differentiate these subgroups to better tailor mental health services, workplace protections, and labor standards to their specific conditions and needs.

#### 4.1.5 Brothels

Brothel-based work did not predict QoL, mental illnesses, and generally showed no significant associations across most burden variables. Working in a brothel emerged as a predictor of higher odds of wishing to exit sex work and increased rule-breaking by clients. It was also connected to lower odds of reporting independence as a positive factor. This implies that brothel-based work offers limited positive resources (compared to other settings) while bearing increased risks such as boundary-crossing behaviors by clients.

Individuals engaged in this setting tended to have a migration background, children, and an intermediate or higher educational background. Stating “I do not need anything” was the only “need” associated with this setting. Previous research from South Africa identified that clinical on-site services in brothels could endorse positive health-seeking behavior, health awareness, and condom use, and found that clinical services offered on site are a good alternative to the provision of conventional clinical services (91). Research on working conditions in brothels remains very scarce. Most of the available literature is over 20 years old, does not stem from high-income countries, or focuses on “indoor sex work,” which comprises several settings and does not allow for further distinction (92–94). Therefore, there appears to be a significant research gap concerning brothels in high-income countries, except Australia (95–97). This may be partly due to legal factors, as sex work establishments are often regulated or prohibited differently from the act of sex work itself. Additionally, brothel workers can be difficult to access for research, since brothel owners frequently control and restrict researchers' contact with them. In this study, we contacted a large number of brothels to conduct interviews; however, only one brothel granted access to the researchers. Therefore, results must be reviewed critically (see limitations, Section 4.2).

##### 4.1.5.1 Recommendations

Further research is necessary, since our results stem from only one brothel.

#### 4.1.6 Studio-based work

Working in a studio demonstrated a significant protective effect for anxiety disorders and predicted a higher mental component score of QoL. It was negatively associated with a lower burden related to the work itself. It was also associated with decreased rule-breaking by clients and linked to a specialization in BDSM services, as indicated by previous literature (98). Reporting doing something good or helping someone, independence, freedom of choice, self-confidence and self-respect, recognition or support from friends, and money emerged as positive aspects of the work.

Not having a migration background and higher education was associated with working in a studio setting. Individuals with anxiety disorders or addiction were less likely to work in this setting. Studio work was a predictor of reporting personal preference, financial benefits (“it is well paid”), and paying off debt as personal reasons for sex work, while it was linked to a lower likelihood of reporting being forced by circumstances as a reason. Paying off debt must be viewed in the context of income and other socio-demographic factors. 24.8% of studio workers reported earning over 3,000 Euro monthly, while the majority had German citizenship (65.3 %). This indicates that paying off debt may rather be related to personal investments. O'Doherty et al. (28) found that the participants in their qualitative study, primarily professional dominatrices, viewed their work as distinct from other forms of sex work, emphasizing the specialized skills, education, and training essential for their profession. They highlighted the importance of delivering BDSM experiences safely and responsibly, requiring knowledge in administering physical pain without causing actual harm (28).

Working in a studio was associated with lower odds of reporting needing assistance in exiting the business, while it predicted reporting the need for recognition of sex work as a regular occupation/work activity.

##### 4.1.6.1 Recommendations

The data showed that studio-based sex workers would benefit from the recognition of sex work as a regular occupation/work activity. This could be achieved through destigmatizing campaigns and informative workshops for, for instance, healthcare staff and authorities.

#### 4.1.7 Own apartment

Working in this setting was a predictor of OCD, PTSD, and eating disorders. No other associations emerged across the analyses. Therefore, this setting was not as distinct as other settings, meaning that its association with various outcomes, such as mental health indicators, experiences of violence, or access to support services, was less pronounced or inconsistent across measures (no effect or opposite effects that neutralize each other). Working in one's own apartment displayed a high correlation with other work settings, such as the client's apartment, and a moderate correlation with online work, as well as escort services. This might be the reason why we did not find many significant associations across the analyses. Like the setting “hotel or client's apartment,” the lack of adequate security measures might explain the association with PTSD. However, the connection between mental disorders and working in one's own apartment needs further study.

##### 4.1.7.1 Recommendations

The setting overlaps considerably with other work contexts, particularly hotels/clients' apartments, online work, and escort services, making it difficult to isolate its unique effects. Future research should explore this subgroup further to better understand their specific working conditions and needs in order to develop more targeted support. Accessible mental health resources should be provided to address mental conditions, such as PTSD (71).

#### 4.1.8 Club

This setting was associated with significantly lower mental QoL, as well as a higher burden stemming from violence. Working in clubs was associated with a higher likelihood of reporting power and dominance as positive factors of the work. It predicted suffering from OCD. In total, this setting displayed negative associations as well as positive resources, painting a mixed picture of this working environment.

##### 4.1.8.1 Recommendations

The findings suggest that further research is necessary to investigate the link between club-based work, OCD, and to better understand possible occupational stressors or environmental conditions.

### 4.2 Limitations

Several limitations of the study design and methodology should be acknowledged. The sample is not representative due to the high fluidity of the population, which makes a representative sample practically impossible. Similarly, the findings are not representative of the specific settings as well, and while the statistical results point to significant trends, it is important to remember that there are always exceptions; for example, some street-based sex workers may not require any exit assistance, whereas some escort workers might benefit from exit support. Participants were recruited via NGOs, social media, and direct outreach, which may have led to an overrepresentation of more visible, networked, or digitally active individuals and an underrepresentation of highly marginalized sex workers, including those without internet access or affiliation with support services. One particular group that was not adequately reached was sex workers involved in human trafficking, as well as sex workers engaged in sexual assistance (see Section 1).

Additionally, we chose to focus on female sex workers due to the fact that the lived experience of male sex workers is likely to differ. To reduce heterogeneity, we therefore focused on the larger group of female sex workers. However, more research on male sex workers is necessary, as this is an often-overlooked group within the sex work environment. Furthermore, the experiences of trans^*^ women and non-binary sex workers may not have been fully captured; future research should adopt more inclusive measures of gender identity to better reflect the diversity of this population.

As a cross-sectional study, the findings only capture a snapshot in time and cannot determine causality. Associations identified between work settings, mental health, and socio-demographic variables may be bidirectional or confounded by unmeasured variables. Longitudinal data would be necessary to explore causal pathways and temporal dynamics. Because many participants work in multiple settings, we initially considered two statistical approaches: (a) standard generalized linear models (GLMs) treating each work setting as a separate predictor, and (b) mixed-effects models accounting for the correlation of multiple work settings within the same individual. However, given the cross-sectional nature of the data and the fact that all outcomes (e.g., mental health and quality of life) were measured at only a single time point, mixed-effects models were not appropriate, as they require within-person variance to separate the effect of work setting from individual characteristics. Consequently, we opted for standard GLMs. This approach was necessary to analyze the data, but it bears the risk of overrepresentation of women who work in multiple places, which should be considered when interpreting the associations between work settings and outcomes. The removal of rare diagnostic categories and underpopulated work settings, while methodologically justified, may have resulted in the loss of data from particular subgroups. Participants exhibiting signs of acute intoxication or psychosis were excluded for ethical and methodological reasons, but these exclusions may have systematically removed individuals experiencing the most severe forms of psychiatric distress or marginalization. As such, the mental health burden in the broader sex worker population might be underestimated.

Standardized, validated instruments were used. However, no data were available to assess the validity among sex workers. Several important variables remain unassessed, such as ethnicity (beyond citizenship and migration background), sexual orientation, or health insurance status, which could all significantly affect the individual experience of being a sex worker. Data were collected through self-report interviews, which are inherently subject to biases such as social desirability, recall bias, and underreporting, particularly concerning sensitive topics like mental illness, substance use, coercion, or experiences of violence. Although anonymity was ensured, and interviewers were trained to conduct the interviews, stigma and fear of legal consequences may have led to misreporting. Similarly, interviewer bias must be taken into consideration, despite training scientific staff. Although the study made significant efforts to offer linguistically diverse and culturally sensitive materials, subtle nuances or culturally specific understandings may have been lost in translation. We were not able to provide interpreters for all languages, although we covered 10 different interview languages, accommodating the most common nationalities in this industry (see Section 2). While the assumptions underlying the statistical analysis are based on a thorough literature review, it should be mentioned that there are no established models that explain the formation of QoL or mental diseases among sex workers. There are also no models on what factors influence workplace choice. This study would benefit from solid and validated models explaining the named outcomes.

## Data Availability

The datasets presented in this article are not readily available because of the sensitive nature of the data, datasets will be made available on request to other researchers. Requests to access the datasets should be directed to: olivia.kalinowski@charite.de.
