# Supplementary material for: Quality of life, mental health, and socio-demographic differences across sex work settings: implications for specialized healthcare and support services
Source: Front Public Health. 2025 Dec 4;13:1703735. doi: 10.3389/fpubh.2025.1703735 (PMC12711543; doi:10.3389/fpubh.2025.1703735)
Supplement: Supplementary file 1 [file Supplementary_file_1.pdf]

List of Variable Measures

1. Gender	What is your gender?	❒ Female<br>❒ Male<br>❒ Diverse
2. Age	In which year were you born?	
3. Citizenship	What nationality(ies) do you have?	
4. Migration Background	Was at least one of your parents not born in Germany?	❒ yes<br>❒ no
5. Languages	Which languages do you speak?	
6. Reading and Writing Literacy	Can you read and write?	❒ yes<br>❒ no
7. Children	Do you have children?	❒ yes<br>❒ no
8. Number of Children	*If yes, how many children do you have?	❒ 1<br>❒ 2<br>❒ 3<br>❒ more than 3
9. Homelessness	Are you or have you ever been homeless?	❒ yes<br>❒ no
10. Education	What is your highest educational qualification?	❒ No qualification<br>❒ I'm still a student<br>❒ Lower Secondary School Certificate<br>❒ Intermediate Secondary School Leaving Certificate<br>❒ Completed apprenticeship<br>❒ Entrance qualification for universities of applied science ("Fachabitur", "Fachhochschulreife")<br>❒ university entrance qualification, high school degree ("Abitur")<br>❒ university degree<br>❒ Other degree
11. Age of Beginning	How old were you when you started working in sex work?	____ Years
12. Involvement of third parties	Is there someone involved in your activity / protecting you / controlling you or similar?	❒ yes<br>❒ no

13.	*If yes, how would you describe this person?	
14. Relationship	Do you live in a (steady) relationship?	❒ yes<br>❒ no
15.	*If yes, does your partner know about your activity in the context of sex work?	❒ yes<br>❒ no
16. Income	Approximately what is your monthly income?	❒ I have no income of my own<br>❒ under 500 €<br>❒ 500 - 1000 €<br>❒ 1000€ - 3000 €<br>❒ 3000 € or more
17.	Is your activity in the sex work environment your main source of income?	❒ yes<br>❒ no
18. Number of clients	How many customers/clients do you have/serve on average?	❒ <5/day<br>❒ >5/day<br>❒ <5/week<br>❒ >5/week<br>❒ <5/month<br>❒ >5/month
19. Working Days	How many days do you usually work per week?	_____ days per week
20. Setting	Where do you usually meet/ look after your customers/ clients?<br>*(multiple answers possible)*	❒ In my own apartment<br>❒ In an apartment/studio rented for the activity<br>❒ In a brothel<br>❒ In a (massage) salon<br>❒ In a club/ cabaret club<br>❒ At the customer's/client's home<br>❒ In a car (your own or the customer's/client's)<br>❒ In a hotel<br>❒ On the street/ outdoors<br>❒ In a caravan/ camper<br>❒ online<br>❒ in different places (e.g. shopping, restaurant, travel) / escort<br>❒ girlfriend experience
	*If you work elsewhere, please tell us where:	

21. Other Sex Workers	Are there other sex workers where you work?	❒ yes<br>❒ no
22. Social Network	Do you have social contacts outside the context of sex work?	❒ yes<br>❒ no
23.	*If yes, do they know about your work?	❒ yes<br>❒ no
24. Sexual Services	What sexual services/practices do you offer?<br><br>*(multiple answers possible)*	❒ vaginal penetration<br>❒ anal penetration<br>❒ oral sex<br>❒ others<br>❒ Not applicable
25. Excluded services	Are there certain practices/things in your job that you exclude/dislike?	❒ yes<br>❒ no
26. Rule-breaking	Do you experience that customers/clients do not follow your rules?	❒ yes<br>❒ no
27. Specialization	Do you have a specialization and if yes, which one?<br>*(Multiple answers possible)*	❒ none<br>❒ dominatrix<br>❒ fetish<br>❒ BDSM<br>❒ tantra<br>❒ other
28. Reasons for Sex Work	Are you involved in sex work because...<br>*(multiple answers possible)*	❒ you like it<br>❒ it is well paid<br>❒ you support your family<br>❒ you support your partner<br>❒ you pay off debt<br>❒ you fund drugs<br>❒ you finance your training/study<br>❒ you have no choice<br>❒ you are forced to do so by someone<br>❒ you are forced to do so by the circumstances (e.g. no alternative to earning well enough with other jobs).<br>❒ other reasons
29. Positive Aspects of Sex Work	What do you like about your work in the sex work environment?<br>*(multiple answers possible)*	❒ power/ dominance<br>❒ sexual satisfaction<br>❒ the feeling of doing something good or of helping someone<br>❒ independence

		❒ freedom of choice (working hours, atmosphere, etc.)<br>❒ self-confidence, self-respect<br>❒ recognition and support from friends (common interests, experiences, protection, information flow, communication, social support)<br>❒ money<br>❒ attraction of the scene/community/ network<br>❒ other
30. Exit Wish	Do you want to leave sex work?	❒ yes<br>❒ no
31.	*If yes, how many times have you tried to leave?	❒ never<br>❒ 1x<br>❒ 2-3x<br>❒ more than 3x
32. Burdens	*To what extent do you feel burdened by the following points?*	
	1. The activity itself	❒ never true<br>❒ a little<br>❒ a lot<br>❒ very much
	2. The risk of contracting an STD	❒ never true<br>❒ a little<br>❒ a lot<br>❒ very much
	3. Stress caused by external working conditions/working conditions (e.g. weather, poor hygienic conditions, standing for long periods, long waiting times, working hours, competition)	❒ never true<br>❒ a little<br>❒ a lot<br>❒ very much
	4. financial exploitation	❒ never true<br>❒ a little<br>❒ a lot<br>❒ very much
	5. Being forced to work	❒ never true<br>❒ a little<br>❒ a lot<br>❒ very much

	6. Violence (mental, physical or sexual violence)	❒ never true<br>❒ a little<br>❒ a lot<br>❒ very much
	7. Special demands/requests by customers/clients	❒ never true<br>❒ a little<br>❒ a lot<br>❒ very much
	8. Double life (discrimination/ isolation)	❒ never true<br>❒ a little<br>❒ a lot<br>❒ very much
	9. Relationship difficulties	❒ never true<br>❒ a little<br>❒ a lot<br>❒ very much
	10. Financial dependency, difficult financial situation	❒ never true<br>❒ a little<br>❒ a lot<br>❒ very much
	11. Fear of being arrested	❒ never true<br>❒ a little<br>❒ a lot<br>❒ very much
	12. Sexual problems (e.g. no desire, disgust)	❒ never true<br>❒ a little<br>❒ a lot<br>❒ very much
	13. Feelings of guilt, shame, helplessness	❒ never true<br>❒ a little<br>❒ a lot<br>❒ very much
	14. Health problems (e.g., abdominal pain, infections)	❒ never true<br>❒ a little<br>❒ a lot<br>❒ very much
	15. Please list other difficulties, if applicable:	
33. Needs	What do you need?<br>*(Please tick anything you think is appropriate for your situation)*	
34.	A home/ safe place	❒
35.	Protection from physical attacks	❒
36.	Assistance in exiting the business	❒
37.	Therapy to quit drugs and alcohol	❒
38.	Medical support	❒
39.	Attorney/ legal support	❒

40.	Residence permit/ legal status	❐
41.	Better and safer working conditions	❐
42.	Recognition of sex work as a normal occupation/work activity	❐
43.	Another job/(another) education	❐
44.	Professional interpreters	❐
45.	I don’t need anything	❐
46.	other:	
