# Supplementary material for: Quality of life, mental health, and socio-demographic differences across sex work settings: implications for specialized healthcare and support services
Source: Front Public Health. 2025 Dec 4;13:1703735. doi: 10.3389/fpubh.2025.1703735 (PMC12711543; doi:10.3389/fpubh.2025.1703735)
Supplement: Supplementary file 2 [file Supplementary_file_2.pdf]

Statistical Protocol

STATISTICAL PROTOCOL **1**

1. RESEARCH QUESTIONS, INCLUDED VARIABLES AND STATISTICAL PROCEDURES 2
   1.1. How is sex work setting associated with quality of life, mental health, and working conditions among sex workers? *2*
   1.2. Which factors are associated with engagement in specific sex work settings? *3*
   1.3. How are reported healthcare and support needs associated with sex work setting? *4*
2. MODEL ASSUMPTIONS, STABILITY AND BONFERRONI CORRECTION 4
   2.1. Ordinary Least Squares (OLS) Models *4*
   2.2. Binary Logistic Regression Models *4*
   2.3. Ordinal and Partial Proportional Odds (PPO) Models *5*
   2.4. General Stability and Sensitivity Analyses *5*
   2.5. Bonferroni-Correction *5*
3. EVALUATION OF MODEL ASSUMPTIONS AND ROBUSTNESS 6
   3.1. How is sex work setting associated with quality of life, mental health, and working conditions among sex workers? *6*
      3.1.1. Is sex work setting a predictor of quality of life? 6
         a) Physical Component 6
         b) Mental Component 9
      3.1.2. Is sex work setting a predictor of mental diseases? 12
      3.1.3. Is sex work setting a predictor of working conditions? 16
         a) Does sex work setting predict perceived burdens? 16
         b) Does sex work setting predict rule-breaking by clients? 20
         c) Does sex work setting predict specialization? 20
         d) Does setting predict the desire to exit sex work? 24
         e) Does setting predict reported positive aspects? 24
         f) Does Setting predict Monthly Income? 25
   3.2. Which factors are associated with engagement in specific sex work settings? *26*
      3.2.1. Do socio-economic factors predict work setting? 26
      3.2.2. Do mental diseases predict specific work settings? 31
      3.2.3. Do self-reported reasons predict the work setting? 33
   3.3. How are reported healthcare and support needs associated with sex work setting? *38*

1. Research Questions, Included Variables and Statistical Procedures

1.1. How is sex work setting associated with quality of life, mental health, and working conditions among sex workers?

1.1.1. Is sex work setting a predictor of quality of life?

Independent Variables	Dependent Variable	Statistical Method
Street/Car<br>Hotel/Client´s apartment<br>Escort<br>Brothel<br>Studio<br>Own apartment<br>Online<br>Club	Quality of Life Mental component<br><br>Quality of Life Physical component	Ordinary least squares linear regression, when OLS model assumptions were violated, we used robust linear models (RLMs)

1.1.2. Is sex work setting a predictor of mental diseases?

Independent Variables	Dependent Variables	Statistical Method
Street/Car<br>Hotel/Client´s apartment<br>Escort<br>Brothel<br>Studio<br>Own apartment<br>Online	Anxiety Disorders<br>Affective Disorders<br>Addiction<br>OCD<br>Eating Disorders<br>PTSD<br>Somatization<br>Sleeping Disorders	Binary logistic regression

1.1.3. Is sex work setting a predictor of working conditions?

Independent Variables	Dependent Variables	Statistical Method
Street/Car<br>Hotel/Client´s apartment<br>Escort<br>Brothel	Perceived burden<br>Income	Cumulative link (ordinal) regression, when the parallel assumption was violated, we fitted

Studio<br>Own apartment<br>Online		partial proportional odds (PPO) models
	Desire to exit<br>Rule breaking by clients<br>Sex work specialization<br>Positive aspects	Binary logistic regression

1.2. Which factors are associated with engagement in specific sex work settings?

1.2.1. Do socio-demographic factors predict specific work settings?

Independent Variables	Dependent Variables	Statistical Method
Citizenship<br>Migration background<br>Relationship status<br>Children<br>Social Contact outside of sex work<br>Involvement of third parties<br>Age at starting sex work<br>Educational background<br>Homelessness	Street/Car<br>Hotel/Client´s apartment<br>Escort<br>Brothel<br>Studio<br>Own apartment<br>Online	Binary logistic regression

1.2.2. Do mental diseases predict specific work settings?

Independent Variables	Dependent Variables	Statistical Method
Anxiety Disorders<br>Affective Disorders<br>Addiction<br>OCD<br>Eating Disorders<br>PTSD<br>Somatization<br>Sleeping Disorders	Street/Car<br>Hotel/Client´s apartment<br>Escort<br>Brothel<br>Studio<br>Own apartment<br>Online	Binary logistic regression

1.2.3. Do self-reported reasons for sex work predict specific work settings?

Independent Variables	Dependent Variables	Statistical Method

Reasons for sex work	Street/Car<br>Hotel/Client´s apartment<br>Escort<br>Brothel<br>Studio<br>Own apartment<br>Online	Binary logistic regression

1.3. How are reported healthcare and support needs associated with sex work setting?

1.3.1. Does work setting predict the reported needs?

Independent Variables	Dependent Variables	Statistical Method
Street/Car<br>Hotel/Client´s apartment<br>Escort<br>Brothel<br>Studio<br>Own apartment<br>Online	Reported socio-medical needs	Binary logistic regression

2. Model Assumptions, Stability and Bonferroni Correction

To ensure the validity and robustness of our statistical analyses, model assumptions were systematically evaluated for each model type.

2.1. Ordinary Least Squares (OLS) Models

For continuous outcomes (SF-12 Mental and Physical Component Scores), residuals were visually inspected using Q-Q plots and histograms to assess normality, and residuals versus fitted plots were used to evaluate homoscedasticity. The Breusch–Pagan test was applied to formally test for heteroscedasticity. Normality was additionally assessed using the Shapiro–Wilk test, acknowledging that minor deviations are expected in large samples and unlikely to bias estimates. Multicollinearity among predictors was evaluated using variance inflation factors (VIFs), and influential observations were identified using Cook's distance. Where the assumptions were violated, we used robust linear models (RLMs) to calculate effect sizes and confidence intervals.

2.2. Binary Logistic Regression Models

For binary outcomes, model adequacy was assessed using the Hosmer–Lemeshow goodness-of-fit test, and calibration plots were inspected where appropriate. The

linearity of continuous predictors in the logit was evaluated to confirm appropriate model specification, where appropriate (however, most predictors were binary). Multicollinearity among predictors was checked using VIFs. To assess model stability, we examined standard errors, confidence intervals, and changes in estimates when potential outliers or sparse cells were excluded in sensitivity analyses. Model fit was assessed using McFadden's pseudo-R^2.

2.3. Ordinal and Partial Proportional Odds (PPO) Models

For ordered categorical outcomes (perceived burden, income), cumulative link (ordinal) regression models were fitted. The proportional odds (parallel regression) assumption was tested using the Brant test. Where the assumption was violated, PPO models were fitted, allowing category-specific (non-parallel) effects for those predictors. Model fit was assessed using McFadden's pseudo-R^2, observed-versus-predicted probabilities, and calibration plots where appropriate. Multicollinearity was evaluated with VIFs.

2.4. General Stability and Sensitivity Analyses

For all regression models, we inspected confidence intervals, standard errors, and convergence criteria to ensure numerical stability. Where categories had very small sample sizes, we examined whether collapsing categories affected estimates. Missing data were handled using listwise deletion after confirming that excluded cases did not differ systematically from the analytic sample in key covariates.

2.5. Bonferroni-Correction

The significance threshold for each predictor was adjusted using the Bonferroni correction per outcome to account for the increased risk of Type I error due to multiple testing. The original significance level of $\alpha = 0.05$ was divided by the number of tests per outcome. The p-value for QoL analysis is therefore $p < 0.025$. P-values < 0.006 were considered statistically significant within each diagnostic group for the question of whether work setting predicts mental disorders and vice versa. For the burden variables, 0.003 was set as the p-value threshold. For positive aspects of sex work, it was 0.005. P-value for the model with sociodemographic variables as predictors of setting was set at 0.002, while the significance threshold for reasons predicting setting was set at $p < 0.005$. The p-value for the needs analysis was set to 0.004.

3. Evaluation of Model Assumptions and Robustness

3.1. How is sex work setting associated with quality of life, mental health, and working conditions among sex workers?

3.1.1. Is sex work setting a predictor of quality of life?

a) Physical Component

Linearity: Residuals vs Fitted

Normality of residuals:

Shapiro-Wilk normality test

W = 0.92794, p-value = 8.012e-13

Homoscedasticity: Breusch-Pagan Test
BP = 8.6794, df = 8, p-value = 0.3701

Multicollinearity: VIF

Location	Value
Street/Car	1.126800
Escort (diverse)	1.147137
Hotel/Client's apartment	1.226044
Online	1.101742

Club	1.017161
Brothel	1.048349
Studio	1.033966
Own apartment	1.038446

Influential Points: Cook's Distance

Model results after removing Outliers (no significant changes):

Predictor	Estimate	Std. Error	t value	p-value	Significance
(Intercept)	37.31	6.43	5.81	< 0.001	***
Car_Street	3.19	1.08	2.95	0.003	**
Diverse_Escort	-1.75	1.13	-1.55	0.122	n.s.
Client_Hotel	1.88	1.02	1.84	0.067	.
online	0.80	1.05	0.77	0.445	n.s.
club	1.21	1.63	0.74	0.459	n.s.
brothel	0.64	1.47	0.43	0.665	n.s.

studio	0.39	1.03	0.38	0.702	n.s.
own_apartment	0.99	1.46	0.68	0.498	n.s.

**Model Fit Statistics**	**Value**
Residual standard error	8.86 (df = 364)
Multiple R^2	0.048
Adjusted R^2	0.027
F-statistic	2.27 (df = 8, 364), p = 0.022
N (after deletion)	372

b) Mental Component

Linearity: Residuals vs Fitted

Normality of residuals: Histogram of Residuals

Shapiro-Wilk normality test

W = 0.97659, p-value = 5.612e-06

Homoscedasticity: Breusch-Pagan Test
BP = 9.7274, df = 8, p-value = 0.2847

Multicollinearity (VIF):

Location	Value
Street/Car	1.126800
Escort (diverse)	1.147137
Hotel/Client's apartment	1.226044
Online	1.101742
Club	1.017161
Brothel	1.048349
Studio	1.033966
Own apartment	1.038446

Influential points: Cook´s Distance

Model results after removing Outliers (no significant changes):

Predictor	Estimate	Std. Error	t value	p-value	Significance
(Intercept)	22.00	8.37	2.63	0.009	**
Car_Street	3.34	1.37	2.43	0.016	*
Diverse_Escort	-0.57	1.42	-0.41	0.686	n.s.

Client_Hotel	3.38	1.29	2.61	0.009	**
online	0.34	1.32	0.26	0.794	n.s.
club	7.74	2.14	3.61	< 0.001	***
brothel	-2.21	1.88	-1.18	0.240	n.s.
studio	-3.07	1.31	-2.35	0.019	*
own_apartment	2.73	1.84	1.49	0.138	n.s.

**Model Fit Statistics**	**Value**
Residual standard error	11.14 (df = 360)
Multiple R^2	0.107
Adjusted R^2	0.087
F-statistic	5.41 (df = 8, 360), $p < 0.001$
N (after deletion)	368

3.1.2. Is sex work setting a predictor of mental diseases?

**Diagnostic Test**	**Result**
**Model type**	Binary logistic regression
**Outcome variable**	Anxiety disorder (yes/no)
**McFadden's Pseudo-R^2**	0.068
**Variance Inflation Factors (VIFs)**	
Street/Car	1.12
Escort (diverse)	1.16
Hotel/Client's apartment	1.29
Online	1.15
Club	1.02
Brothel	1.04
Studio	1.04
Own apartment	1.04
**Hosmer–Lemeshow Goodness-of-Fit Test**	
χ^2 (df = 8)	5.48
p-value	0.706
**Model fit interpretation**	Non-significant *p* indicates good model fit. Multicollinearity was low (all VIFs < 1.3).

**Diagnostic Test**	**Result**
**Model type**	Binary logistic regression
**Outcome variable**	Affective disorder (yes/no)
**McFadden's Pseudo-R^2**	0.053
**Variance Inflation Factors (VIFs)**	
Street/Car	1.11
Escort (diverse)	1.18

Hotel/Client's apartment	1.25
Online	1.14
Club	1.03
Brothel	1.03
Studio	1.04
Own apartment	1.04
**Hosmer–Lemeshow Goodness-of-Fit Test**	
χ^2 (df = 7)	9.05
p-value	0.249
**Model fit interpretation**	Non-significant *p* indicates good model fit. Multicollinearity was low (all VIFs < 1.3).

Diagnostic Test	Result
**Model type**	Binary logistic regression
**Outcome variable**	Obsessive-Compulsive Disorder (yes/no)
**McFadden's Pseudo-R²**	0.071
**Variance Inflation Factors (VIFs)**	
Street/Car	1.09
Escort (diverse)	1.18
Hotel/Client's apartment	1.18
Online	1.12
Club	1.05
Brothel	1.04
Studio	1.03
Own apartment	1.09
**Hosmer–Lemeshow Goodness-of-Fit Test**	
χ^2 (df = 8)	2.47
p-value	0.963
**Model fit interpretation**	Non-significant *p* indicates good model fit. Multicollinearity was low (all VIFs < 1.2).

Diagnostic Test	Result
**Model type**	Binary logistic regression
**Outcome variable**	Eating disorder (yes/no)
**McFadden's Pseudo-R²**	0.074
**Variance Inflation Factors (VIFs)**	
Street/Car	1.07
Escort (diverse)	1.20
Hotel/Client's apartment	1.18
Online	1.17
Club	1.03
Brothel	1.05

Studio	1.03
Own apartment	1.12
**Hosmer–Lemeshow Goodness-of-Fit Test**	
χ^2 (df = 8)	1.71
p-value	0.636
**Model fit interpretation**	Non-significant *p* indicates good model fit. Multicollinearity was low (all VIFs < 1.2).

**Diagnostic Test**	**Result**
**Model type**	Binary logistic regression
**Outcome variable**	Somatization (yes/no)
**McFadden's Pseudo-R²**	0.017
**Variance Inflation Factors (VIFs)**	
Street/Car	1.11
Escort (diverse)	1.18
Hotel/Client's apartment	1.20
Online	1.13
Club	1.02
Brothel	1.03
Studio	1.04
Own apartment	1.07
**Hosmer–Lemeshow Goodness-of-Fit Test**	
χ^2 (df = 8)	3.92
p-value	0.864
**Model fit interpretation**	Non-significant *p* indicates good model fit. Multicollinearity was low (all VIFs < 1.21).

**Diagnostic Test**	**Result**
**Model type**	Binary logistic regression
**Outcome variable**	Addiction (yes/no)
**McFadden's Pseudo-R²**	0.151
**Variance Inflation Factors (VIFs)**	
Street/Car	1.08
Escort (diverse)	1.16
Hotel/Client's apartment	1.12
Online	1.14
Club	1.06
Brothel	1.03
Studio	1.06
Own apartment	1.05
**Hosmer–Lemeshow Goodness-of-Fit Test**	
χ^2 (df = 8)	12.75

p-value	0.121
**Model fit interpretation**	Non-significant *p* indicates good model fit. Multicollinearity was low (all VIFs < 1.2).

**Diagnostic Test**	**Result**
**Model type**	Binary logistic regression
**Outcome variable**	Sleep disorder (yes/no)
**McFadden's Pseudo-R²**	0.033
**Variance Inflation Factors (VIFs)**	
Street/Car	1.11
Escort (diverse)	1.17
Hotel/Client's apartment	1.24
Online	1.12
Club	1.02
Brothel	1.03
Studio	1.03
Own apartment	1.05
**Hosmer–Lemeshow Goodness-of-Fit Test**	
χ^2 (df = 8)	7.64
p-value	0.470
**Model fit interpretation**	Non-significant *p* indicates good model fit. Multicollinearity was low (all VIFs < 1.24).

**Diagnostic Test**	**Result**
**Model type**	Binary logistic regression
**Outcome variable**	PTSD (yes/no)
**McFadden's Pseudo-R²**	0.047
**Variance Inflation Factors (VIFs)**	
Street/Car	1.10
Escort (diverse)	1.17
Hotel/Client's apartment	1.20
Online	1.11
Club	1.03
Brothel	1.05
Studio	1.04
Own apartment	1.06
**Hosmer–Lemeshow Goodness-of-Fit Test**	
χ^2 (df = 7)	5.25
p-value	0.630
**Model fit interpretation**	Non-significant *p* indicates good model fit. Multicollinearity was low (all VIFs < 1.21).

3.1.3. Is sex work setting a predictor of working conditions?

a) Does sex work setting predict perceived burdens?

Brant Test Results for each Model:

Model (Outcome)	Omnibus χ^2	df	p-value	Interpretation
**Work-related strain / burden**	**30.5**	**16**	**0.02**	**Violation (p < .05)**
**Burden due to sexually transmitted diseases (STDs)**	**46.9**	**16**	**<0.001**	**Violation (p < .001)**
Burden due to working conditions / circumstances	16.5	16	0.42	Assumption met
**Burden due to financial exploitation**	**29.7**	**16**	**0.02**	**Violation (p < .05)**
Burden due to coercion	9.44	16	0.89	Assumption met
Burden due to violence	10.96	16	0.81	Assumption met
**Burden due to client demands**	**46.2**	**16**	**<0.001**	**Violation (p < .001)**
Burden due to leading a double life	19.7	16	0.23	Assumption met
Burden due to relationship problems	10.73	16	0.83	Assumption met
Burden due to financial dependency	18.66	16	0.29	Assumption met
Burden due to risk or experience of arrest	17.92	16	0.33	Assumption met
Burden due to sexual difficulties	24.87	16	0.07	Assumption met
Burden due to guilt and shame	16.2	16	0.44	Assumption met
Burden due to health problems	11.25	16	0.79	Assumption met

**Variance Inflation Factors (VIFs)**		
**Outcome**	**VIF range**	**Interpretation**
Work-related strain / burden	1.02 – 1.25	No multicollinearity
Burden due to sexually transmitted diseases (STDs)	1.02 – 1.18	No multicollinearity
Burden due to working conditions / circumstances	1.02 – 1.17	No multicollinearity
Burden due to financial exploitation	1.04 – 1.24	No multicollinearity
Burden due to coercion	1.05 – 1.18	No multicollinearity

Burden due to violence	1.03 – 1.22	No multicollinearity
Burden due to client demands	1.02 – 1.23	No multicollinearity
Burden due to leading a double life	1.02 – 1.24	No multicollinearity
Burden due to relationship problems	1.02 – 1.20	No multicollinearity
Burden due to financial dependency	1.02 – 1.19	No multicollinearity
Burden due to risk or experience of arrest	1.03 – 1.17	No multicollinearity
Burden due to sexual difficulties	1.02 – 1.21	No multicollinearity
Burden due to guilt and shame	1.02 – 1.20	No multicollinearity
Burden due to health problems	1.03 – 1.18	No multicollinearity

McFadden Pseudo R^2:

**Outcome**	**McFadden Pseudo R^2**
Work-related strain / burden	0.06061837
Burden due to sexually transmitted diseases (STDs)	0.05525449
Burden due to working conditions / circumstances	0.09871806
Burden due to financial exploitation	0.06268377
Burden due to coercion	0.04955484
Burden due to violence	0.05510608
Burden due to client demands	0.01495492
Burden due to leading a double life	0.01267938
Burden due to relationship problems	0.0380485
Burden due to financial dependency	0.06556648
Burden due to risk or experience of arrest	0.05991072
Burden due to sexual difficulties	0.03033213
Burden due to guilt and shame	0.04612155
Burden due to health problems	0.06985672

A partial proportional odds (PPO) ordinal logistic model was used for outcomes that did not satisfy the proportional odds assumption:

Work-related strain / burden:

**Predictor**	**Estimate**	**Std. Error**	**z value**	**p-value**	**OR**	**Significance**
(Intercept):1	2.2164	1.4025	1.580	0.1140	–	
(Intercept):2	0.9469	1.3992	0.677	0.4986	–	
(Intercept):3	-0.1775	1.4050	-0.126	0.8994	–	
**Car_Street**	**-1.0307**	0.2315	-4.452	**<0.001**	**0.36**	***
**Diverse_Escort**	0.4193	0.2608	1.607	0.1080	1.52	
**Client_Hotel**	**-0.7590**	0.2391	-3.174	**0.002**	**0.47**	**
**online**	0.4256	0.2531	1.681	0.0927	1.53	.

**club**	-0.5643	0.3645	-1.548	0.1216	0.57	
**brothel**	-0.4059	0.3201	-1.268	0.2047	0.67	
**studio**	0.4707	0.2468	1.907	0.0565	1.60	.
**own_apartment**	-0.1778	0.3239	-0.549	0.5831	0.84	
**Residual deviance: 763.9974 (df = 1183)**<br>**Log-likelihood: –381.9987**<br>**Iterations: 5 (no Hauck–Donner effect detected)**<br>**Link function: logit**<br>**Outcome: Work-related strain**						

Burden due to financial exploitation:

**Predictor**	**Estimate**	**Std. Error**	**z value**	**p-value**	**OR**	**Signif.**
(Intercept)	2.7092	1.3920	1.946	0.0516	—	—
Diverse_Escort	0.7378	0.2690	2.743	0.0061	2.091	**
Client_Hotel	-0.5750	0.2350	-2.447	0.0144	0.563	*
online	-0.2110	0.2404	-0.878	0.3800	0.810	
club	-0.7077	0.3612	-1.959	0.0501	0.493	
brothel	-0.4749	0.3204	-1.482	0.1383	0.622	
studio	0.2299	0.2398	0.959	0.3377	1.259	
Car_Street:1	-1.2612	0.2425	-5.201	<0.001	0.283	***
Car_Street:2	-1.1090	0.2629	-4.219	<0.001	0.330	***
Car_Street:3	-1.2570	0.3525	-3.566	<0.001	0.285	***
own_apartment:1	0.3804	0.3474	1.095	0.2736	1.463	
own_apartment:2	-0.3836	0.3634	-1.056	0.2911	0.681	
own_apartment:3	-0.8668	0.4078	-2.126	0.0335	0.420	**
**Residual deviance 782.07 (df = 1178)**<br>**Log-likelihood -391.03 (df = 1178)**<br>**Fisher scoring iterations 5**<br>**Hauck–Donner effect None detected**						

Burden due to sexually transmitted diseases (not stable):

**Predictor**	**Estimate**	**Std. Error**	**z value**	**p-value**	**Signif.**	**OR**
(Intercept)	2.851	0.4796	5.945	2.77e-09	***	—
Car_Street	-0.6128	1.069e-06	-5.731e+05	< 2e-16	***	0.542
Diverse_Escort:1	-0.2855	0.2092	-1.365	0.172		0.752
Diverse_Escort:2	0.4694	0.2106	NA	NA		1.599
Diverse_Escort:3	0.2335	0.2863	0.816	0.415		1.263
Client_Hotel	-0.7139	1.507e-06	-4.737e+05	< 2e-16	***	0.489

online	0.6392	1.309e-06	4.884e+05	< 2e-16	***	1.895
club:1	0.0472	0.1205	NA	NA		1.048
club:2	-1.244	0.1266	-9.826	< 2e-16	***	0.288
club:3	-1.542	0.2365	-6.521	6.96e-11	***	0.214
brothel	-0.5611	1.546e-06	-3.630e+05	< 2e-16	***	0.571
studio	0.1293	1.541e-06	8.386e+04	< 2e-16	***	1.138
own_apartment	-0.1919	2.083e-06	-9.210e+04	< 2e-16	***	0.826
**Model Fit Statistics**						
Residual deviance			4464.63 (df = 1181)			
Log-likelihood			NA (Hauck–Donner effect detected)			
Fisher scoring iterations			2			
Hauck–Donner effect			Detected for: (Intercept), Diverse_Escort:2, Client_Hotel, online, club:1, club:3, brothel			

Warning: some probabilities are very close to 0

Burden due to client demands:

**Predictor**	**Estimate**	**Std. Error**	**z value**	**p-value**	**Significance**	**OR**
(Intercept)	1.85581	1.31510	1.411	0.1582		—
Car_Street:1	0.01391	0.23512	0.059	0.9528		1.014
Car_Street:2	-1.00786	0.26065	-3.867	0.00011	***	0.365
Car_Street:3	-1.36921	0.36277	-3.774	0.00016	***	0.254
Diverse_Escort	0.12368	0.23219	0.533	0.5943		1.132
Client_Hotel	-0.49591	0.21572	-2.299	0.02151	*	0.609
online:1	-0.79065	0.22317	-3.543	0.000396	***	0.453
online:2	0.19496	0.22536	0.865	0.387		1.215
online:3	0.14648	0.38285	0.383	0.702		1.158
club	-0.03168	0.34879	-0.091	0.9276		0.969
brothel	-0.24430	0.30119	-0.811	0.4173		0.783
studio	0.05660	0.21298	0.266	0.7904		1.058
own_apartment:1	0.39254	0.31093	1.262	0.2068		1.481
own_apartment:2	-0.55692	0.32366	NA	NA		0.573
own_apartment:3	-0.85464	0.43700	-1.956	0.0505	.	0.425
**Residual deviance: 895.20 (df = 1176)**<br>**Log-likelihood: -447.60 (df = 1176)**<br>**Iterations: 12**<br>**Hauck–Donner effect detected for own_apartment:2**						

b) Does sex work setting predict rule-breaking by clients?

**Diagnostic Test**	**Result**
**Model type**	Binary logistic regression
**Outcome variable**	Rule breaking by clients (yes/no)
**McFadden's Pseudo-R^2**	0.100
**Cox & Snell R^2**	0.128
**Nagelkerke R^2**	0.134
**Brier Score**	0.213
**Variance Inflation Factors (VIFs) for predictors (Work Settings)**	
Street/Car	1.077
Escort (diverse)	1.159
Hotel/Client's apartment	1.219
Online	1.081
Club	1.014
Brothel	1.054
Studio	1.034
Own apartment	1.045
**Hosmer–Lemeshow Goodness-of-Fit Test**	
χ^2 (df = 3)	3.954
p-value	0.267
**Model fit interpretation**	Non-significant *p* indicates acceptable model fit. Multicollinearity is low (all VIFs < 1.22). Brier score indicates moderate predictive accuracy.

c) Does sex work setting predict specialization?

**Diagnostic Test**	**Result**
**Model type**	Binary logistic regression
**Outcome variable**	No Specialization (yes/no)
**McFadden's Pseudo-R^2**	0.088
**Nagelkerke R^2**	0.154
**Brier Score**	0.22
**Variance Inflation Factors (VIFs) for predictors (Work Settings)**	
Street/Car	1.10
Escort (diverse)	1.15
Hotel/Client's apartment	1.25
Online	1.10
Club	1.02

Brothel	1.06
Studio	1.03
Own apartment	1.04
**Hosmer–Lemeshow Goodness-of-Fit Test**	
χ^2 (df = 8)	11.28
p-value	0.186
**Model fit interpretation**	Non-significant *p* indicates acceptable model fit. Multicollinearity is low (all VIFs < 1.25). Brier score suggests moderate predictive accuracy.

**Diagnostic Test**	**Result**
**Model type**	Binary logistic regression
**Outcome variable**	Dominatrix (yes/no)
**McFadden's Pseudo-R²**	0.104
**Nagelkerke R²**	0.161
**Brier Score**	0.156
**Variance Inflation Factors (VIFs) for predictors (Work Settings)**	
Street/Car	1.09
Escort (diverse)	1.15
Hotel/Client's apartment	1.21
Online	1.07
Club	1.01
Brothel	1.02
Studio	1.06
Own apartment	1.06
**Hosmer–Lemeshow Goodness-of-Fit Test**	
χ^2 (df = 7)	10.59
p-value	0.158
**Model fit interpretation**	Non-significant *p* indicates acceptable model fit. Multicollinearity is low (all VIFs < 1.25). Brier score indicates reasonable predictive accuracy.

**Diagnostic Test**	**Result**
**Model type**	Binary logistic regression
**Outcome variable**	Fetish (yes/no)
**McFadden's Pseudo-R²**	0.136
**Nagelkerke R²**	0.205
**Brier Score**	0.145
**Variance Inflation Factors (VIFs) for predictors (Work Settings)**	

Street/Car	1.07
Escort (diverse)	1.16
Hotel/Client's apartment	1.21
Online	1.08
Club	1.03
Brothel	1.03
Studio	1.04
Own apartment	1.07
**Hosmer–Lemeshow Goodness-of-Fit Test**	
χ^2 (df = 3)	6.58
p-value	0.086
**Model fit interpretation**	Non-significant *p* indicates acceptable model fit. Multicollinearity is low (all VIFs < 1.25). Brier score indicates reasonable predictive accuracy.

**Diagnostic Test**	**Result**
**Model type**	Binary logistic regression
**Outcome variable**	BDSM (yes/no)
**McFadden's Pseudo-R²**	0.223
**Nagelkerke R²**	0.312
**Brier Score**	0.117
**Variance Inflation Factors (VIFs) for predictors (Work Settings)**	
Street/Car	1.06
Escort (diverse)	1.14
Hotel/Client's apartment	1.20
Online	1.09
Club	1.03
Brothel	1.04
Studio	1.04
Own apartment	1.07
**Hosmer–Lemeshow Goodness-of-Fit Test**	
χ^2 (df = 8)	12.5
p-value	0.13
**Model fit interpretation**	Non-significant *p* indicates acceptable model fit. Multicollinearity is low (all VIFs < 1.25). Brier score indicates reasonable predictive accuracy.

**Diagnostic Test**	**Result**
**Model type**	Binary logistic regression

**Outcome variable**	Tantra (yes/no)
**McFadden's Pseudo-R²**	0.163
**Nagelkerke R²**	0.198
**Brier Score**	0.057
**Variance Inflation Factors (VIFs) for predictors (Work Settings)**	
Street/Car	1.04
Escort (diverse)	1.12
Hotel/Client's apartment	1.14
Online	1.07
Club	1.05
Brothel	1.00
Studio	1.07
Own apartment	1.06
**Hosmer–Lemeshow Goodness-of-Fit Test**	
χ^2 (df = 8)	4.83
p-value	0.776
**Model fit interpretation**	Non-significant *p* indicates excellent model fit. Multicollinearity is low (all VIFs < 1.25). Brier score indicates reasonable predictive accuracy.

**Diagnostic Test**	**Result**
**Model type**	Binary logistic regression
**Outcome variable**	Other (yes/no)
**McFadden's Pseudo-R²**	0.055
**Nagelkerke R²**	0.085
**Brier Score**	0.157
**Variance Inflation Factors (VIFs) for predictors (Work Settings)**	
Street/Car	1.07
Escort (diverse)	1.16
Hotel/Client's apartment	1.25
Online	1.11
Club	1.02
Brothel	1.07
Studio	1.03
Own apartment	1.04
**Hosmer–Lemeshow Goodness-of-Fit Test**	
χ^2 (df = 8)	5.4
p-value	0.714

**Model fit interpretation**	Non-significant *p* indicates good model fit. Multicollinearity is low (all VIFs < 1.3). Brier score indicates reasonable predictive accuracy.

d) Does setting predict the desire to exit sex work?

**Diagnostic Test**	**Result**
**Model type**	Binary logistic regression
**Outcome variable**	Desire to Exit (yes/no)
**McFadden's Pseudo-R^2**	0.155
**Nagelkerke R^2**	0.254
**Brier Score**	0.192
**Variance Inflation Factors (VIFs) for predictors (Work Settings)**	
Street/Car	1.08
Escort (diverse)	1.13
Hotel/Client's apartment	1.20
Online	1.06
Club	1.01
Brothel	1.06
Studio	1.03
Own apartment	1.04
**Hosmer–Lemeshow Goodness-of-Fit Test**	
χ^2 (df = 3)	1.12
p-value	0.771
**Model fit interpretation**	Non-significant *p* indicates good model fit. Multicollinearity is low (all VIFs < 1.3). Brier score indicates reasonable predictive accuracy.

e) Does setting predict reported positive aspects?

Positive aspect	McFadden: R^2	**Hosmer–Lemeshow Goodness-of-Fit Test** (p-value)	Mean VIF
power/ dominance	0.129	0.317	1.09
sexual satisfaction	0.0662	0.212	1.09
the feeling of doing something good or of helping someone	0.106	0.854	1.09

independence	0.159	0.614	1.08
freedom of choice (working hours, atmosphere, etc.)	0.128	0.652	1.09
self-confidence, self-respect	0.129	0.724	1.09
recognition and support from friends (common interests, experiences,protection, information flow, communication, social support)	0.0577	0.920	1.10
money	0.0422	0.849	1.09
attraction of the scene/community/ network	0.0898	0.341	1.09
other	0.0486	0.619	1.09

f) Does Setting predict Monthly Income?

Predictor / Test	χ^2	df	p-value	Interpretation
Omnibus	46.94	24	0.0034	Overall model indicates some violation of parallel regression assumption.
Car/Street	4.53	3	0.209	Parallel regression assumption holds.
Diverse/Escort	21.91	3	0.000068	Parallel regression assumption violated.
Client/Hotel	5.08	3	0.166	Parallel regression assumption holds.
Online	0.55	3	0.909	Parallel regression assumption holds.
Club	2.07	3	0.558	Parallel regression assumption holds.
Brothel	0.88	3	0.830	Parallel regression assumption holds.
Studio	5.62	3	0.132	Parallel regression assumption holds.
Own_Apartment	2.02	3	0.568	Parallel regression assumption holds.

Income partial parallel model:

**Predictor**	**Threshold / Linear Predictor**	**Estimate**	**Std. Error**	**z value**	**p-value**	**Signifi-cance**
(Intercept)	–	-0.149	1.299	-0.114	0.909	–
Car_Street	–	-1.380	0.226	-6.101	1.06e-09	***
Diverse_Escort	1	-1.374	0.301	-4.571	4.86e-06	***
Diverse_Escort	2	0.009	0.234	0.039	0.969	–

Diverse_Escort	3	0.681	0.231	2.945	0.003	**
Diverse_Escort	4	1.941	0.252	7.704	1.31e-14	***
Client_Hotel	–	-0.472	0.214	-2.208	0.027	*
online	–	0.593	0.218	2.723	0.006	**
club	–	-0.558	0.334	-1.670	0.095	.
brothel	–	0.258	0.307	0.842	0.400	–
studio	–	0.239	0.212	1.128	0.259	–
own_apartment	–	0.376	0.303	1.243	0.214	–

**Statistic**	**Value**	**Comment**
McFadden Pseudo-R^2	0.066	Small explanatory power
Residual deviance	1017.1 (df = 1560)	–
Log-likelihood	-508.55	–
Hauck-Donner effect	none detected	–

3.2. Which factors are associated with engagement in specific sex work settings?

3.2.1. Do socio-economic factors predict work setting?

**Diagnostic Test**	**Result**
**Model type**	Binary logistic regression
**Outcome variable**	Working in Street/Car setting (yes/no)
**McFadden's Pseudo-R^2**	0.334
**Brier Score**	0.151
**Variance Inflation Factors (VIFs) for predictors (Socioeconomic Factors)**	
(None) German citizenship	1.09
Parents from Germany (migration status)	1.00
Residence status	1.07
Children	1.25
Homelessness	1.12
Educational level	1.33
Stable relationship	1.07
Social contacts outside sex work	1.16
Third party involvement	1.15
Age at starting sex work	1.12
**Hosmer–Lemeshow Goodness-of-Fit Test**	
χ^2 (df = 3)	3.17
p-value	0.367
**Model fit interpretation**	Non-significant *p* indicates good model fit. Multicollinearity was low (all VIFs < 1.34).

	Brier score indicates reasonable predictive accuracy.

Diagnostic Test	Result
**Model type**	Binary logistic regression
**Outcome variable**	Working in Escort setting (yes/no)
**McFadden's Pseudo-R²**	0.216
**Brier Score**	0.122
**Variance Inflation Factors (VIFs) for predictors (Socioeconomic Factors)**	
(None) German citizenship	1.29
Parents from Germany (migration status)	1.15
Residence status	1.22
Children	1.21
Homelessness	1.16
Educational level	1.57
Stable relationship	1.04
Social contacts outside sex work	1.26
Third party involvement	1.13
Age at starting sex work	1.28
**Hosmer–Lemeshow Goodness-of-Fit Test**	
χ^2 (df = 3)	2.46
p-value	0.483
**Model fit interpretation**	Non-significant *p* indicates good model fit. Multicollinearity was generally low (all VIFs < 1.57). Brier score indicates reasonable predictive accuracy.

Diagnostic Test	Result
**Model type**	Binary logistic regression
**Outcome variable**	Working in Client/Hotel setting (yes/no)
**McFadden's Pseudo-R²**	0.034
**Brier Score**	0.236
**Variance Inflation Factors (VIFs) for predictors (Socioeconomic Factors)**	
(None) German citizenship	1.22
Parents from Germany (migration status)	1.13
Residence status	1.13
Children	1.31
Homelessness	1.22
Educational level	1.49

Stable relationship	1.05
Social contacts outside sex work	1.41
Third party involvement	1.06
Age at starting sex work	1.10
**Hosmer–Lemeshow Goodness-of-Fit Test**	
χ^2 (df = 3)	4.15
p-value	0.246
**Model fit interpretation**	Non-significant *p* indicates good model fit. Multicollinearity was low (all VIFs < 1.49). Brier score indicates reasonable predictive accuracy.

**Diagnostic Test**	**Result**
**Model type**	Binary logistic regression
**Outcome variable**	Working in Online setting (yes/no)
**McFadden's Pseudo-R²**	0.154
**Brier Score**	0.129
**Variance Inflation Factors (VIFs) for predictors (Socioeconomic Factors)**	
(None) German citizenship	1.32
Parents from Germany (migration status)	1.24
Residence status	1.13
Children	1.17
Homelessness	1.18
Educational level	1.56
Stable relationship	1.05
Social contacts outside sex work	1.36
Third party involvement	1.12
Age at starting sex work	1.21
**Hosmer–Lemeshow Goodness-of-Fit Test**	
χ^2 (df = 3)	6.04
p-value	0.110
**Model fit interpretation**	Non-significant *p* indicates good model fit. Multicollinearity was low (all VIFs < 1.56). Brier score indicates reasonable predictive accuracy.

**Diagnostic Test**	**Result**
**Model type**	Binary logistic regression
**Outcome variable**	Working in Club setting (yes/no)
**McFadden's Pseudo-R²**	0.113

**Brier Score**	0.078
**Variance Inflation Factors (VIFs) for predictors (Socioeconomic Factors)**	
(None) German citizenship	1.27
Parents from Germany (migration status)	1.00
Residence status	1.24
Children	1.26
Homelessness	1.21
Educational level	1.35
Stable relationship	1.05
Social contacts outside sex work	1.39
Third party involvement	1.08
Age at starting sex work	1.21
**Hosmer–Lemeshow Goodness-of-Fit Test**	
χ^2 (df = 3)	7.24
p-value	0.065
**Model fit interpretation**	Non-significant *p* indicates adequate model fit (borderline). Multicollinearity was low (all VIFs < 1.39). Brier score indicates reasonable predictive accuracy.

**Diagnostic Test**	**Result**
**Model type**	Binary logistic regression
**Outcome variable**	Working in Brothel setting (yes/no)
**McFadden's Pseudo-R²**	0.045
**Brier Score**	0.113
**Variance Inflation Factors (VIFs) for predictors (Socioeconomic Factors)**	
(None) German citizenship	1.14
Parents from Germany (migration status)	1.00
Residence status	1.11
Children	1.32
Homelessness	1.19
Educational level	1.43
Stable relationship	1.04
Social contacts outside sex work	1.32
Third party involvement	1.05
Age at starting sex work	1.19
**Hosmer–Lemeshow Goodness-of-Fit Test**	
χ^2 (df = 3)	0.50
p-value	0.919

**Model fit interpretation**	Non-significant *p* indicates excellent model fit. Multicollinearity was low (all VIFs < 1.43). Brier score indicates reasonable predictive accuracy.

**Diagnostic Test**	**Result**
**Model type**	Binary logistic regression
**Outcome variable**	Working in Studio setting (yes/no)
**McFadden's Pseudo-R²**	0.152
**Brier Score**	0.148
**Variance Inflation Factors (VIFs) for predictors (Socioeconomic Factors)**	
(None) German citizenship	1.14
Parents from Germany (migration status)	1.00
Residence status	1.13
Children	1.43
Homelessness	1.24
Educational level	1.61
Stable relationship	1.04
Social contacts outside sex work	1.44
Third party involvement	1.11
Age at starting sex work	1.16
**Hosmer–Lemeshow Goodness-of-Fit Test**	
χ^2 (df = 3)	1.16
p-value	0.763
**Model fit interpretation**	Non-significant *p* indicates good model fit. Multicollinearity was low (all VIFs < 1.61). Brier score indicates reasonable predictive accuracy.

**Diagnostic Test**	**Result**
**Model type**	Binary logistic regression
**Outcome variable**	Working in Own Apartment setting (yes/no)
**McFadden's Pseudo-R²**	0.141
**Brier Score**	0.089
**Variance Inflation Factors (VIFs) for predictors (Socioeconomic Factors)**	
(None) German citizenship	1.11
Parents from Germany (migration status)	1.00
Residence status	1.10
Children	1.25

Homelessness	1.18
Educational level	1.51
Stable relationship	1.05
Social contacts outside sex work	1.33
Third party involvement	1.11
Age at starting sex work	1.20
**Hosmer–Lemeshow Goodness-of-Fit Test**	
χ^2 (df = 3)	1.71
p-value	0.634
**Model fit interpretation**	Non-significant *p* indicates good model fit. Multicollinearity was low (all VIFs < 1.51). Brier score indicates reasonable predictive accuracy.

3.2.2. Do mental diseases predict specific work settings?

**Diagnostic Test**	**Result**
**Model type**	Binary logistic regression
**Outcome variable**	Working in Street/Car (yes/no)
**McFadden's Pseudo-R²**	0.064
**Variance Inflation Factors (VIFs) across mental disorder models**	
Anxiety Disorders	1.22
Affective Disorders	1.25
OCD	1.19
Eating Disorder	1.11
Somatization	1.08
Addiction	1.07
Sleep Disorder	1.28
PTSD	1.23
**Hosmer–Lemeshow Goodness-of-Fit Test**	
χ^2 (df = 2)	3.45
p-value	0.178
**Model fit interpretation**	Non-significant *p* indicates good model fit. Multicollinearity was low (all VIFs < 1.28).

**Diagnostic Test**	**Result**
**Model type**	Binary logistic regression
**Outcome variable**	Working in Online setting (yes/no)
**McFadden's Pseudo-R²**	0.051

**Variance Inflation Factors (VIFs) across mental disorder models**	
Anxiety Disorders	1.24
Affective Disorders	1.27
OCD	1.17
Eating Disorder	1.10
Somatization	1.07
Addiction	1.07
Sleep Disorder	1.24
PTSD	1.25
**Hosmer–Lemeshow Goodness-of-Fit Test**	
χ^2 (df = 2)	1.89
p-value	0.388
**Model fit interpretation**	Non-significant *p* indicates good model fit. Multicollinearity was low (all VIFs < 1.27).

**Diagnostic Test**	**Result**
**Model type**	Binary logistic regression
**Outcome variable**	Working in Studio setting (yes/no)
**McFadden's Pseudo-R²**	0.028
**Variance Inflation Factors (VIFs) across mental disorder models**	
Anxiety Disorders	1.27
Affective Disorders	1.28
OCD	1.18
Eating Disorder	1.11
Somatization	1.08
Addiction	1.05
Sleep Disorder	1.26
PTSD	1.25
**Hosmer–Lemeshow Goodness-of-Fit Test**	
χ^2 (df = 2)	0.96
p-value	0.617
**Model fit interpretation**	Non-significant *p* indicates good model fit. Multicollinearity was low (all VIFs < 1.28).

**Diagnostic Test**	**Result**
**Model type**	Binary logistic regression
**Outcome variable**	Working in Hotel/Client's Apartment setting (yes/no)
**McFadden's Pseudo-R²**	0.077

**Variance Inflation Factors (VIFs) across mental disorder models**	
Anxiety Disorders	1.23
Affective Disorders	1.22
OCD	1.15
Eating Disorder	1.08
Somatization	1.06
Addiction	1.02
Sleep Disorder	1.25
PTSD	1.23
**Hosmer–Lemeshow Goodness-of-Fit Test**	
χ^2 (df = 2)	1.74
p-value	0.419
**Model fit interpretation**	Non-significant *p* indicates good model fit. Multicollinearity was low (all VIFs < 1.25).

3.2.3. Do self-reported reasons predict the work setting?

**Diagnostic Test**	**Result**
**Model type**	Binary logistic regression
**Outcome variable**	Street/Car
**McFadden's Pseudo-R²**	0.190
**Brier Score**	0.158
**Variance Inflation Factors (VIFs) for predictors (Reasons)**	
Personal Preference	1.20
Financial Reasons	1.16
Supporting their Family	1.13
Supporting their Partner	1.15
Paying off Debt	1.14
Financing Drugs	1.06
Financing their education	1.12
Having no choice	1.26
Being forced by someone	1.21
Being forced by the circumstances (e.g. no alternative to earning well enough with other jobs)	1.19
Other	1.12
**Hosmer–Lemeshow Goodness-of-Fit Test**	
χ^2 (df = 3)	2.96
p-value	0.398

**Model fit interpretation**	Non-significant *p* indicates good model fit. Multicollinearity was low (all VIFs < 1.27). Brier score indicates reasonable predictive accuracy.

**Diagnostic Test**	**Result**
**Model type**	Binary logistic regression
**Outcome variable**	Escort (diverse)
**McFadden's Pseudo-R²**	0.103
**Brier Score**	0.169
**Variance Inflation Factors (VIFs) for predictors (Reasons)**	
Personal Preference	1.19
Financial Reasons	1.18
Supporting their Family	1.11
Supporting their Partner	1.09
Paying off Debt	1.15
Financing Drugs	1.12
Financing their education	1.03
Having no choice	1.27
Being forced by someone	1.08
Being forced by the circumstances (e.g. no alternative to earning well enough with other jobs)	1.28
Other	1.14
**Hosmer–Lemeshow Goodness-of-Fit Test**	
χ^2 (df = 3)	5.30
p-value	0.151
**Model fit interpretation**	Non-significant *p* indicates good model fit. Multicollinearity was low (all VIFs < 1.28). Brier score indicates reasonable predictive accuracy.

**Diagnostic Test**	**Result**
**Model type**	Binary logistic regression
**Outcome variable**	Hotel/Client's Apartmen
**McFadden's Pseudo-R²**	0.092
**Brier Score**	0.219
**Variance Inflation Factors (VIFs) for predictors (Reasons)**	
Personal Preference	1.22
Financial Reasons	1.20
Supporting their Family	1.13

Supporting their Partner	1.11
Paying off Debt	1.08
Financing Drugs	1.03
Financing their education	1.02
Having no choice	1.20
Being forced by someone	1.14
Being forced by the circumstances (e.g. no alternative to earning well enough with other jobs)	1.16
Other	1.11
**Hosmer–Lemeshow Goodness-of-Fit Test**	
χ^2 (df = 3)	2.84
p-value	0.418
**Model fit interpretation**	Non-significant *p* indicates good model fit. Multicollinearity was low (all VIFs < 1.22). Brier score indicates reasonable predictive accuracy.

Diagnostic Test	Result
**Model type**	Binary logistic regression
**Outcome variable**	Online
**McFadden's Pseudo-R²**	0.080
**Brier Score**	0.193
**Variance Inflation Factors (VIFs) for predictors (Reasons)**	
Personal Preference	1.21
Financial Reasons	1.17
Supporting their Family	1.13
Supporting their Partner	1.14
Paying off Debt	1.17
Financing Drugs	1.10
Financing their education	1.05
Having no choice	1.32
Being forced by someone	1.20
Being forced by the circumstances (e.g. no alternative to earning well enough with other jobs)	1.29
Other	1.12
**Hosmer–Lemeshow Goodness-of-Fit Test**	
χ^2 (df = 3)	3.52
p-value	0.318
**Model fit interpretation**	Non-significant *p* indicates good model fit. Multicollinearity was low (all VIFs < 1.32).

	Brier score indicates reasonable predictive accuracy.

**Diagnostic Test**	**Result**
**Model type**	Binary logistic regression
**Outcome variable**	Club
**McFadden's Pseudo-R^2**	0.029
**Brier Score**	0.076
**Variance Inflation Factors (VIFs) for predictors (Reasons)**	
Personal Preference	1.21
Financial Reasons	1.18
Supporting their Family	1.12
Supporting their Partner	1.10
Paying off Debt	1.14
Financing Drugs	1.09
Financing their education	1.04
Having no choice	1.28
Being forced by someone	1.24
Being forced by the circumstances (e.g. no alternative to earning well enough with other jobs)	1.23
Other	1.13
**Hosmer–Lemeshow Goodness-of-Fit Test**	
χ^2 (df = 3)	4.02
p-value	0.260
**Model fit interpretation**	Non-significant *p* indicates good model fit. Multicollinearity was low (all VIFs < 1.28). Brier score indicates reasonable predictive accuracy.

**Diagnostic Test**	**Result**
**Model type**	Binary logistic regression
**Outcome variable**	Working in Brothel setting (yes/no)
**McFadden's Pseudo-R^2**	0.069
**Brier Score**	0.098
**Variance Inflation Factors (VIFs) for predictors (Reasons)**	
Personal Preference	1.23
Financial Reasons	1.20
Supporting their Family	1.14
Supporting their Partner	1.12
Paying off Debt	1.11

Financing Drugs	1.09
Financing their education	1.04
Having no choice	1.26
Being forced by someone	1.22
Being forced by the circumstances (e.g. no alternative to earning well enough with other jobs)	1.22
Other	1.12
**Hosmer–Lemeshow Goodness-of-Fit Test**	
χ^2 (df = 3)	1.38
p-value	0.710
**Model fit interpretation**	Non-significant *p* indicates good model fit. Multicollinearity was low (all VIFs < 1.26). Brier score indicates reasonable predictive accuracy.

**Diagnostic Test**	**Result**
**Model type**	Binary logistic regression
**Outcome variable**	Working in Studio setting (yes/no)
**McFadden's Pseudo-R^2**	0.133
**Brier Score**	0.177
**Variance Inflation Factors (VIFs) for predictors (Reasons)**	
Personal Preference	1.25
Financial Reasons	1.19
Supporting their Family	1.14
Supporting their Partner	1.14
Paying off Debt	1.26
Financing Drugs	1.10
Financing their education	1.04
Having no choice	1.44
Being forced by someone	1.34
Being forced by the circumstances (e.g. no alternative to earning well enough with other jobs)	1.33
Other	1.13
**Hosmer–Lemeshow Goodness-of-Fit Test**	
χ^2 (df = 3)	3.87
p-value	0.275
**Model fit interpretation**	Non-significant *p* indicates good model fit. Multicollinearity was low (all VIFs < 1.44). Brier score indicates reasonable predictive accuracy.

**Diagnostic Test**	**Result**
**Model type**	Binary logistic regression
**Outcome variable**	Working in Own Apartment setting (yes/no)
**McFadden's Pseudo-R^2**	0.048
**Brier Score**	0.101
**Variance Inflation Factors (VIFs) for predictors (Reasons)**	
Personal Preference	1.20
Financial Reasons	1.14
Supporting their Family	1.12
Supporting their Partner	1.11
Paying off Debt	1.16
Financing Drugs	1.13
Financing their education	1.05
Having no choice	1.31
Being forced by someone	1.10
Being forced by the circumstances (e.g. no alternative to earning well enough with other jobs)	1.31
Other	1.10
**Hosmer–Lemeshow Goodness-of-Fit Test**	
χ^2 (df = 3)	17.47
p-value	0.00057
**Model fit interpretation**	Significant *p* indicates poor model fit. Multicollinearity was low (all VIFs < 1.31). Brier score indicates moderate predictive accuracy.

3.3. How are reported healthcare and support needs associated with sex work setting?

**Diagnostic Test**	**Result**
**Model type**	Binary logistic regression
**Outcome variable**	Reported socio-medical need 1: A home/ safe place (yes/no)
**McFadden's Pseudo-R^2**	0.133
**Brier Score**	0.028
**Variance Inflation Factors (VIFs) for predictors (Work Settings)**	
Street/Car	1.05

Escort (diverse)	1.00
Hotel/Client's apartment	1.23
Online	1.27
Club	1.01
Brothel	1.15
Studio	1.03
Own apartment	1.11
**Hosmer–Lemeshow Goodness-of-Fit Test**	
χ^2 (df = 7)	0.449
p-value	0.9996
**Model fit interpretation**	Non-significant *p* indicates excellent model fit. Multicollinearity was very low (all VIFs < 1.27). Brier score indicates high predictive accuracy.

**Diagnostic Test**	**Result**
**Model type**	Binary logistic regression
**Outcome variable**	Reported socio-medical need 2: Protection from physical attacks (yes/no)
**McFadden's Pseudo-R²**	0.122
**Brier Score**	0.207
**Variance Inflation Factors (VIFs) for predictors (Work Settings)**	
Street/Car	1.07
Escort (diverse)	1.17
Hotel/Client's apartment	1.22
Online	1.10
Club	1.02
Brothel	1.06
Studio	1.03
Own apartment	1.03
**Hosmer–Lemeshow Goodness-of-Fit Test**	
χ^2 (df = 8)	9.42
p-value	0.308
**Model fit interpretation**	Non-significant *p* indicates acceptable model fit. Multicollinearity was low (all VIFs < 1.23). Brier score indicates good predictive accuracy.

**Diagnostic Test**	**Result**
**Model type**	Binary logistic regression
**Outcome variable**	Reported socio-medical need 3: Assistance in exiting the business (yes/no)

**McFadden's Pseudo-R^2**	0.111
**Brier Score**	0.199
**Variance Inflation Factors (VIFs) for predictors (Work Settings)**	
Street/Car	1.07
Escort (diverse)	1.16
Hotel/Client's apartment	1.18
Online	1.09
Club	1.03
Brothel	1.04
Studio	1.03
Own apartment	1.04
**Hosmer–Lemeshow Goodness-of-Fit Test**	
χ^2 (df = 8)	7.89
p-value	0.444
**Model fit interpretation**	Non-significant *p* indicates good model fit. Multicollinearity was low (all VIFs < 1.18). Brier score shows good predictive accuracy.

**Diagnostic Test**	**Result**
**Model type**	Binary logistic regression
**Outcome variable**	Reported socio-medical need 4: Therapy to quit drugs and alcohol (yes/no)
**McFadden's Pseudo-R^2**	0.157
**Brier Score**	0.159
**Variance Inflation Factors (VIFs) for predictors (Work Settings)**	
Street/Car	1.068
Escort (diverse)	1.146
Hotel/Client's apartment	1.145
Online	1.097
Club	1.044
Brothel	1.045
Studio	1.052
Own apartment	1.044
**Hosmer–Lemeshow Goodness-of-Fit Test**	
χ^2 (df = 8)	10.243
p-value	0.248
**Model fit interpretation**	Non-significant *p* indicates good model fit. Multicollinearity was low (all VIFs < 1.15). Brier score shows good predictive accuracy.

**Diagnostic Test**	**Result**
**Model type**	Binary logistic regression
**Outcome variable**	Reported socio-medical need 5: Medical support (yes/no)
**McFadden's Pseudo-R²**	0.143
**Brier Score**	0.139
**Variance Inflation Factors (VIFs) for predictors (Work Settings)**	
Street/Car	1.087
Escort (diverse)	1.117
Hotel/Client's apartment	1.136
Online	1.084
Club	1.044
Brothel	1.060
Studio	1.063
Own apartment	1.044
**Hosmer–Lemeshow Goodness-of-Fit Test**	
χ^2 (df = 8)	10.635
p-value	0.223
**Model fit interpretation**	Non-significant *p* indicates good model fit. Multicollinearity was low (all VIFs < 1.14). Brier score shows acceptable predictive accuracy.

**Diagnostic Test**	**Result**
**Model type**	Binary logistic regression
**Outcome variable**	Reported socio-medical need 6: Attorney/ legal support (yes/no)
**McFadden's Pseudo-R²**	0.167
**Brier Score**	0.095
**Variance Inflation Factors (VIFs) for predictors (Work Settings)**	
Street/Car	1.109
Escort (diverse)	1.120
Hotel/Client's apartment	1.090
Online	1.159
Club	1.072
Brothel	1.101
Studio	1.114
Own apartment	1.085
**Hosmer–Lemeshow Goodness-of-Fit Test**	
χ^2 (df = 3)	6.251

p-value	0.100
**Model fit interpretation**	Non-significant *p* indicates acceptable model fit. Multicollinearity was low (all VIFs < 1.16). Brier score shows good predictive accuracy.

**Diagnostic Test**	**Result**
**Model type**	Binary logistic regression
**Outcome variable**	Reported socio-medical need 7: Residence permit/ legal status (yes/no)
**McFadden's Pseudo-R²**	0.182
**Brier Score**	0.078
**Variance Inflation Factors (VIFs) for predictors (Work Settings)**	
Street/Car	1.166
Escort (diverse)	1.099
Hotel/Client's apartment	1.072
Online	1.194
Club	1.072
Brothel	1.134
Studio	1.159
Own apartment	1.100
**Hosmer–Lemeshow Goodness-of-Fit Test**	
χ^2 (df = 8)	11.737
p-value	0.163
**Model fit interpretation**	Non-significant *p* indicates acceptable model fit. Multicollinearity was low (all VIFs < 1.20). Brier score shows good predictive accuracy.

**Diagnostic Test**	**Result**
**Model type**	Binary logistic regression
**Outcome variable**	Reported socio-medical need 8: Better and safer working conditions (yes/no)
**McFadden's Pseudo-R²**	0.156
**Brier Score**	0.159
**Variance Inflation Factors (VIFs) for predictors (Work Settings)**	
Street/Car	1.068
Escort (diverse)	1.146
Hotel/Client's apartment	1.145
Online	1.097
Club	1.044

Brothel	1.045
Studio	1.052
Own apartment	1.044
**Hosmer–Lemeshow Goodness-of-Fit Test**	
χ^2 (df = 8)	10.243
p-value	0.248
**Model fit interpretation**	Non-significant *p* indicates acceptable model fit. Multicollinearity was low (all VIFs < 1.16). Brier score shows good predictive accuracy.

**Diagnostic Test**	**Result**
**Model type**	Binary logistic regression
**Outcome variable**	Reported socio-medical need 9: Recognition of sex work as a normal occupation/work activity (yes/no)
**McFadden's Pseudo-R²**	0.161
**Brier Score**	0.038
**Variance Inflation Factors (VIFs) for predictors (Work Settings)**	
Street/Car	1.194
Escort (diverse)	1.063
Hotel/Client's apartment	1.062
Online	1.081
Club	1.000
Brothel	1.055
Studio	1.191
Own apartment	1.024
**Hosmer–Lemeshow Goodness-of-Fit Test**	
χ^2 (df = 8)	14.631
p-value	0.067
**Model fit interpretation**	Non-significant *p* indicates acceptable model fit. Multicollinearity was low (all VIFs < 1.20). Brier score shows good predictive accuracy.
