# Supplementary material for: Quality of life, mental health, and socio-demographic differences across sex work settings: implications for specialized healthcare and support services
Source: Front Public Health. 2025 Dec 4;13:1703735. doi: 10.3389/fpubh.2025.1703735 (PMC12711543; doi:10.3389/fpubh.2025.1703735)
Supplement: Supplementary file 3 [file Supplementary_file_3.pdf]

R version 4.3.3 (2024-02-29) -- "Angel Food Cake"
Copyright (C) 2024 The R Foundation for Statistical Computing
Platform: x86_64-apple-darwin20 (64-bit)

**Code:**

```
library(tidyverse)
library(haven)
library(broom)
library(writexl)
library(MASS
library(lmtest)
location_vars <- c("Car_Street", "Diverse_Escort", "Client_Hotel", "online", "club", "brothel", "studio",
"own_apartment")
qol_outcomes <- c("SF12_PCS12", "SF12_MCS12")
data <- data %>%
  mutate(across(all_of(location_vars), ~ factor(.x, levels = c(2, 1))))
fit_model <- function(outcome) {
  form <- as.formula(paste(outcome, "~", paste(location_vars, collapse = " + ")))
  ols_model <- lm(form, data = data)

  bp_test <- bptest(ols_model)

  if (bp_test$p.value < 0.05) {
    message(paste(outcome, ": OLS assumptions violated -> using RLM"))
    rlm_model <- rlm(form, data = data)
    return(rlm_model)
  } else {
    return(ols_model)
  }
}
model_results_qol <- map(qol_outcomes, fit_model)
extract_model_info <- function(model) {
  # For RLM, broom::glance doesn't fully work, so handle separately
  if (inherits(model, "rlm")) {
    tidy_df <- broom::tidy(model) %>%
      mutate(conf.low = estimate - 1.96*std.error,
             conf.high = estimate + 1.96*std.error)
    glance_df <- tibble(
      sigma = summary(model)$sigma,
      residual_df = model$df.residual
    )
  } else {
    tidy_df <- broom::tidy(model, conf.int = TRUE)
    glance_df <- broom::glance(model)
  }
  list(coefficients = tidy_df, model_info = glance_df)
}
model_summaries_qol <- map(model_results_qol, extract_model_info)
names(model_summaries_qol) <- qol_outcomes
```

**Physical Component of QoL:**

| Term | Estimate | Std. Error | Statistic | Conf. Low | Conf. High | p-value |
|---|---|---|---|---|---|---|
| (Intercept) | 52.9 | 0.903 | 58.6 | 51.1 | 54.6 | 0.000000 |
| Car/Street | -3.41 | 1.01 | -3.38 | -5.39 | -1.43 | 0.000727 |
| Escort/Diverse | 0.773 | 1.05 | 0.733 | -1.29 | 2.84 | 0.464 |
| Client´s apartment/ Hotel | -1.50 | 0.966 | -1.56 | -3.40 | 0.390 | 0.120 |
| online | -0.849 | 0.984 | -0.863 | -2.78 | 1.08 | 0.388 |
| club | -1.88 | 1.55 | -1.22 | -4.92 | 1.15 | 0.224 |

| | | | | | | |
|---|---|---|---|---|---|---|
| brothel | -1.26 | 1.37 | -0.916 | -3.95 | 1.43 | 0.359 |
| studio | -0.456 | 0.970 | -0.470 | -2.36 | 1.44 | 0.638 |
| Own apartment | 0.0823 | 1.37 | 0.0601 | -2.60 | 2.76 | 0.952 |

| $R^2$ | adj. $R^2$ | sigma | Statistic | p. value | df | logLik | AIC | BIC | deviance | df.residual | nobs |
|---|---|---|---|---|---|---|---|---|---|---|---|
| 0.0372 | 0.0172 | 9.05 | 1.86 | 0.0658 | 8 | -- 1419. | 2855. | 2891. | 31425 | 384 | 393 |

**Mental Component of QoL:**

| term | estimate | std.error | statistic | p.value | conf.low | conf.high |
|---|---|---|---|---|---|---|
| (Intercept) | 45.40 | 1.19 | 38.04 | 2.5e-132 | 43.05 | 47.74 |
| Car/Street | -3.26 | 1.33 | -2.44 | 0.015 | -5.88 | -0.64 |
| Escort/Diverse | 0.23 | 1.39 | 0.17 | 0.87 | -2.51 | 2.97 |
| Client´s apartment/ Hotel | -3.52 | 1.28 | -2.76 | 0.00606 | -6.04 | -1.01 |
| online | -0.75 | 1.30 | -0.58 | 0.56 | -3.31 | 1.80 |
| club | -6.04 | 2.05 | -2.95 | 0.00336 | -10.07 | -2.02 |
| brothel | 0.98 | 1.82 | 0.54 | 0.59 | -2.60 | 4.55 |
| studio | 2.94 | 1.28 | 2.30 | 0.0222 | 0.42 | 5.46 |
| Own apartment | -1.65 | 1.81 | -0.91 | 0.36 | -5.20 | 1.91 |

| $R^2$ | adj. $R^2$ | sigma | statistic | p.value | df | logLik | AIC | BIC | deviance | df.residual | nobs |
|---|---|---|---|---|---|---|---|---|---|---|---|
| 0,08637 | 0,067336 | 11,31186 | 4,537694 | 2,72E-05 | 8 | -1506,45 | 3032,901 | 3072,639 | 49135,97 | 384 | 393 |

**Results adjusted model for physical component of quality of life:**

**Physical Component:**

| term | estimate | std.error | statistic | p.value | conf.low | conf.high | OR_like | OR_low | OR_high |
|---|---|---|---|---|---|---|---|---|---|
| (Intercept) | 48.39 | 9.92 | 4.88 | 0.0000132 | 28.43 | 68.36 | 1.04e+21 | 2.22e+12 | 4.86e+29 |
| Workingdays per Week | -0.18 | 0.51 | -0.36 | 0.72 | -1.21 | 0.85 | 0.83 | 0.30 | 2.33 |
| Burden Activity | -2.23 | 1.49 | -1.49 | 0.14 | -5.24 | 0.77 | 0.11 | 0.01 | 2.17 |
| Burden Working Hours | 2.34 | 1.09 | 2.14 | 0.04 | 0.14 | 4.54 | 10.38 | 1.15 | 93.58 |
| Burden Circumstances | -2.56 | 1.26 | -2.03 | 0.05 | -5.11 | -0.02 | 0.08 | 0.01 | 0.98 |
| Burden Financial Exploitation | 0.12 | 1.10 | 0.11 | 0.91 | -2.10 | 2.33 | 1.13 | 0.12 | 10.32 |
| Burden Coercion | -1.73 | 1.30 | -1.33 | 0.19 | -4.36 | 0.90 | 0.18 | 0.01 | 2.45 |
| Burden Violence | 3.86 | 1.22 | 3.15 | 0.00284 | 1.40 | 6.32 | 47.40 | 4.04 | 556.66 |
| Burden Demands | -3.60 | 1.38 | -2.60 | 0.01237 | -6.37 | -0.82 | 0.03 | 0.002 | 0.44 |
| Burden DoubleLife | 1.48 | 0.99 | 1.49 | 0.14 | -0.52 | 3.47 | 4.37 | 0.60 | 32.13 |
| Burden Relationship | -2.98 | 1.29 | -2.30 | 0.03 | -5.58 | -0.37 | 0.05 | 0.004 | 0.69 |
| Burden Financial Dependence | 1.60 | 1.39 | 1.14 | 0.26 | -1.21 | 4.40 | 4.93 | 0.30 | 81.49 |
| Burden Arrest | -0.15 | 1.20 | -0.12 | 0.90 | -2.57 | 2.28 | 0.86 | 0.08 | 9.73 |

| | | | | | | | | |
|---|---|---|---|---|---|---|---|---|
| Burden Sexual Difficulties | -0.61 | 1.10 | -0.55 | 0.58 | -2.82 | 1.60 | 0.54 | 0.06 | 4.95 |
| Burden Guilt or Shame | 2.34 | 1.12 | 2.08 | 0.04 | 0.08 | 4.59 | 10.34 | 1.08 | 98.74 |
| Burden Health | -2.94 | 1.16 | -2.53 | 0.015 | -5.28 | -0.60 | 0.05 | 0.005 | 0.55 |
| Wellbeing in Sex Work | -0.06 | 0.83 | -0.07 | 0.94 | -1.74 | 1.62 | 0.94 | 0.18 | 5.03 |
| Experience Clients | -0.57 | 0.74 | -0.78 | 0.44 | -2.05 | 0.91 | 0.56 | 0.13 | 2.47 |
| Income Monthly | 1.15 | 1.29 | 0.89 | 0.38 | -1.44 | 3.74 | 3.15 | 0.24 | 42.07 |
| Car/Street | 6.35 | 3.14 | 2.02 | 0.05 | 0.03 | 12.66 | 570.32 | 1.03 | 314428.3 |
| Diverse/Escort | 0.86 | 3.33 | 0.26 | 0.80 | -5.83 | 7.55 | 2.36 | 0.003 | 1906.52 |
| Client’s apartment/Hotel | 2.37 | 2.17 | 1.09 | 0.28 | -1.99 | 6.74 | 10.73 | 0.14 | 842.81 |
| Online | -0.95 | 2.72 | -0.35 | 0.73 | -6.42 | 4.51 | 0.39 | 0.002 | 91.36 |
| Club | 0.08 | 4.88 | 0.02 | 0.99 | -9.74 | 9.89 | 1.08 | 0.000059 | 19788.4 |
| Brothel | 0.15 | 3.24 | 0.05 | 0.96 | -6.36 | 6.66 | 1.16 | 0.002 | 780.59 |
| Studio | 1.08 | 2.90 | 0.37 | 0.71 | -4.76 | 6.92 | 2.94 | 0.01 | 1013.03 |
| Own apartment | -3.27 | 2.66 | -1.23 | 0.22 | -8.62 | 2.07 | 0.04 | 0.000181 | 7.94 |
| Migration Background | 9.51 | 6.03 | 1.58 | 0.12 | -2.63 | 21.65 | 13522.08 | 0.07 | 2.53e+09 |
| Not holding German Citizenship | 3.72 | 4.56 | 0.82 | 0.42 | -5.45 | 12.89 | 41.18 | 0.004 | 395469.5 |
| Lower Secondary School Certificate | -3.12 | 3.01 | -1.04 | 0.30 | -9.18 | 2.93 | 0.04 | 0.000104 | 18.65 |
| Intermediate Secondary School Leaving Certificate | 6.20 | 3.07 | 2.02 | 0.049 | 0.02 | 12.38 | 493.87 | 1.02 | 238630.8 |
| Completed apprenticeship | 6.19 | 4.49 | 1.38 | 0.17 | -2.84 | 15.22 | 487.47 | 0.06 | 4080725 |
| Entrance qualification for universities of applied science (“Fachabitur”, “Fachhochschulreife”) | -1.37 | 11.06 | -0.12 | 0.90 | -23.64 | 20.89 | 0.25 | 5.42e-11 | 1.18e+09 |
| University entrance qualification, high school degree (“Abitur”) | -0.40 | 3.68 | -0.11 | 0.91 | -7.81 | 7.01 | 0.67 | 0.000405 | 1110.17 |
| University degree | 1.23 | 3.17 | 0.39 | 0.70 | -5.15 | 7.60 | 3.41 | 0.006 | 2004.87 |
| Other degree | 6.06 | 11.30 | 0.54 | 0.59 | -16.68 | 28.81 | 430.26 | 5.7e-08 | 3.25e+12 |
| Not being Homelessness | 11.34 | 2.10 | 5.40 | 0.00000228 | 7.11 | 15.56 | 83814.38 | 1223.64 | 5740929 |

| | | | | | | | | | |
|---|---|---|---|---|---|---|---|---|---|
| Not having Children | -4.60 | 2.36 | -1.95 | 0.06 | -9.34 | 0.14 | 0.01 | 0.000088 | 1.16 |
| No Stable Relationship | -5.91 | 2.12 | -2.78 | 0.008 | -10.18 | -1.64 | 0.003 | 0.0000381 | 0.19 |
| Vaginal intercourse | -1.36 | 3.33 | -0.41 | 0.68 | -8.07 | 5.34 | 0.26 | 0.000313 | 209.28 |
| Anal intercourse | -0.28 | 2.44 | -0.11 | 0.91 | -5.19 | 4.64 | 0.76 | 0.006 | 103.37 |
| Oral sex | 1.68 | 2.72 | 0.62 | 0.54 | -3.79 | 7.14 | 5.34 | 0.023 | 1263.78 |
| Others | 0.32 | 3.01 | 0.11 | 0.92 | -5.74 | 6.38 | 1.37 | 0.003 | 587.97 |
| Not applicable | 2.55 | 7.23 | 0.35 | 0.73 | -12.01 | 17.10 | 12.75 | 0.00000607 | 26776593 |

| **r.squared** | **adj.r.squared** | **sigma** | **statistic** | **p.value** | **df** | **logLik** | **AIC** | **BIC** | **deviance** | **df.residual** | **nobs** |
|---|---|---|---|---|---|---|---|---|---|---|---|
| 0,750198 | 0,516688 | 6,766984 | 3,212702 | 7,42E-05 | 43 | -269,587 | 629,1738 | 741,6652 | 2106,436 | 46 | 90 |

**Mental Component:**

| **term** | **estimate** | **std.error** | **statistic** | **p.value** | **conf.low** | **conf.high** | **OR_like** | **OR_low** | **OR_high** |
|---|---|---|---|---|---|---|---|---|---|
| (Intercept) | 62.67 | 14.44 | 4.34 | 0.0000777 | 33.59 | 91.74 | 1.64e+27 | 3.87e+14 | 6.95e+39 |
| Workingdays per Week | 0.49 | 0.74 | 0.66 | 0.51 | -1.01 | 1.99 | 1.64 | 0.37 | 7.33 |
| Burden Activity | -2.78 | 2.18 | -1.28 | 0.21 | -7.16 | 1.60 | 0.06 | 0.000773 | 4.93 |
| Burden Working Hours | 0.62 | 1.59 | 0.39 | 0.70 | -2.58 | 3.83 | 1.86 | 0.08 | 45.88 |
| Burden Circumstances | -0.38 | 1.84 | -0.21 | 0.84 | -4.09 | 3.32 | 0.68 | 0.02 | 27.72 |
| Burden Financial Exploitation | -0.38 | 1.60 | -0.23 | 0.82 | -3.60 | 2.85 | 0.69 | 0.03 | 17.30 |
| Burden Coercion | 2.08 | 1.90 | 1.10 | 0.28 | -1.74 | 5.91 | 8.02 | 0.17 | 367.40 |
| Burden Violence | -1.11 | 1.78 | -0.62 | 0.54 | -4.70 | 2.48 | 0.33 | 0.00914 | 11.93 |
| Burden Demands | -1.42 | 2.01 | -0.71 | 0.48 | -5.47 | 2.63 | 0.24 | 0.00423 | 13.87 |
| Burden DoubleLife | -0.79 | 1.44 | -0.55 | 0.59 | -3.69 | 2.11 | 0.45 | 0.025 | 8.29 |
| Burden Relationship | -3.12 | 1.88 | -1.66 | 0.10 | -6.91 | 0.67 | 0.04 | 0.001 | 1.95 |
| Burden Financial Dependence | -0.14 | 2.03 | -0.07 | 0.95 | -4.22 | 3.95 | 0.87 | 0.015 | 51.80 |
| Burden Arrest | 2.38 | 1.75 | 1.36 | 0.18 | -1.15 | 5.91 | 10.80 | 0.32 | 368.16 |
| Burden Sexual Difficulties | 0.83 | 1.60 | 0.52 | 0.61 | -2.39 | 4.05 | 2.29 | 0.09 | 57.27 |
| Burden Guilt or Shame | -0.37 | 1.63 | -0.23 | 0.82 | -3.66 | 2.92 | 0.69 | 0.03 | 18.50 |
| Burden Health | -3.23 | 1.69 | -1.91 | 0.06 | -6.64 | 0.17 | 0.04 | 0.00130 | 1.19 |
| Wellbeing in Sex Work | 0.42 | 1.21 | 0.34 | 0.73 | -2.02 | 2.86 | 1.52 | 0.13 | 17.46 |
| Experience Clients | -0.32 | 1.07 | -0.30 | 0.77 | -2.48 | 1.83 | 0.73 | 0.08 | 6.26 |

| | | | | | | | | | |
|---|---|---|---|---|---|---|---|---|---|
| Income Monthly | -0.28 | 1.88 | -0.15 | 0.88 | -4.05 | 3.50 | 0.76 | 0.02 | 32.97 |
| Car/Street | 2.57 | 4.57 | 0.56 | 0.58 | -6.63 | 11.76 | 13.03 | 0.00133 | 128036.4 |
| Diverse/Escort | -6.30 | 4.84 | -1.30 | 0.20 | -16.05 | 3.45 | 0.00 | 1.07e-07 | 31.35 |
| Client's apartment/Hotel | 0.38 | 3.16 | 0.12 | 0.91 | -5.98 | 6.73 | 1.46 | 0.003 | 839.90 |
| Online | -0.39 | 3.96 | -0.10 | 0.92 | -8.35 | 7.58 | 0.68 | 0.000237 | 1953.55 |
| Club | 8.34 | 7.10 | 1.17 | 0.25 | -5.95 | 22.64 | 4200.08 | 0.003 | 6.78e+09 |
| Brothel | 2.39 | 4.71 | 0.51 | 0.61 | -7.09 | 11.87 | 10.91 | 0.001 | 143362.6 |
| Studio | -5.00 | 4.23 | -1.18 | 0.24 | -13.51 | 3.50 | 0.01 | 1.36e-06 | 33.24 |
| Own apartment | -3.47 | 3.87 | -0.90 | 0.37 | -11.25 | 4.32 | 0.03 | 1.3e-05 | 75.00 |
| Migration Background | 1.22 | 8.78 | 0.14 | 0.89 | -16.46 | 18.90 | 3.39 | 7.12e-08 | 1.62e+08 |
| Not holding German Citizenship | -7.39 | 6.63 | -1.11 | 0.27 | -20.74 | 5.97 | 0.00 | 9.84e-10 | 390.93 |
| Lower Secondary School Certificate | 5.64 | 4.38 | 1.29 | 0.20 | -3.17 | 14.45 | 280.88 | 0.04 | 1885298 |
| Intermediate Secondary School Leaving Certificate | 4.18 | 4.47 | 0.94 | 0.35 | -4.82 | 13.18 | 65.43 | 0.008 | 530457 |
| Completed apprenticeship | -0.28 | 6.53 | -0.04 | 0.97 | -13.43 | 12.88 | 0.76 | 1.47e-06 | 391555 |
| Entrance qualification for universities of applied science ("Fachabitur", "Fachhochschulreife") | 18.21 | 16.11 | 1.13 | 0.26 | -14.22 | 50.63 | 80757477 | 6.69e-07 | 9.74e+21 |
| University entrance qualification, high school degree ("Abitur") | 1.89 | 5.36 | 0.35 | 0.73 | -8.90 | 12.69 | 6.63 | 0.000136 | 323014.4 |
| University degree | 3.91 | 4.61 | 0.85 | 0.40 | -5.38 | 13.20 | 49.84 | 0.00562 | 537737 |
| Other degree | -10.58 | 16.46 | -0.64 | 0.52 | -43.71 | 22.54 | 0.00 | 1.04e-19 | 6.15e+09 |
| Not being Homelessness | 2.36 | 3.06 | 0.77 | 0.44 | -3.79 | 8.52 | 10.63 | 0.023 | 5009.33 |
| Not having Children | -3.79 | 3.43 | -1.10 | 0.28 | -10.69 | 3.12 | 0.02 | 2.27e-05 | 22.63 |
| No Stable Relationship | -2.37 | 3.09 | -0.77 | 0.45 | -8.59 | 3.85 | 0.09 | 0.000187 | 47.06 |
| Vaginal intercourse | -1.63 | 4.85 | -0.34 | 0.74 | -11.40 | 8.13 | 0.20 | 1.12e-05 | 3410.92 |
| Anal intercourse | 4.51 | 3.55 | 1.27 | 0.21 | -2.64 | 11.67 | 91.08 | 0.0711 | 116669.6 |
| Oral sex | -1.47 | 3.95 | -0.37 | 0.71 | -9.43 | 6.49 | 0.23 | 7.99e-05 | 656.51 |

| | | | | | | | | | |
|---|---|---|---|---|---|---|---|---|---|
| Others | 3.70 | 4.38 | 0.84 | 0.40 | -5.13 | 12.52 | 40.32 | 0.00594 | 273708.7 |
| Not applicable | 15.46 | 10.53 | 1.47 | 0.15 | -5.74 | 36.66 | 5168108 | 0.00321 | 8.32e+15 |

| $R^2$ | adj. $R^2$ | sigma | statistic | p.value | df | logLik | AIC | BIC | deviance | df.residual | nobs |
|---|---|---|---|---|---|---|---|---|---|---|---|
| 0,61346 | 0,25213 | 9,854705 | 1,697782 | 0,039793 | 43 | -303,417 | 696,8346 | 809,326 | 4467,299 | 46 | 90 |
