# Supplementary material for: Quality of life, mental health, and socio-demographic differences across sex work settings: implications for specialized healthcare and support services
Source: Front Public Health. 2025 Dec 4;13:1703735. doi: 10.3389/fpubh.2025.1703735 (PMC12711543; doi:10.3389/fpubh.2025.1703735)
Supplement: Supplementary file 4 [file Supplementary_file_4.pdf]

R version 4.3.3 (2024-02-29) -- "Angel Food Cake"
Copyright (C) 2024 The R Foundation for Statistical Computing
Platform: x86_64-apple-darwin20 (64-bit)

Setting as a Predictor of Mental Diseases

Code:

```
library(tidyverse)
library(openxlsx)
results_list <- map(outcomes, function(outcome) {
  form <- as.formula(paste0(outcome, " ~ Car_Street + Diverse_Escort + Client_Hotel + online + club + brothel +
studio + own_apartment"))
  mod <- glm(form, data = data, family = binomial)
  coefs <- summary(mod)$coefficients
  ORs <- exp(coefs[, "Estimate"])
  CI <- exp(confint.default(mod))
  pvals <- coefs[, "Pr(>|z|)"]
  zvals <- coefs[, "z value"]
  estimates <- coefs[, "Estimate"]
  std_err <- coefs[, "Std. Error"]
  tibble(
    Outcome = outcome,
    Variable = rownames(coefs),
    Estimate = estimates,
    Std_Error = std_err,
    z_value = zvals,
    p_value = pvals,
    OR = ORs,
    CI_lower = CI[,1],
    CI_upper = CI[,2]
  )
})
results_df <- bind_rows(results_list)
results_df <- results_df %>%
  mutate(across(c(Estimate, Std_Error, z_value, p_value, OR, CI_lower, CI_upper),
                ~ format(., decimal.mark = ",", scientific = FALSE)))
```

Outcome	Variable	Estimate	Std_Error	z_value	p_value	OR	CI_lower	CI_upper
Anxiety Disorder	(Intercept)	-1.39	0.28	-4.97	6.55e-07	0.25	0.14	0.43
Anxiety Disorder	Car/Street	0.22	0.27	0.81	0.42	1.25	0.73	2.13
Anxiety Disorder	Diverse/ Escort	-0.23	0.30	-0.74	0.46	0.80	0.44	1.45
Anxiety Disorder	Client´s apartment/ Hotel	0.93	0.28	3.28	0.00104	2.54	1.45	4.43
Anxiety Disorder	Online	0.99	0.29	3.45	0.00056	2.70	1.53	4.74
Anxiety Disorder	Club	1.01	0.45	2.22	0.0265	2.74	1.12	6.68
Anxiety Disorder	Brothel	0.09	0.41	0.23	0.82	1.10	0.49	2.47
Anxiety Disorder	Studio	-0.73	0.29	-2.50	0.0124	0.48	0.27	0.85

Anxiety Disorder	Own apartment	-0.04	0.39	-0.09	0.93	0.97	0.45	2.09
Affective Disorder	(Intercept)	-1.85	0.33	-5.55	2.92e-08	0.16	0.08	0.30
Affective Disorder	Car/Street	-0.01	0.33	-0.03	0.97	0.99	0.52	1.87
Affective Disorder	Diverse/ Escort	-0.77	0.37	-2.07	0.0386	0.46	0.22	0.96
Affective Disorder	Client´s apartment/ Hotel	1.20	0.34	3.52	0.00043	3.33	1.70	6.49
Affective Disorder	Online	1.31	0.35	3.73	0.00019	3.71	1.87	7.39
Affective Disorder	Club	1.05	0.53	1.97	0.04896	2.86	1.00	8.14
Affective Disorder	Brothel	0.02	0.47	0.04	0.97	1.02	0.40	2.58
Affective Disorder	Studio	0.06	0.32	0.17	0.86	1.06	0.56	1.99
Affective Disorder	Own apartment	0.37	0.43	0.86	0.39	1.45	0.63	3.35
OCD	(Intercept)	-2.66	0.39	-6.77	1.32e-11	0.07	0.03	0.15
OCD	Car/Street	-0.43	0.44	-0.97	0.33	0.65	0.27	1.55
OCD	Diverse/ Escort	0.48	0.41	1.18	0.24	1.62	0.73	3.58
OCD	Client´s apartment/ Hotel	0.37	0.41	0.90	0.37	1.45	0.65	3.23
OCD	Online	-0.77	0.46	-1.66	0.0973	0.47	0.19	1.15
OCD	Club	1.59	0.46	3.44	0.00058	4.92	1.99	12.19
OCD	Brothel	-0.22	0.59	-0.37	0.72	0.81	0.25	2.57
OCD	Studio	0.03	0.40	0.07	0.94	1.03	0.47	2.24
OCD	Own apartment	1.10	0.48	2.32	0.0205	3.01	1.19	7.66
Eating Disorder	(Intercept)	-3.02	0.46	-6.51	7.47e-11	0.05	0.02	0.12
Eating Disorder	Car/Street	-0.61	0.55	-1.11	0.27	0.55	0.19	1.59
Eating Disorder	Diverse/ Escort	0.96	0.46	2.08	0.0379	2.61	1.06	6.45
Eating Disorder	Client´s apartment/ Hotel	0.24	0.48	0.49	0.63	1.27	0.49	3.26
Eating Disorder	Online	-0.26	0.49	-0.52	0.60	0.77	0.29	2.03
Eating Disorder	Club	0.71	0.62	1.15	0.25	2.03	0.61	6.83
Eating Disorder	Brothel	0.23	0.68	0.34	0.74	1.26	0.33	4.79
Eating Disorder	Studio	-0.10	0.46	-0.21	0.83	0.91	0.37	2.25
Eating Disorder	Own apartment	1.42	0.50	2.82	0.00485	4.15	1.54	11.15
Somatization	(Intercept)	-3.40	0.50	-6.78	1.18e-11	0.03	0.01	0.09
Somatization	Car/Street	0.44	0.52	0.85	0.40	1.55	0.56	4.30

Somatization	Diverse/ Escort	0.30	0.55	0.55	0.58	1.35	0.46	3.97
Somatization	Client´s apartment/ Hotel	0.11	0.53	0.21	0.83	1.12	0.39	3.20
Somatization	Online	0.02	0.54	0.04	0.97	1.02	0.36	2.93
Somatization	Club	0.31	0.78	0.39	0.70	1.36	0.29	6.30
Somatization	Brothel	-0.07	0.78	-0.09	0.93	0.93	0.20	4.29
Somatization	Studio	0.17	0.52	0.33	0.74	1.19	0.43	3.29
Somatization	Own apartment	0.34	0.67	0.51	0.61	1.41	0.38	5.28
Addiction	(Intercept)	-2.64	0.35	-7.52	5.42e-14	0.07	0.04	0.14
Addiction	Car/Street	1.06	0.30	3.59	0.00034	2.90	1.62	5.19
Addiction	Diverse/ Escort	-0.13	0.34	-0.39	0.70	0.88	0.45	1.71
Addiction	Client´s apartment/ Hotel	1.51	0.35	4.31	1.62e-05	4.51	2.27	8.95
Addiction	Online	0.16	0.34	0.48	0.63	1.18	0.60	2.30
Addiction	Club	1.02	0.47	2.16	0.0307	2.77	1.10	6.98
Addiction	Brothel	-0.85	0.65	-1.31	0.19	0.43	0.12	1.53
Addiction	Studio	-0.49	0.34	-1.43	0.15	0.61	0.32	1.20
Addiction	Own apartment	0.54	0.40	1.34	0.18	1.72	0.78	3.78
Sleep Disorder	(Intercept)	-1.99	0.29	-6.75	1.50e-14	0.14	0.08	0.24
Sleep Disorder	Car/Street	0.18	0.31	0.57	0.57	1.19	0.65	2.20
Sleep Disorder	Diverse/ Escort	-0.28	0.35	-0.80	0.42	0.76	0.38	1.49
Sleep Disorder	Client´s apartment/ Hotel	0.33	0.32	1.03	0.30	1.38	0.75	2.57
Sleep Disorder	Online	0.55	0.31	1.80	0.0721	1.73	0.95	3.15
Sleep Disorder	Club	0.15	0.49	0.31	0.75	1.17	0.45	3.04
Sleep Disorder	Brothel	-0.31	0.51	-0.60	0.55	0.74	0.27	1.99
Sleep Disorder	Studio	-0.20	0.32	-0.64	0.52	0.82	0.44	1.52
Sleep Disorder	Own apartment	0.72	0.38	1.89	0.0583	2.05	0.98	4.30
PTBS	(Intercept)	-2.11	0.30	-6.99	2.70e-12	0.12	0.07	0.22
PTBS	Car/Street	0.17	0.32	0.51	0.61	1.18	0.63	2.23
PTBS	Diverse/ Escort	0.29	0.33	0.88	0.38	1.34	0.70	2.56
PTBS	Client´s apartment/ Hotel	0.58	0.33	1.78	0.0750	1.79	0.94	3.40
PTBS	Online	0.08	0.32	0.25	0.80	1.09	0.58	2.05
PTBS	Club	0.76	0.44	1.72	0.0856	2.14	0.90	5.10
PTBS	Brothel	0.31	0.43	0.72	0.47	1.36	0.59	3.17
PTBS	Studio	-0.00	0.32	-0.01	0.99	1.00	0.53	1.86

PTBS	Own apartment	0.93	0.39	2.42	0.0156	2.54	1.19	5.41
