# Supplementary material for: Quality of life, mental health, and socio-demographic differences across sex work settings: implications for specialized healthcare and support services
Source: Front Public Health. 2025 Dec 4;13:1703735. doi: 10.3389/fpubh.2025.1703735 (PMC12711543; doi:10.3389/fpubh.2025.1703735)
Supplement: Supplementary file 5 [file Supplementary_file_5.pdf]

R version 4.3.3 (2024-02-29) -- "Angel Food Cake"
Copyright (C) 2024 The R Foundation for Statistical Computing
Platform: x86_64-apple-darwin20 (64-bit)

Setting as a Predictor of Working Conditions

Burden Variables Code:

```
library(tidyverse)
library(broom)
library(writexl)
library(MASS)   # for polr()

burden_vars <- c(
  "Belastung_Tätigkeit", "Belastung_STD", "Belastung_Umstände",
  "Belastung_finanzielle_Ausbeutung", "Belastung_Zwang", "Belastung_Gewalt",
  "Belastung_Forderungen", "Belastung_Doppelleben", "Belastung_Beziehung",
  "Belastung_finanz_Abh", "Belastung_Festnahme", "Belastung_sex_Schwierigk",
  "Belastung_Schuld_Scham", "Belastung_Gesundheit", "Belastung_andere"
)

workplace_vars <- c("Car_Street", "Diverse_Escort", "Client_Hotel",
                    "online", "club", "brothel", "studio", "own_apartment")

data <- data %>%
  mutate(across(all_of(workplace_vars), ~ relevel(as.factor(.), ref = "2")))

data <- data %>%
  mutate(across(all_of(burden_vars), ~ ordered(.)))

results_burden <- list()

for (bur in burden_vars) {
  cat("------------------------------------------------------\n")
  cat("Modell für Burden:", bur, "\n")

  formula_polr <- as.formula(paste(bur, "~", paste(workplace_vars, collapse = " + ")))

  tryCatch({
    model <- MASS::polr(formula_polr, data = data, Hess = TRUE)
    tidy_out <- broom::tidy(model, conf.int = TRUE, conf.level = 0.95, exponentiate = TRUE) %>%
      filter(!str_detect(term, "\\|")) %>%  # exclude intercept thresholds
      mutate(
        Beta = round(log(estimate), 3),  # log(OR)
        OR = round(estimate, 2),
        CI_Lower = round(conf.low, 2),
        CI_Upper = round(conf.high, 2),
        P_Value = round(p.value, 4),
        Burden = bur
      ) %>%
      select(Burden, term, Beta, P_Value, OR, CI_Lower, CI_Upper)

    results_burden[[bur]] <- tidy_out
  }, error = function(e) {
    cat("Fehler bei", bur, ":", e$message, "\n")
  })
}
```

```
results_burden_df <- bind_rows(results_burden)
```

term	estimate	std.error	statistic	P value	Lower 95% CI	Upper 95% CI
**Burden related to circumstances such as competition, long waiting hours or weather conditions**						
Car/Street	-1,7163348	0,23073977	-7,4384003	**1,0192E-13**	-2,1685848	-1,2640849
Escort/ Diverse	0,22397155	0,23415324	0,95651697	0,33881111	-0,2349688	0,68291191
Client´s apartment/ Hotel	-0,3721579	0,21635072	-1,7201604	0,08540329	-0,7962053	0,05188947
Online	0,52112365	0,22472686	2,31892014	**0,02039937**	0,080659	0,9615883
Club	-0,5413124	0,3481678	-1,5547456	0,12000667	-1,2237212	0,14109653
Brothel	-0,1352763	0,31107798	-0,4348631	0,66366177	-0,7449892	0,47443649
Studio	0,51960772	0,2230298	2,32976818	**0,01981841**	0,08246932	0,95674612
Own apartment	-0,080633	0,2995754	-0,2691576	0,7878084	-0,6678008	0,50653479
1|2	-2,9897986	1,31573185	-2,2723464	**0,02306559**	-5,568633	-0,4109641
2|3	-1,4994132	1,30528652	-1,1487234	0,25067005	-4,0577748	1,05894841
3|4	-0,3547413	1,3095762	-0,2708825	0,7864814	-2,9215106	2,21202807
**Burden related to coercion**						
Car/Street	-0,8068082	0,32268132	-2,500325	**0,01240794**	-1,4392635	-0,1743528
Escort/ Diverse	1,06657108	0,43614917	2,44542728	**0,01446806**	0,21171871	1,92142346
Client´s apartment/ Hotel	-0,6156925	0,33860528	-1,8183193	0,06901534	-1,2793589	0,04797382
Online	0,13541867	0,36560627	0,37039483	0,71108833	-0,5811696	0,85200696
Club	-0,8748884	0,47594547	-1,8382114	0,06603126	-1,8077415	0,05796474
Brothel	-0,461635	0,43923546	-1,0509967	0,29326011	-1,3225365	0,39926649
Studio	-0,1957978	0,3388701	-0,5777959	0,56340193	-0,8599831	0,46838764
Own apartment	-0,3419378	0,4429518	-0,7719526	0,44014248	-1,2101233	0,52624772
1|2	-1,7242874	1,85287782	-0,9305996	0,35206072	-5,3559279	1,90735315
2|3	-1,0172943	1,85315075	-0,5489539	0,58303709	-4,6494698	2,61488116
3|4	-0,3912136	1,8563871	-0,2107392	0,83309076	-4,0297323	3,24730513
**Burden related to violence**						
Car/Street	-0,8514328	0,24297352	-3,5042207	**0,00045795**	-1,3276609	-0,3752047
Escort/ Diverse	0,64638288	0,28531584	2,26549943	**0,02348205**	0,08716383	1,20560194
Client´s apartment/ Hotel	-0,6630425	0,25260572	-2,6248117	**0,00866969**	-1,1581497	-0,1679352
Online	0,01476125	0,26817221	0,0550439	0,95610349	-0,5108563	0,54037878
Club	-0,8276131	0,38814114	-2,1322477	**0,03298649**	-1,5883697	-0,0668564
Brothel	-0,573248	0,33655706	-1,7032714	0,08851729	-1,2328999	0,08640382
Studio	0,49864825	0,26339611	1,89314961	0,05833798	-0,0176081	1,01490462
Own apartment	-0,3802394	0,33737259	-1,1270608	0,25971676	-1,0414897	0,28101084
1|2	-2,9192652	1,49609644	-1,9512547	0,05102675	-5,8516143	0,01308378
2|3	-1,9238313	1,49174224	-1,289654	0,19717083	-4,8476461	0,9999835
3|4	-0,8796018	1,49274535	-0,5892511	0,55569287	-3,8053827	2,04617911
**Burden related to double life**						
Car/Street	0,22842516	0,22094563	1,03385237	0,30120519	-0,2046283	0,66147858
Escort/ Diverse	-0,1846339	0,22294627	-0,8281543	0,4075831	-0,6216086	0,25234077
Client´s apartment/ Hotel	-0,5554057	0,20675151	-2,6863439	**0,00722387**	-0,9606386	-0,1501727

Online	-0,0352834	0,20612042	-0,1711786	0,86408335	-0,4392794	0,36871262
Club	-0,1954942	0,33419282	-0,5849744	0,55856493	-0,8505122	0,45952368
Brothel	-0,4313501	0,28423322	-1,5175921	0,12911727	-0,9884472	0,12574703
Studio	0,08536656	0,20123632	0,42421048	0,67141232	-0,3090566	0,47978975
Own apartment	0,10880895	0,28510713	0,38164235	0,70272667	-0,450001	0,66761893
1|2	-2,2400649	1,23889851	-1,8081101	0,07058937	-4,668306	0,18817616
2|3	-0,8340403	1,23439017	-0,6756699	0,49925025	-3,253445	1,58536445
3|4	0,43274818	1,2363944	0,3500082	0,72633254	-1,9905848	2,85608119
**Burden related to relationship difficulties**						
Car/Street	0,01939806	0,22975403	0,08442968	0,93271481	-0,4309198	0,46971596
Escort/ Diverse	-0,5331992	0,22869121	-2,3315246	**0,01972572**	-0,9814339	-0,0849644
Client´s apartment/ Hotel	-0,6779138	0,22251836	-3,046552	**0,00231482**	-1,1140497	-0,2417778
Online	0,5126384	0,22797602	2,24865051	**0,02453474**	0,06580539	0,9594714
Club	-0,0110209	0,36449031	-0,0302365	0,97587845	-0,7254219	0,70338011
Brothel	-0,2463336	0,30391801	-0,8105265	0,41763764	-0,8420129	0,3493457
Studio	0,3945926	0,22135267	1,78264212	0,07464458	-0,0392586	0,82844384
Own apartment	-0,5838902	0,30045427	-1,9433581	0,0519729	-1,1727806	0,00500012
1|2	-1,7084649	1,3593838	-1,2567936	0,20882837	-4,3728572	0,95592732
2|3	-0,6999284	1,35759957	-0,5155632	0,60615952	-3,3608235	1,96096679
3|4	0,45058484	1,3603298	0,33123206	0,74046921	-2,2156616	3,11683124
**Burden related to financial dependence**						
Car/Street	-0,9177324	0,22389544	-4,0989329	**4,1506E-05**	-1,3565674	-0,4788973
Escort/ Diverse	0,27759539	0,23976664	1,15777322	0,2469566	-0,1923472	0,74753799
Client´s apartment/ Hotel	-0,8884037	0,21994704	-4,0391712	**5,364E-05**	-1,3194999	-0,4573075
Online	0,27189256	0,22718586	1,19678469	0,23139049	-0,1733917	0,71717684
Club	-0,125707	0,35536152	-0,3537441	0,72353071	-0,8222156	0,57080156
Brothel	-0,4759928	0,29741089	-1,6004552	0,10949765	-1,0589181	0,10693255
Studio	0,64147094	0,22640285	2,83331651	**0,00460677**	0,19772135	1,08522054
Own apartment	-0,3086176	0,30124973	-1,0244577	0,30561914	-0,8990671	0,28183186
1|2	-2,5057855	1,34095056	-1,8686636	0,06166963	-5,1340486	0,12247755
2|3	-1,3425104	1,33608929	-1,0048059	0,31499031	-3,9612455	1,27622456
3|4	-0,3487094	1,33673407	-0,2608667	0,79419528	-2,9687082	2,27128933
**Burden related to fear of arrest**						
Car/Street	-0,6190381	0,25142824	-2,4620866	**0,01381313**	-1,1118375	-0,1262387
Escort/ Diverse	0,15609628	0,27365649	0,57040959	0,56839992	-0,3802704	0,692463
Client´s apartment/ Hotel	-1,1991848	0,28180765	-4,255331	**2,0874E-05**	-1,7515278	-0,6468418
Online	0,27594002	0,27599595	0,99979736	0,31740858	-0,265012	0,81689207
Club	0,20036465	0,43270538	0,46305097	0,64332783	-0,6477379	1,04846719
Brothel	-0,4003177	0,35920215	-1,1144634	0,26508042	-1,1043539	0,30371855
Studio	0,28491914	0,26915839	1,05855566	0,28980219	-0,2426313	0,81246959
Own apartment	-0,0568543	0,34715069	-0,1637741	0,86990899	-0,7372696	0,62356106
1|2	-0,9250941	1,47774718	-0,6260165	0,53130411	-3,8214786	1,97129034
2|3	0,25257523	1,48008659	0,17064896	0,8644998	-2,6483945	3,15354495
3|4	1,25860511	1,49444203	0,84219065	0,39968124	-1,6705013	4,18771149
**Burden related to sexual difficulties**						
Car/Street	-0,7799549	0,23274259	-3,3511482	**0,00080477**	-1,2361304	-0,3237794
Escort/ Diverse	-0,0874574	0,24386015	-0,3586375	0,7198663	-0,5654233	0,39050851

Client´s apartment/ Hotel	-0,3103624	0,22861005	-1,357606	0,17458875	-0,7584381	0,13771333
Online	0,08330063	0,23681011	0,35176128	0,7250173	-0,3808472	0,54744845
Club	-0,0072266	0,37963794	-0,0190355	0,98481281	-0,7513169	0,73686377
Brothel	0,20145636	0,34774676	0,57931914	0,56237385	-0,4801273	0,88304002
Studio	0,43605313	0,2360219	1,84751129	0,06467307	-0,0265498	0,89865606
Own apartment	-0,0753265	0,32630118	-0,2308497	0,81743155	-0,7148769	0,56422378
1|2	-0,3460745	1,46761159	-0,235808	0,81358169	-3,2225932	2,53044423
2|3	0,87614828	1,47014414	0,5959608	0,55120142	-2,0053342	3,75763079
3|4	1,67903485	1,47336293	1,13959352	0,25445569	-1,2087565	4,5668262
**Burden related to perceived guilt or shame**						
Car/Street	-0,558599	0,23120977	-2,4159837	**0,01569276**	-1,0117702	-0,1054279
Escort/ Diverse	0,00275653	0,24618742	0,0111969	0,99106636	-0,4797708	0,48528387
Client´s apartment/ Hotel	-0,45706	0,22934798	-1,9928668	**0,04627604**	-0,906582	-0,0075379
Online	0,76366569	0,25123425	3,03965601	**0,00236848**	0,27124657	1,25608482
Club	-0,0831303	0,37080916	-0,2241863	0,82261235	-0,8099163	0,64365563
Brothel	0,34734136	0,33996827	1,02168757	0,3069288	-0,3189965	1,01367918
Studio	0,54569227	0,23868799	2,28621587	**0,02224163**	0,07786381	1,01352072
Own apartment	-0,3284125	0,31438542	-1,0446175	0,29619977	-0,944608	0,2877829
1|2	0,93083089	1,49269431	0,6235911	0,53289616	-1,99485	3,85651174
2|3	2,03389238	1,49665701	1,35895691	0,17416024	-0,8995554	4,96734011
3|4	2,82448022	1,50065448	1,88216558	0,05981354	-0,1168026	5,765763
**Burden related to health**						
Car/Street	-1,0305405	0,2308177	-4,4647376	**8,0167E-06**	-1,4829431	-0,5781378
Escort/ Diverse	0,41934539	0,26115008	1,60576395	**0,10832579**	-0,0925088	0,93119955
Client´s apartment/ Hotel	-0,7589167	0,23977513	-3,1651186	**0,0015502**	-1,228876	-0,2889575
Online	0,42563618	0,25207545	1,68852693	0,09131012	-0,0684317	0,91970406
Club	-0,5639714	0,36830707	-1,5312534	0,12570679	-1,2858533	0,15791042
Brothel	-0,4056203	0,33330397	-1,2169681	0,22361637	-1,0588961	0,2476555
Studio	0,47078919	0,24543767	1,91816194	0,05509048	-0,0102686	0,95184702
Own apartment	-0,1775348	0,32361518	-0,5485986	0,58328096	-0,8118206	0,45675093
1|2	-2,2138768	1,4182524	-1,5609893	0,11852628	-4,9936515	0,56589793
2|3	-0,9443734	1,41533832	-0,6672422	0,50461746	-3,7184365	1,8296897
3|4	0,17999889	1,42116262	0,12665608	0,89921261	-2,6054798	2,96547762

PPO Models

Work-related strain / burden:

**Predictor**	**Estimate**	**Std. Error**	**z value**	**p-value**	**OR**	**95% CI (β)**
(Intercept):1	2.2164	1.4025	1.580	0.1140	–	(-0.532, 4.965)
(Intercept):2	0.9469	1.3992	0.677	0.4986	–	(-1.795, 3.689)
(Intercept):3	-0.1775	1.4050	-0.126	0.8994	–	(-2.931, 2.576)
Car_Street	-1.0307	0.2315	-4.452	**<0.001**	0.36	(-1.484, -0.577)
Diverse_Escort	0.4193	0.2608	1.607	0.1080	1.52	(-0.092, 0.931)
Client_Hotel	-0.7590	0.2391	-3.174	**0.002**	0.47	(-1.227, -0.291)
online	0.4256	0.2531	1.681	0.0927	1.53	(-0.070, 0.921)
club	-0.5643	0.3645	-1.548	0.1216	0.57	(-1.278, 0.149)
brothel	-0.4059	0.3201	-1.268	0.2047	0.67	(-1.034, 0.222)

studio	0.4707	0.2468	1.907	0.0565	1.60	(-0.013, 0.955)
own_apartment	-0.1778	0.3239	-0.549	0.5831	0.84	(-0.813, 0.457)

Burden due to financial exploitation:

**Predictor**	**Estimate**	**Std. Error**	**z value**	**p-value**	**OR**	**95% CI (β)**
(Intercept)	2.7092	1.3920	1.946	**0.0516**	—	(-0.019, 5.438)
Diverse_Escort	0.7378	0.2690	2.743	**0.0061**	2.091	(0.211, 1.265)
Client_Hotel	-0.5750	0.2350	-2.447	**0.0144**	0.563	(-1.036, -0.114)
online	-0.2110	0.2404	-0.878	0.3800	0.810	(-0.682, 0.260)
club	-0.7077	0.3612	-1.959	0.0501	0.493	(-1.415, 0.000)
brothel	-0.4749	0.3204	-1.482	0.1383	0.622	(-1.103, 0.153)
studio	0.2299	0.2398	0.959	0.3377	1.259	(-0.240, 0.700)
Car_Street:1	-1.2612	0.2425	-5.201	**<0.001**	0.283	(-1.736, -0.787)
Car_Street:2	-1.1090	0.2629	-4.219	**<0.001**	0.330	(-1.624, -0.594)
Car_Street:3	-1.2570	0.3525	-3.566	**<0.001**	0.285	(-1.948, -0.566)
Own apartment:1	0.3804	0.3474	1.095	0.2736	1.463	(-0.301, 1.062)
Own apartment:2	-0.3836	0.3634	-1.056	0.2911	0.681	(-1.096, 0.329)
Own apartment:3	-0.8668	0.4078	-2.126	**0.0335**	0.420	(-1.666, -0.068)

Burden due to client demands:

**Predictor**	**Estimate**	**Std. Error**	**z value**	**p-value**	**95% CI (β)**	**OR**
(Intercept)	1.85581	1.31510	1.411	0.1582	(-0.722, 4.434)	—
Car_Street:1	0.01391	0.23512	0.059	0.9528	(-0.447, 0.475)	1.014
Car_Street:2	-1.00786	0.26065	-3.867	**0.00011**	(-1.519, -0.497)	0.365
Car_Street:3	-1.36921	0.36277	-3.774	**0.00016**	(-2.080, -0.658)	0.254
Diverse_Escort	0.12368	0.23219	0.533	0.5943	(-0.332, 0.579)	1.132
Client_Hotel	-0.49591	0.21572	-2.299	**0.02151**	(-0.918, -0.074)	0.609
online:1	-0.79065	0.22317	-3.543	**0.000396**	(-1.228, -0.353)	0.453
online:2	0.19496	0.22536	0.865	0.387	(-0.247, 0.637)	1.215
online:3	0.14648	0.38285	0.383	0.702	(-0.604, 0.897)	1.158
club	-0.03168	0.34879	-0.091	0.9276	(-0.715, 0.652)	0.969
brothel	-0.24430	0.30119	-0.811	0.4173	(-0.835, 0.347)	0.783
studio	0.05660	0.21298	0.266	0.7904	(-0.361, 0.474)	1.058
Own apartment:1	0.39254	0.31093	1.262	0.2068	(-0.217, 1.002)	1.481
Own apartment:2	-0.55692	0.32366	NA	NA	NA	0.573
Own	-0.85464	0.43700	-1.956	0.0505		0.425

apartment:3					(-1.711, 0.002)	

Desire to leave the industry:

```
workplace_vars <- c(
  "Car_Street", "Diverse_Escort", "Client_Hotel",
  "online", "club", "brothel", "studio", "own_apartment"
)
data <- data %>%
  mutate(across(all_of(workplace_vars), ~ relevel(as.factor(.), ref = "2")))
data$Chiffre <- as.factor(data$Chiffre)
data$Ausstiegswunsch <- factor(data$Ausstiegswunsch, levels = c("2", "1"))
form <- as.formula(
  paste("Ausstiegswunsch ~", paste(workplace_vars, collapse = " + "))
)
model <- glm(form, data = data, family = binomial(link = "logit"))
results <- broom::tidy(model, conf.int = TRUE) %>%
  mutate(
    OR = exp(estimate),
    conf.low.OR = exp(conf.low),
    conf.high.OR = exp(conf.high)
  )
results_eu <- results %>%
  mutate(across(c(estimate, std.error, statistic, p.value, OR, conf.low.OR, conf.high.OR),
                ~ format(., decimal.mark = ",", scientific = FALSE)))
```

term	estimate	std.error	statistic	p.value	conf.low	conf.high	OR	conf.low.OR	conf.high.OR
(Intercept)	-0,7265047	0,2637123	-2,7549139	0,00587076	-1,2433713	-0,2096381	0,48359637	0,28841027	0,81087768
Car/Street	1,30830981	0,30677922	4,26466241	2,002E-05	0,70703359	1,90958604	3,69991487	2,02796654	6,75029384
Diverse/Escort	-0,8317713	0,31229452	-2,6634195	0,00773509	-1,4438573	-0,2196853	0,43527759	0,23601561	0,80277139
Client/Hotel	0,64559061	0,27915624	2,31264979	0,0207419	0,09845444	1,19272678	1,90711306	1,10346413	3,29605659
online	-0,9781279	0,30016695	-3,2586129	0,00111958	-1,5664443	-0,3898115	0,37601437	0,20878624	0,67718452
club	-0,2343056	0,43205438	-0,5423057	0,58760792	-1,0811166	0,61250547	0,79112005	0,33921655	1,84504832
brothel	0,94121279	0,37358865	2,51938273	0,01175608	0,20899249	1,67343309	2,56308802	1,23243575	5,33043627
studio	-0,3775679	0,27177981	-1,3892419	0,16475919	-0,9102465	0,15511073	0,68552665	0,402425	1,16778726
Own apartment	-0,1095838	0,37492185	-0,2922843	0,7700693	-0,8444171	0,62524956	0,8962071	0,42980783	1,86871226

Rule Breaking:

```
library(tidyverse)
library(haven)
library(broom)
library(writexl)

workplace_vars <- c("Car_Street", "Diverse_Escort", "Client_Hotel",
          "online", "club", "brothel", "studio", "own_apartment")
data <- data %>%
  mutate(across(all_of(workplace_vars), ~ relevel(as.factor(.), ref = "2")))
data$Chiffre <- as.factor(data$Chiffre)
data$Kunden_Regeln_brechen <- factor(data$Kunden_Regeln_brechen, levels = c("2", "1"))
form <- as.formula(
  paste("Kunden_Regeln_brechen ~", paste(workplace_vars, collapse = " + "))
)
model <- glm(form, data = data, family = binomial(link = "logit"))
results <- broom::tidy(model, conf.int = TRUE, conf.level = 0.95) %>%
  filter(term != "(Intercept)") %>%
  mutate(
    OR = exp(estimate),
    CI_Lower = exp(conf.low),
    CI_Upper = exp(conf.high)
  ) %>%
  select(
    Predictor = term,
    Beta = estimate,
    P_Value = p.value,
    OR,
    CI_Lower,
    CI_Upper
  ) %>%
  mutate(
    Beta = round(Beta, 3),
    P_Value = round(P_Value, 4),
    OR = round(OR, 2),
    CI_Lower = round(CI_Lower, 2),
    CI_Upper = round(CI_Upper, 2)
  )
```

Predictor	Beta	P_Value	OR	CI_Lower	CI_Upper
Car/Street	0,622	0,0226	1,86	1,09	3,18
Diverse/Escort	-0,112	0,6793	0,89	0,53	1,52
Client/Hotel	0,942	0,0003	2,57	1,53	4,3
online	-0,567	0,0271	0,57	0,34	0,94
club	0,084	0,8306	1,09	0,51	2,34
brothel	0,82	0,0282	2,27	1,09	4,72
studio	-0,744	0,0037	0,48	0,29	0,79
Own apartment	-0,244	0,4831	0,78	0,4	1,55

Specialization:

```
library(tidyverse)
library(haven)
library(broom)
library(writexl)
```

```
spez_vars <- paste0("Spezialisierung0", 1:5)
antworten <- 1:6
for (antw in antworten) {
  dummy_name <- paste0("Spezialisierung_dummy_", antw)
  data[[dummy_name]] <- apply(data[spez_vars], 1, function(x) ifelse(any(x == antw, na.rm = TRUE), 1, 0))
}
dummy_vars <- paste0("Spezialisierung_dummy_", antworten)
data <- data %>% mutate(across(all_of(dummy_vars), ~ factor(.)))
workplace_vars <- c("Car_Street", "Diverse_Escort", "Client_Hotel", "online", "club", "brothel", "studio",
"own_apartment")
results <- list()
for (wp in workplace_vars) {
  cat("----------------------------------------------------\n")
  cat("Modell für Arbeitsort:", wp, "\n")
  form <- as.formula(
    paste(wp, "~", paste(dummy_vars, collapse = " + "))
  )
  model <- glm(form, data = data, family = binomial(link = "logit"))
  tidy_out <- broom::tidy(model, conf.int = TRUE, conf.level = 0.95) %>%
    filter(term != "(Intercept)") %>%
    mutate(
      OR = exp(estimate),
      CI_Lower = exp(conf.low),
      CI_Upper = exp(conf.high),
      Beta = round(estimate, 3),
      P_Value = round(p.value, 4),
      OR = round(OR, 2),
      CI_Lower = round(CI_Lower, 2),
      CI_Upper = round(CI_Upper, 2),
      Workplace = wp
    ) %>%
    select(Workplace, term, Beta, P_Value, OR, CI_Lower, CI_Upper)
  results[[wp]] <- tidy_out
}
results_df <- bind_rows(results)
```

Street/Car:

**Prädiktor**	**Schätzer (Estimate)**	**Std.-Fehler**	**z-Wert**	**p-Wert**	**Signifikanz**
(Intercept)	-0,2702	0,4020	-0,672	0,50155	
None	-0,2865	0,4238	-0,676	0,49908	
Dominatrix	-0,1241	0,4159	-0,298	0,76542	
Fetish	-0,2082	0,4380	-0,475	0,63454	
BDSM	-0,9895	0,4802	-2,061	0,03932	*
Tantra	-1,1353	0,7907	-1,436	0,15107	
Other	-1,3207	0,4696	-2,813	0,00491	**

Diverse/Escort:

**Prädiktor**	**Schätzer (Estimate)**	**Std.-Fehler**	**z-Wert**	**p-Wert**	**Signifikanz**
(Intercept)	-1,2085	0,4142	-2,918	0,00352	**
None	-0,0624	0,4174	-0,149	0,88118	

Dominatrix	-0,4084	0,3906	-1,045	0,29582	
Fetish	1,0501	0,4408	2,382	0,01722	*
BDSM	-0,3022	0,4355	-0,694	0,48782	
Tantra	-0,2198	0,5127	-0,429	0,66810	
Other	0,3358	0,3947	0,851	0,39489	

Client_Hotel

**Prädiktor**	**Schätzer (Estimate)**	**Std.-Fehler**	**z-Wert**	**p-Wert**	**Signifikanz**
(Intercept)	0,2125	0,3416	0,622	0,5339	
None	0,2010	0,3725	0,539	0,5895	
Dominatrix	-0,7803	0,3656	-2,134	0,0328	*
Fetish	0,2709	0,3831	0,707	0,4795	
BDSM	0,6256	0,4025	1,554	0,1202	
Tantra	0,5350	0,4827	1,108	0,2677	
Other	-0,2874	0,3601	-0,798	0,4248	

Online:

**Prädiktor**	**Schätzer (Estimate)**	**Std.-Fehler**	**z-Wert**	**p-Wert**	**Signifikanz**
(Intercept)	-1,0304	0,3763	-2,738	0,00618	**
None	0,0376	0,3957	0,095	0,92438	
Dominatrix	0,2026	0,3676	0,551	0,58154	
Fetish	0,3838	0,3959	0,969	0,33231	
BDSM	-0,0322	0,4102	-0,078	0,93750	
Tantra	-0,9099	0,5551	-1,639	0,10119	
Other	0,4103	0,3785	1,084	0,27833	

Club:

**Prädiktor**	**Schätzer (Estimate)**	**Std.-Fehler**	**z-Wert**	**p-Wert**	**Signifikanz**
(Intercept)	-8,6553	17,7643	-0,487	0,626	
None	-3,6706	19,6081	-0,187	0,852	
Dominatrix	-25,0996	25,9184	-0,968	0,333	
Fetish	-2,7501	26,1213	-0,105	0,916	
BDSM	-0,9200	20,9972	-0,044	0,965	
Tantra	0,3945	22,7594	0,017	0,986	
Other	-3,4674	17,7261	-0,196	0,845	

Brothel:

**Prädiktor**	**Schätzer (Estimate)**	**Std.-Fehler**	**z-Wert**	**p-Wert**	**Signifikanz**
None	13,9439	0,0020	6893,259	<2e-16	***

Dominatrix	-1,4184	0,0020	-701,227	<2e-16	***
Fetish	5,7703	0,0020	2852,493	<2e-16	***
BDSM	10,5607	0,0020	5220,616	<2e-16	***
Tantra	-7,4696	44,99198	-0,166	0,868	
Other	1,5538	0,0020	768,170	<2e-16	***

Studio:

**Prädiktor**	**Schätzer (Estimate)**	**Std.-Fehler**	**z-Wert**	**p-Wert**	**Signifikanz**
(Intercept)	-11,1848	1,8528	-6,037	1,57e-09	***
None	-0,6572	1,9697	-0,334	0,739	
Dominatrix	0,0370	1,7576	0,021	0,983	
Fetish	0,4126	1,8885	0,219	0,827	
BDSM	22,1673	2,4429	9,074	<2e-16	***
Tantra	-0,9206	2,6314	-0,350	0,726	
Other	-0,4541	1,8708	-0,243	0,808	

Own Apartment:

**Prädiktor**	**Schätzer (Estimate)**	**Std.-Fehler**	**z-Wert**	**p-Wert**	**Signifikanz**
(Intercept)	-32,3811	5,5418	-5,843	5,12e-09	***
None	19,7273	5,1886	3,802	0,000143	***
Dominatrix	0,7554	2,6671	0,283	0,777004	
Fetish	20,3261	5,1053	3,981	6,85e-05	***
BDSM	-0,7635	3,2404	-0,236	0,813724	
Tantra	1,3907	2,6865	0,518	0,604683	
Other	20,0098	4,5765	4,372	1,23e-05	***

Positive Aspects:

```
library(tidyverse)
library(broom)
library(writexl)
workplace_vars <- c("Car_Street", "Diverse_Escort", "Client_Hotel",
                    "online", "club", "brothel", "studio", "own_apartment")
positive_aspects <- paste0("pos_Asp_", sprintf("%02d", 1:10))
data <- data %>%
  mutate(across(all_of(workplace_vars), ~ relevel(as.factor(.), ref = "2")))
data <- data %>%
  mutate(across(all_of(positive_aspects), ~ relevel(as.factor(.), ref = "2")))
results <- list()
for (asp in positive_aspects) {
  cat("---------------------------------------------------\n")
  cat("Modell für positiven Aspekt:", asp, "\n")
  formula_glm <- as.formula(paste(asp, "~", paste(workplace_vars, collapse = " + ")))
  tryCatch({
    model <- glm(formula_glm, data = data, family = binomial(link = "logit"))
    tidy_out <- broom::tidy(model, conf.int = TRUE, conf.level = 0.95) %>%
      filter(term != "(Intercept)") %>%
      mutate(
```

```
      OR = exp(estimate),
      CI_Lower = exp(conf.low),
      CI_Upper = exp(conf.high),
      Beta = round(estimate, 3),
      P_Value = round(p.value, 4),
      OR = round(OR, 2),
      CI_Lower = round(CI_Lower, 2),
      CI_Upper = round(CI_Upper, 2),
      Positive_Aspect = asp
    ) %>%
    select(Positive_Aspect, term, Beta, P_Value, OR, CI_Lower, CI_Upper)
  results[[asp]] <- tidy_out
 }, error = function(e) {
  cat("Fehler bei", asp, ":", e$message, "\n")
 })
}
results_df <- bind_rows(results)
```

Ergebnisse für positiven Aspekt: power/dominance

```
               Estimate Std. Error z value Pr(>|z|)
(Intercept)     -1.65894    0.34920  -4.751 2.03e-06 ***
Car_Street1     -0.64305    0.32714  -1.966   0.0493 *
Diverse_Escort1  0.34511    0.29741   1.160   0.2459
Client_Hotel1    0.07979    0.28208   0.283   0.7773
online1          0.38657    0.27796   1.391   0.1643
club1            0.93790    0.42528   2.205   0.0274 *
brothel1        -1.28935    0.52376  -2.462   0.0138 *
studio1          1.51930    0.33366   4.554 5.28e-06 ***
own_apartment1   0.16556    0.38218   0.433   0.6649
---
Signif. codes:  0 ‘***’ 0.001 ‘**’ 0.01 ‘*’ 0.05 ‘.’ 0.1 ‘ ’ 1
```

Ergebnisse für positiven Aspekt: sexual satisfaction

```
               Estimate Std. Error z value Pr(>|z|)
(Intercept)      -1.5637     0.3352  -4.665 3.09e-06 ***
Car_Street1      -0.7884     0.3165  -2.491   0.0127 *
Diverse_Escort1   0.5632     0.2844   1.980   0.0477 *
Client_Hotel1     0.3020     0.2775   1.088   0.2765
online1           0.6966     0.2775   2.510   0.0121 *
club1            -0.1082     0.4323  -0.250   0.8023
brothel1         -0.4321     0.4273  -1.011   0.3119
studio1           0.3648     0.2614   1.396   0.1628
own_apartment1    0.2918     0.3575   0.816   0.4144
---
Signif. codes:  0 ‘***’ 0.001 ‘**’ 0.01 ‘*’ 0.05 ‘.’ 0.1 ‘ ’ 1
```

Ergebnisse für positiven Aspekt: doing something good or helping someone

```
               Estimate Std. Error z value Pr(>|z|)
(Intercept)     -0.90486    0.24211  -3.737 0.000186 ***
Car_Street1     -0.92102    0.27967  -3.293 0.000991 ***
Diverse_Escort1  0.71935    0.26875   2.677 0.007436 **
```

```
Client_Hotel1    0.43276    0.25202   1.717 0.085953 .
online1          0.68820    0.25222   2.729 0.006362 **
club1            0.48658    0.39282   1.239 0.215459
brothel1        -0.63606    0.36940  -1.722 0.085096 .
studio1          0.78124    0.24952   3.131 0.001742 **
own_apartment1  -0.03755    0.34451  -0.109 0.913199
---
Signif. codes:  0 '***' 0.001 '**' 0.01 '*' 0.05 '.' 0.1 ' ' 1

----------------------------------------------------
Ergebnisse für positiven Aspekt: Independence

               Estimate Std. Error z value Pr(>|z|)
(Intercept)      0.32760    0.24111   1.359 0.174238
Car_Street1     -1.23379    0.26397  -4.674 2.96e-06 ***
Diverse_Escort1  1.10497    0.31342   3.526 0.000423 ***
Client_Hotel1    0.03401    0.25912   0.131 0.895570
online1          1.09795    0.28973   3.790 0.000151 ***
club1            0.22926    0.43402   0.528 0.597341
brothel1        -0.73016    0.34098  -2.141 0.032245 *
studio1          0.50053    0.26645   1.879 0.060309 .
own_apartment1   0.25126    0.38223   0.657 0.510956
---
Signif. codes:  0 '***' 0.001 '**' 0.01 '*' 0.05 '.' 0.1 ' ' 1


----------------------------------------------------
Ergebnisse für positiven Aspekt: freedom of choice (working hours, atmosphere, etc.)

               Estimate Std. Error z value Pr(>|z|)
(Intercept)       0.1892     0.2410   0.785 0.432347
Car_Street1      -1.0753     0.2868  -3.749 0.000178 ***
Diverse_Escort1   1.1419     0.3302   3.458 0.000544 ***
Client_Hotel1     0.1867     0.2581   0.723 0.469470
online1           0.9043     0.2901   3.118 0.001822 **
club1             0.5031     0.4525   1.112 0.266260
brothel1         -0.4841     0.3506  -1.381 0.167341
studio1           0.4693     0.2701   1.737 0.082304 .
own_apartment1   -0.1944     0.3655  -0.532 0.594873
---
Signif. codes:  0 '***' 0.001 '**' 0.01 '*' 0.05 '.' 0.1 ' ' 1

----------------------------------------------------
Ergebnisse für positiven Aspekt: Stating self-confidence and/or self-respect

               Estimate Std. Error z value Pr(>|z|)
(Intercept)      -0.7340     0.2384  -3.079  0.00208 **
Car_Street1      -1.2517     0.2979  -4.201 2.65e-05 ***
Diverse_Escort1   0.7686     0.2755   2.790  0.00527 **
Client_Hotel1     0.2596     0.2526   1.028  0.30403
online1           0.8170     0.2592   3.152  0.00162 **
club1             0.6336     0.4008   1.581  0.11389
brothel1         -0.1894     0.3465  -0.547  0.58460
studio1           0.8131     0.2538   3.204  0.00135 **
own_apartment1   -0.1926     0.3527  -0.546  0.58500
---
```

```
Signif. codes:  0 ‘***’ 0.001 ‘**’ 0.01 ‘*’ 0.05 ‘.’ 0.1 ‘ ’ 1


-----------------------------------------------------
Ergebnisse für positiven Aspekt: recognition and support from friends

               Estimate Std. Error z value Pr(>|z|)
(Intercept)      -1.5437     0.2634  -5.860 4.63e-09 ***
Car_Street1      -0.6852     0.3438  -1.993   0.0463 *
Diverse_Escort1   0.6442     0.3016   2.136   0.0327 *
Client_Hotel1    -0.4013     0.2923  -1.373   0.1698
online1           0.6714     0.2748   2.443   0.0146 *
club1            -0.3536     0.4813  -0.735   0.4625
brothel1          0.2364     0.3981   0.594   0.5527
studio1           0.1866     0.2764   0.675   0.4997
own_apartment1    0.3597     0.3844   0.936   0.3494
---
Signif. codes:  0 ‘***’ 0.001 ‘**’ 0.01 ‘*’ 0.05 ‘.’ 0.1 ‘ ’ 1

-----------------------------------------------------
Ergebnisse für positiven Aspekt: Money

                Estimate Std. Error z value Pr(>|z|)
Car_Street1      -0.6269    0.4561   -1.37    0.1493
Diverse_Escort1   1.1692    0.3735    3.13    0.00095 ***
Client_Hotel1    -0.0671    0.3532   -0.18    0.8545
online1           0.5360    0.3441    1.56    0.1189
club1             0.2497    0.5074    0.49    0.6252
brothel1          0.2000    0.5039    0.40    0.6878
studio1           0.8674    0.3264    2.66    0.00771 **
own_apartment1    0.3811    0.4645    0.82    0.4129
---
Signif. codes:  0 ‘***’ 0.001 ‘**’ 0.01 ‘*’ 0.05 ‘.’ 0.1 ‘ ’ 1

-----------------------------------------------------
Ergebnisse für positiven Aspekt: Community / Network

               Estimate Std. Error z value Pr(>|z|)
(Intercept)     -1.22608    0.28033  -4.374 1.22e-05 ***
Car_Street1     -1.11315    0.33961  -3.278  0.00105 **
Diverse_Escort1  0.84457    0.29099   2.902  0.00370 **
Client_Hotel1   -0.07922    0.26851  -0.295  0.76796
online1          0.42011    0.26440   1.589  0.11208
club1            0.84045    0.40252   2.088  0.03680 *
brothel1         0.47244    0.35629   1.326  0.18484
studio1          0.42099    0.25689   1.639  0.10126
own_apartment1  -0.25289    0.39278  -0.644  0.51968
---
Signif. codes:  0 ‘***’ 0.001 ‘**’ 0.01 ‘*’ 0.05 ‘.’ 0.1 ‘ ’ 1

-----------------------------------------------------
Ergebnisse für positiven Aspekt: Other

               Estimate Std. Error z value Pr(>|z|)
(Intercept)       -7.263      5.931  -1.224   0.2208
Car_Street1       -3.321      7.843  -0.423   0.6720
```

```
Diverse_Escort1    0.351      6.535   0.054   0.9572
Client_Hotel1     -1.807      6.596  -0.274   0.7841
online1           -2.828      7.535  -0.375   0.7074
club1             -7.732      8.872  -0.872   0.3835
brothel1         -16.075      8.127  -1.978   0.0479 *
studio1           -4.918      6.385  -0.770   0.4412
own_apartment1    -9.201     10.214  -0.901   0.3677
---
Signif. codes:  0 '***' 0.001 '**' 0.01 '*' 0.05 '.' 0.1 ' ' 1
```

Positive_Aspect	Workplace_Variable	OR	Lower_CI	Upper_CI	P_Value
power/dominance	Car_Street1	0.526	0.277	0.999	0.0493
power/dominance	Diverse_Escort1	1.412	0.789	2.527	0.2459
power/dominance	Client_Hotel1	1.083	0.624	1.879	0.7773
power/dominance	online1	1.472	0.849	2.550	0.1643
power/dominance	club1	2.554	1.106	5.894	0.0274
power/dominance	brothel1	0.276	0.099	0.769	0.0138
power/dominance	studio1	4.571	2.377	8.791	5.28e-06
power/dominance	own_apartment1	1,20587445	0,0183044	79,44171	0,930182809
sexual satisfaction	Car_Street1	0,31765753	0,18207114	0,55421362	5,38284E-05
sexual satisfaction	Diverse_Escort1	1,38112993	0,78908677	2,41737661	0,258232619
sexual satisfaction	Client_Hotel1	1,25870826	0,7658712	2,06868531	0,364047203
sexual satisfaction	online1	2,19975693	1,27158612	3,80542889	0,004814921
sexual satisfaction	club1	1,26287876	0,54437813	2,92969664	0,586715613
sexual satisfaction	brothel1	1,143303	0,56920284	2,29644277	0,706657022
sexual satisfaction	studio1	0,63051861	0,38229224	1,03992098	0,070820138
sexual satisfaction	own_apartment1	0,79097034	0,39396912	1,58802823	0,509632011
doing something good or helping someone	Car_Street1	0,203058	0,10027836	0,41118097	9,47614E-06
doing something good or helping someone	Diverse_Escort1	3,92643864	1,85219291	8,32360406	0,000359999
doing something good or helping someone	Client_Hotel1	1,29269537	0,74759766	2,23524151	0,358173972
doing something good or helping someone	online1	2,9261428	1,55487021	5,50676937	0,000874158
doing something good or helping someone	club1	1,68304447	0,6465654	4,38105517	0,286168914
doing something good or helping someone	brothel1	0,71573458	0,34703878	1,47613471	0,365174956
doing something good or helping someone	studio1	2,17304351	1,183779	3,98902001	0,012267665
doing something good or helping someone	own_apartment1	1,22547877	0,55647595	2,69876568	0,61369778
Independence	Car_Street1	0,28561047	0,15803302	0,5161791	3,32453E-05
Independence	Diverse_Escort1	3,7367461	1,98476895	7,03521253	4,43709E-05
Independence	Client_Hotel1	1,2138091	0,72879145	2,02161062	0,456601606
Independence	online1	2,38152742	1,38168818	4,10488627	0,001784938
Independence	club1	1,74018087	0,74238301	4,07906623	0,202453533
Independence	brothel1	0,6792473	0,33840603	1,36338261	0,276592936
Independence	studio1	2,68930681	1,54898093	4,66911565	0,000440406
Independence	own_apartment1	1,03384743	0,50544562	2,11464985	0,927357185
freedom of choice (working hours, atmosphere, etc.)	Car_Street1	0,31773398	0,17473713	0,5777529	0,000171104

freedom of choice (working hours, atmosphere, etc.)	Diverse_Escort1	2,65929229	1,51635143	4,66371803	0,000643781
freedom of choice (working hours, atmosphere, etc.)	Client_Hotel1	1,13797288	0,68584723	1,88814975	0,616869309
freedom of choice (working hours, atmosphere, etc.)	online1	2,38716559	1,4205215	4,01159684	0,001018491
freedom of choice (working hours, atmosphere, etc.)	club1	1,88958447	0,85346876	4,18355029	0,116592151
freedom of choice (working hours, atmosphere, etc.)	brothel1	0,45521965	0,2126219	0,97461706	0,042748491
freedom of choice (working hours, atmosphere, etc.)	studio1	2,85333266	1,68993129	4,81765581	8,73621E-05
freedom of choice (working hours, atmosphere, etc.)	own_apartment1	1,01397092	0,5043424	2,03856948	0,968940166
Stating self-confidence and/or self-respect	Car_Street1	0,24520773	0,12219381	0,49206116	7,63622E-05
Stating self-confidence and/or self-respect	Diverse_Escort1	2,39319889	1,35368214	4,23097916	0,002685557
Stating self-confidence and/or self-respect	Client_Hotel1	1,03976299	0,61340199	1,76247729	0,884854555
Stating self-confidence and/or self-respect	online1	2,16405702	1,27519097	3,67250309	0,004225301
Stating self-confidence and/or self-respect	club1	1,8158141	0,82252624	4,00860262	0,139832874
Stating self-confidence and/or self-respect	brothel1	0,58421313	0,26542198	1,28589568	0,181785748
Stating self-confidence and/or self-respect	studio1	2,80918301	1,65166233	4,77791922	0,000138018
Stating self-confidence and/or self-respect	own_apartment1	0,83342866	0,39502405	1,75838237	0,632417607
recognition and support from friends	Car_Street1	0,33061975	0,15577342	0,70172061	0,003945519
recognition and support from friends	Diverse_Escort1	2,10825608	1,13441238	3,91810222	0,018333871
recognition and support from friends	Client_Hotel1	0,9138399	0,51646591	1,61695736	0,756971101
recognition and support from friends	online1	2,15303913	1,21005987	3,83086622	0,009093415
recognition and support from friends	club1	1,46995817	0,63167938	3,42068632	0,371343584
recognition and support from friends	brothel1	0,66784269	0,27925183	1,59717437	0,364167752
recognition and support from friends	studio1	2,61156933	1,45951036	4,6730017	0,001222414
recognition and support from friends	own_apartment1	1,17797	0,54133148	2,56333386	0,679688133
Money	Car_Street1	0,53486024	0,22846654	1,25215485	0,149349089
Money	Diverse_Escort1	3,21960995	1,60940118	6,44083549	0,00094963

Money	Client_Hotel1	0,93507487	0,45631723	1,91613412	0,854493374
Money	online1	1,70701469	0,87173112	3,34265816	0,118854385
Money	club1	1,28350154	0,47144408	3,49431944	0,625241254
Money	brothel1	1,22185659	0,45977649	3,24708538	0,687825876
Money	studio1	2,38327249	1,25812888	4,51463111	0,007711513
Money	own_apartment1	1,46495376	0,58738901	3,6536086	0,412859215
Community / Network	Car_Street1	0,69636598	0,00080291	603,95732	0,91650408
Community / Network	Diverse_Escort1	2,95191367	0,01154413	754,824858	0,701959722
Community / Network	Client_Hotel1	1,07940622	0,00306437	380,214235	0,979625854
Community / Network	online1	2,01261332	0,00657941	615,649381	0,810697754
Community / Network	club1	4,1748741	0,00875402	1991,03675	0,649713608
Community / Network	brothel1	1,8778058	0,0017438	2022,11522	0,859598023
Community / Network	studio1	2,73284247	0,01230824	606,782966	0,715332191
Community / Network	own_apartment1	1,37286524	0,00070737	2664,46488	0,93461498

Income Categories:

```
library(nnet)
library(tidyverse)
data$Income <- as.factor(data$Income)
data$Income <- relevel(data$Income, ref = "low")
workplaces <- c("Car_Street", "Diverse_Escort", "Client_Hotel", "online",
        "club", "brothel", "studio", "own_apartment")
income_model <- multinom(
  formula = as.formula(paste("Income ~", paste(workplaces, collapse = " + "))),
  data = data
)
summary(income_model)
coefs <- summary(income_model)$coefficients
ses <- summary(income_model)$standard.errors
z_vals <- coefs / ses
p_vals <- 2 * (1 - pnorm(abs(z_vals)))
ORs <- exp(coefs)
results_table <- map2_dfr(
  split(coefs, row(coefs)),
  split(ses, row(ses)),
  ~ tibble(
     Outcome = rownames(coefs)[.x],
     Predictor = colnames(coefs),
     Estimate = as.numeric(.x),
     StdError = as.numeric(.y),
     z = as.numeric(.x)/as.numeric(.y),
     p = 2*(1 - pnorm(abs(as.numeric(.x)/as.numeric(.y)))),
     OR = exp(as.numeric(.x))
   )
)
results_table
```

Workplace_Variable	OR	Lower_CI	Upper_CI	P_Value
1|2	0,01122093	0,00424255	0,02967774	1,4567E-19
2|3	0,14820943	0,08584966	0,25586631	7,242E-12
3|4	0,54982591	0,36048858	0,83860779	0,00548341
4|5	5,06370927	3,00904307	8,52136409	1,0067E-09
Car/Street	0,25348854	0,15513156	0,4142061	4,3025E-08

Diverse/Escort	3,50868789	2,0763618	5,92906818	2,7387E-06
Client/Hotel	0,63781977	0,41553719	0,97900757	0,03968532
online	1,69860951	1,09067702	2,6453975	0,01907934
club	0,593828	0,30217066	1,16699513	0,13054819
brothel	1,30777701	0,71853517	2,38023244	0,37984642
studio	1,32432151	0,86305112	2,03212465	0,19851411
Own apartment	1,48478314	0,80969355	2,72273501	0,2013809

PPO Model

**Predictor**	**Estimate**	**Std. Error**	**z value**	**p-value**	**95% CI (β)**
(Intercept)	-0.149	1.299	-0.114	0.909	(-2.695, 2.397)
**Car_Street**	-1.380	0.226	-6.101	1.06e-09	**(-1.823, -0.937)**
**Diverse_Escort 1**	-1.374	0.301	-4.571	4.86e-06	**(-1.965, -0.783)**
**Diverse_Escort 2**	0.009	0.234	0.039	0.969	(-0.449, 0.467)
**Diverse_Escort 3**	0.681	0.231	2.945	0.003	**(0.228, 1.134)**
**Diverse_Escort 4**	1.941	0.252	7.704	1.31e-14	**(1.447, 2.435)**
**Client_Hotel**	-0.472	0.214	-2.208	0.027	**(-0.891, -0.053)**
**online**	0.593	0.218	2.723	0.006	**(0.166, 1.020)**
**club**	-0.558	0.334	-1.670	0.095	(-1.213, 0.097)
**brothel**	0.258	0.307	0.842	0.400	(-0.344, 0.860)
**studio**	0.239	0.212	1.128	0.259	(-0.177, 0.655)
**own_apartment**	0.376	0.303	1.243	0.214	(-0.218, 0.970)
