# Supplementary material for: Quality of life, mental health, and socio-demographic differences across sex work settings: implications for specialized healthcare and support services
Source: Front Public Health. 2025 Dec 4;13:1703735. doi: 10.3389/fpubh.2025.1703735 (PMC12711543; doi:10.3389/fpubh.2025.1703735)
Supplement: Supplementary file 6 [file Supplementary_file_6.pdf]

Supplement 6
Quality of Life, Mental Health, and Socio-Demographic Differences Across Sex Work Settings: Implications for Specialized Healthcare and Support Services

R version 4.3.3 (2024-02-29) -- "Angel Food Cake"
Copyright (C) 2024 The R Foundation for Statistical Computing
Platform: x86_64-apple-darwin20 (64-bit)

Sociodemographic Variables as Predictors of Setting:

```
library(tidyverse)
library(haven)
data <- data %>%
  mutate(across(c(Car_Street, Diverse_Escort, Client_Hotel, online, club, brothel, studio, own_apartment),
              ~ relevel(as.factor(.), ref = "2")))
data$Chiffre <- as.factor(data$Chiffre)
categorical_cofactors <- c("deutsch", "Elternteil_Dtl", "Aufenthaltsstatus", "Kinder",
                         "Obdachlosigkeit", "Schulabschluss", "feste_Beziehung",
                         "soziale_Kontakte_außerhalb", "Person_involviert")
data <- data %>%
  mutate(across(all_of(categorical_cofactors), as.factor))
data$Alter_Beginn_Tätigkeit <- as.numeric(data$Alter_Beginn_Tätigkeit)
location_vars <- c("Car_Street", "Diverse_Escort", "Client_Hotel", "online",
                 "brothel", "studio", "own_apartment")
all_vars <- c(location_vars, categorical_cofactors, "Alter_Beginn_Tätigkeit", "Chiffre")
data <- data %>% drop_na(all_of(all_vars))
for (loc in location_vars) {
  cat("------------------------------------------------------\n")
  cat("Ergebnisse für Arbeitsort:", loc, "\n")
  tryCatch({
    form <- as.formula(paste(loc, "~", paste(c(categorical_cofactors, "Alter_Beginn_Tätigkeit"), collapse = " + 
")))
    model <- glm(form, data = data, family = binomial)
    print(summary(model))
  }, error = function(e) {
    cat("Fehler bei", loc, ":", e$message, "\n")
  })
  cat("\n")
}
```

Street/Car

term	OR	Lower_CI	Upper_CI	P_Value
(Intercept)	0,136171	0,044872	0,413232	0,000431
German Citizenship	0,863703	0,392666	1,899789	0,715615
Migration Background	3,177683	1,447547	6,975713	**0,003952**
Children	1,487233	0,755681	2,92698	0,25055
Homelessness	2,815381	1,431231	5,538151	**0,002712**
No qualification	2,635858	0,831867	8,351992	0,099532
elementary school	0,701171	0,274955	1,788078	0,457327
Secondary school leaving certificate	0,69171	0,205944	2,323262	0,550993
Completed apprenticeship	0,930933	0,221308	3,915974	0,922219
Fachabitur	0,550207	0,193344	1,565752	0,262846
Abitur	0,07319	0,016567	0,323345	**0,000562**
University degree	1,5E-14	0	Inf	0,999996
No stable Relationship	1,3475	0,719749	2,522764	0,351254
Social contacts beyond sex work	2,327191	1,068107	5,070485	**0,033521**
Involvement of third persons	0,61713	0,274707	1,386382	0,242468
Age at Beginning	0,748886	0,552822	1,014487	0,061888

Escort

term	OR	Lower_CI	Upper_CI	P_Value
(Intercept)	0,133857	0,031939	0,561	0,005949
German Citizenship	0,8689	0,434856	1,736179	0,690709
Migration Background	0,948563	0,522933	1,720625	0,862018
Children	0,987645	0,520815	1,872916	0,969628
Homelessness	0,583548	0,274069	1,242491	0,162448
No qualification	2,305241	0,481337	11,04037	0,296001
elementary school	2,702296	0,682303	10,70258	0,156894
Secondary school leaving certificate	4,524385	1,01102	20,24694	**0,048348**
Completed apprenticeship	4,064405	0,741362	22,2825	0,106258
Fachabitur	6,03217	1,477184	24,63272	**0,012299**
Abitur	3,945883	0,979285	15,89935	0,053542
University degree	8,698101	0,992791	76,20635	0,050769
No stable Relationship	0,830655	0,493927	1,396941	0,484196
Social contacts beyond sex work	0,61594	0,235348	1,612004	0,323523
Involvement of third persons	0,88415	0,463108	1,687987	0,709009
Age at Beginning	0,627174	0,445901	0,882139	**0,007351**

Hotel / Client´s apartment

term	OR	Lower_CI	Upper_CI	P_Value
(Intercept)	0,609805	0,23415	1,588138	0,311156
German Citizenship	0,60912	0,321088	1,155533	0,12915
Migration Background	1,390198	0,780194	2,477141	0,263656
Children	0,644978	0,371818	1,118818	0,118651
Homelessness	4,732633	2,300788	9,734849	2,4E-05
No qualification	0,760687	0,263205	2,198458	0,613451
elementary school	1,87577	0,742329	4,739828	0,183529
Secondary school leaving certificate	0,834831	0,286912	2,429115	0,740433
Completed apprenticeship	1,856533	0,498029	6,920714	0,356736
Fachabitur	1,674994	0,638933	4,391079	0,294188
Abitur	1,754031	0,679107	4,530396	0,245783
University degree	3,196697	0,451946	22,61083	0,244307
No stable Relationship	1,382147	0,857284	2,228352	0,184155
Social contacts beyond sex work	1,024276	0,494641	2,121013	0,948506
Involvement of third persons	1,165348	0,64697	2,099069	0,610301
Age at Beginning	0,861806	0,673667	1,102488	0,236602

Online

term	OR	Lower_CI	Upper_CI	P_Value
(Intercept)	0,111956	0,013661	0,91752	0,041334
German Citizenship	0,508534	0,253453	1,020334	0,057001
Migration Background	0,849999	0,477394	1,513421	0,580842
Children	0,921328	0,498192	1,703851	0,793933
Homelessness	0,41384	0,194413	0,880925	**0,022087**
No qualification	8,104152	0,892611	73,5788	0,063023

elementary school	14,63549	1,818023	117,819	**0,01168**
Secondary school leaving certificate	14,74029	1,703235	127,5668	**0,014541**
Completed apprenticeship	17,21932	1,778033	166,76	**0,01402**
Fachabitur	7,572472	0,914967	62,67148	0,060443
Abitur	6,634436	0,804459	54,71474	0,078775
University degree	3,28E-08	0	Inf	0,998237
No stable Relationship	0,713768	0,429702	1,185622	0,192798
Social contacts beyond sex work	0,579139	0,223029	1,503848	0,261907
Involvement of third persons	0,999789	0,534781	1,869134	0,999472
Age at Beginning	0,871187	0,654297	1,159973	0,345146

Brothel

**term**	**OR**	**Lower_CI**	**Upper_CI**	**P_Value**
(Intercept)	0,010489	0,001959	0,056171	1,02E-07
German Citizenship	1,341291	0,561575	3,203603	0,508601
Migration Background	3,416412	1,329419	8,779675	**0,010734**
Children	3,149995	1,397476	7,100279	**0,005657**
Homelessness	0,688684	0,272674	1,739386	0,430112
No qualification	1,675825	0,290287	9,674547	0,563805
elementary school	3,074284	0,717509	13,17226	0,130331
Secondary school leaving certificate	2,458172	0,397495	15,20171	0,33328
Completed apprenticeship	10,67999	1,848687	61,69903	**0,00813**
Fachabitur	7,846433	1,720533	35,7834	**0,007794**
Abitur	1,58904	0,303379	8,323067	0,583574
University degree	5,866984	0,397589	86,5757	0,197622
No stable Relationship	0,988666	0,47958	2,038156	0,975363
Social contacts beyond sex work	0,951041	0,331709	2,726725	0,925579
Involvement of third persons	1,138755	0,470263	2,75753	0,773379
Age at Beginning	0,891724	0,616014	1,290835	0,543696

Studio

**term**	**OR**	**Lower_CI**	**Upper_CI**	**P_Value**
(Intercept)	0,828012	0,287015	2,388739	0,726993
German Citizenship	1,762749	0,856922	3,626101	0,123467
Migration Background	0,503671	0,263115	0,964158	**0,038439**
Children	1,227684	0,667616	2,2576	0,509261
Homelessness	0,491816	0,232582	1,039991	**0,063263**
No qualification	0,05642	0,006119	0,520214	**0,011195**
elementary school	0,688434	0,24864	1,906132	0,472452
Secondary school leaving certificate	0,744833	0,227639	2,437088	0,626194
Completed apprenticeship	2,023246	0,519102	7,885782	0,309956
Fachabitur	0,579161	0,19871	1,688023	0,316972
Abitur	1,218492	0,43505	3,412765	0,706866
University degree	1,420836	0,203094	9,9401	0,723423
No stable Relationship	0,637769	0,375669	1,082734	0,095793
Social contacts beyond sex work	0,570947	0,235664	1,383243	0,214467
Involvement of third persons	1,844537	0,995806	3,416648	**0,051583**

Age at Beginning	1,006759	0,765171	1,324624	0,961625

Own apartment

term	OR	Lower_CI	Upper_CI	P_Value
(Intercept)	1,7E-06	7,28E-30	3,99E+17	0,628545
German Citizenship	0,126475	1,28E-15	1,25E+13	0,899908
Migration Background	5,182036	1,44E-11	1,86E+12	0,903536
Children	0,196181	1,86E-16	2,07E+14	0,926476
Homelessness	2,523933	1,78E-14	3,59E+14	0,955595
No qualification	3,304784	6,56E-25	1,66E+25	0,967144
elementary school	1,742719	3,64E-25	8,35E+24	0,984716
Secondary school leaving certificate	2,028888	5,86E-30	7,02E+29	0,983735
Completed apprenticeship	0,086031	3,65E-34	2,03E+31	0,948572
Fachabitur	0,401034	2,78E-26	5,79E+24	0,975339
Abitur	2,954274	1,81E-23	4,81E+23	0,968313
University degree	0	0	Inf	0,999924
No stable Relationship	2,674183	3,59E-10	1,99E+10	0,932409
Social contacts beyond sex work	0,575557	1,96E-20	1,69E+19	0,980731
Involvement of third persons	2,457675	2,14E-12	2,83E+12	0,949399
Age at Beginning	0,60584	3,7E-06	99324,79	0,934805
