# Supplementary material for: Quality of life, mental health, and socio-demographic differences across sex work settings: implications for specialized healthcare and support services
Source: Front Public Health. 2025 Dec 4;13:1703735. doi: 10.3389/fpubh.2025.1703735 (PMC12711543; doi:10.3389/fpubh.2025.1703735)
Supplement: Supplementary file 7 [file Supplementary_file_7.pdf]

R version 4.3.3 (2024-02-29) -- "Angel Food Cake"
Copyright (C) 2024 The R Foundation for Statistical Computing
Platform: x86_64-apple-darwin20 (64-bit)

Mental Diseases as Predictors of Setting

```
library(tidyverse)
library(openxlsx)

disorders <- c("Angststoerung", "Affektive", "Zwangsstoerung", "Essstoerung",
               "Somatisierung", "Sucht", "Schlafstoerung", "PTBS")

results_list <- map(location_vars, function(location_var) {
  form <- as.formula(paste0(
    location_var, " ~ ", paste(disorders, collapse = " + ")
  ))

  mod <- glm(form, data = data, family = binomial)

  coefs <- summary(mod)$coefficients
  ORs <- exp(coefs[, "Estimate"])
  CI <- exp(confint.default(mod))
  pvals <- coefs[, "Pr(>|z|)"]
  zvals <- coefs[, "z value"]
  estimates <- coefs[, "Estimate"]
  std_err <- coefs[, "Std. Error"]

  tibble(
    Outcome = location_var,
    Variable = rownames(coefs),
    Estimate = estimates,
    Std_Error = std_err,
    z_value = zvals,
    p_value = pvals,
    OR = ORs,
    CI_lower = CI[, 1],
    CI_upper = CI[, 2]
  )
})

results_df <- bind_rows(results_list)

results_df <- results_df %>%
  mutate(across(c(Estimate, Std_Error, z_value, p_value, OR, CI_lower, CI_upper),
                ~ format(., decimal.mark = ",", scientific = FALSE)))
```

Outcome	Predictor	Estimate	Odds_Ratio	CI_Lower	CI_Upper	p_value
Car_Street	(Intercept)	-1,2634068	0,28268932	0,1788247	0,44688039	6,40E-08
Car_Street	Anxiety Disorder	-0,0207577	0,97945627	0,57600801	1,6654883	0,93891297
Car_Street	Affective Disorder	0,02694991	1,02731634	0,56973233	1,85241177	0,92860751
Car_Street	OCD	-0,411578	0,66260381	0,27558487	1,59313467	0,35782356
Car_Street	Eating disorder	-0,3490544	0,70535479	0,27468986	1,81122591	0,46818099
Car_Street	Somatization	0,36571004	1,4415372	0,52435765	3,96300022	0,47846381
Car_Street	Addiction	1,41187289	4,10363388	2,16016329	7,79561949	1,61E-05
Car_Street	Sleep disorder	0,05931117	1,06110537	0,53662124	2,09821104	0,86461083
Car_Street	PTBS	0,10959466	1,11582569	0,57118413	2,17979964	0,74838277
Diverse_Escort	(Intercept)	-1,2200215	0,29522383	0,18451929	0,47234688	3,62E-07

Diverse_Escort	Anxiety Disorder	0,08318608	1,08674401	0,6388606	1,84862323	0,75892017
Diverse_Escort	Affective Disorder	-0,3881616	0,67830275	0,36539598	1,25916716	0,2187638
Diverse_Escort	OCD	0,24507781	1,27772073	0,58220497	2,80411598	0,54112273
Diverse_Escort	Eating disorder	0,86660422	2,37881918	1,02557628	5,51765951	0,04350843
Diverse_Escort	Somatization	0,29712923	1,34598923	0,48829882	3,7102015	0,56573337
Diverse_Escort	Addiction	0,156522	1,16943649	0,66118743	2,06837222	0,59059109
Diverse_Escort	Sleep disorder	-0,134214	0,87440293	0,43542846	1,75592679	0,70595392
Diverse_Escort	PTBS	0,39369658	1,48245068	0,76589492	2,86940147	0,2426383
Client_Hotel	(Intercept)	-0,2352839	0,79034644	0,59702594	1,04626526	0,10018492
Client_Hotel	Anxiety Disorder	0,28519808	1,33002545	0,80738522	2,1909835	0,26277591
Client_Hotel	Affective Disorder	0,48029976	1,6165589	0,91094056	2,86875215	0,10075047
Client_Hotel	OCD	0,08023979	1,08354686	0,49136544	2,38941059	0,84236511
Client_Hotel	Eating disorder	0,32311143	1,38141927	0,57203171	3,33603747	0,47258523
Client_Hotel	Somatization	0,02519842	1,02551858	0,3674478	2,86214359	0,96162142
Client_Hotel	Addiction	1,4889726	4,43253917	2,28046476	8,61552605	1,13E-05
Client_Hotel	Sleep disorder	-0,2740873	0,76026571	0,39572073	1,460636	0,41066847
Client_Hotel	PTBS	0,27360029	1,3146892	0,68474725	2,52415426	0,41103208
online	(Intercept)	-1,1434292	0,31872419	0,214666	0,47322403	1,43E-08
online	Anxiety Disorder	0,94912209	2,58344063	1,50442749	4,43634908	0,0005809
online	Affective Disorder	0,41026291	1,507214	0,85690009	2,65106058	0,15446072
online	OCD	-0,9418415	0,38990914	0,16106053	0,9439255	0,03680755
online	Eating disorder	0,13166274	1,14072353	0,4797476	2,71236413	0,76575677
online	Somatization	-0,3142608	0,73032851	0,26249775	2,03194019	0,5472117
online	Addiction	-0,4963254	0,60876353	0,33829601	1,09546973	0,09776816
online	Sleep disorder	0,2899475	1,33635733	0,7060979	2,52918317	0,37303497
online	PTBS	-0,1608249	0,85144116	0,438748	1,65231987	0,6344822
club	(Intercept)	-13,197185	1,86E-06	1,57E-17	219348,775	0,3103308
club	Anxiety Disorder	0,16748403	1,18232641	4,87E-18	2,87E+17	0,99345738
club	Affective Disorder	-0,134881	0,8738199	4,43E-20	1,72E+19	0,99525235
club	OCD	1,21520029	3,37096917	3,46E-18	3,29E+18	0,95414585
club	Eating disorder	0,258941	1,29555736	6,53E-22	2,57E+21	0,99174279
club	Somatization	-0,4023394	0,66875375	6,63E-32	6,75E+30	0,99118672
club	Addiction	0,3277782	1,38788111	7,17E-18	2,69E+17	0,98712292
club	Sleep disorder	-0,1742887	0,8400543	7,88E-24	8,96E+22	0,99485972
club	PTBS	0,42123132	1,52383674	2,43E-21	9,54E+20	0,98624431
brothel	(Intercept)	-12,22582	4,90E-06	2,75E-07	8,72E-05	8,57E-17
brothel	Anxiety Disorder	-0,4407281	0,64356765	0,01003533	41,2721336	0,83554034
brothel	Affective Disorder	-0,1601931	0,85197922	0,00826347	87,8406351	0,94600132
brothel	OCD	-0,0699099	0,9324778	0,00172305	504,636712	0,98263066
brothel	Eating disorder	0,25591477	1,29164263	0,00276867	602,578427	0,93494837
brothel	Somatization	0,09494834	1,09960205	0,00025196	4798,91742	0,9822853
brothel	Addiction	-1,1363401	0,32099167	0,00085968	119,852958	0,70688101
brothel	Sleep disorder	-0,3869009	0,67915839	0,00196277	235,003073	0,89680096
brothel	PTBS	0,55667865	1,74486754	0,01744131	174,560396	0,81273347
studio	(Intercept)	-0,6920485	0,50054966	0,35846723	0,69894802	4,85E-05
studio	Anxiety Disorder	-0,6723175	0,51052404	0,29178695	0,89323665	0,01849611
studio	Affective Disorder	0,42929745	1,5361779	0,8547583	2,76083022	0,15120848
studio	OCD	0,14054404	1,15089976	0,51423737	2,57579541	0,73240587
studio	Eating disorder	0,15178498	1,16390995	0,49163361	2,75547955	0,72994621
studio	Somatization	0,1546971	1,16730433	0,40826006	3,33757701	0,77287833
studio	Addiction	-0,6892924	0,50193111	0,2665967	0,9450036	0,03274571
studio	Sleep disorder	-0,1215315	0,88556313	0,44253134	1,77212774	0,73132297

studio	PTBS	0,1309457	1,13990589	0,58611195	2,21695775	0,6996242
own_apartment	(Intercept)	-13,053739	2,14E-06	7,56E-08	6,07E-05	2,01E-14
own_apartment	Anxiety Disorder	-0,0538726	0,94755277	0,02464429	36,4326227	0,97691755
own_apartment	Affective Disorder	0,2494645	1,283338	0,02977719	55,309326	0,89663172
own_apartment	OCD	0,37811535	1,4595313	0,0151459	140,647444	0,87112423
own_apartment	Eating disorder	0,9530075	2,59349787	0,02692555	249,808509	0,68259167
own_apartment	Somatization	0,21146081	1,23548155	0,00251933	605,880184	0,94666192
own_apartment	Addiction	0,34992364	1,41895919	0,03350197	60,0993068	0,85473391
own_apartment	Sleep disorder	0,54095861	1,71765262	0,0296631	99,4613174	0,79391963
own_apartment	PTBS	0,56740718	1,76368819	0,03642505	85,3971579	0,77439559
