# Supplementary material for: Quality of life, mental health, and socio-demographic differences across sex work settings: implications for specialized healthcare and support services
Source: Front Public Health. 2025 Dec 4;13:1703735. doi: 10.3389/fpubh.2025.1703735 (PMC12711543; doi:10.3389/fpubh.2025.1703735)
Supplement: Supplementary file 8 [file Supplementary_file_8.pdf]

R version 4.3.3 (2024-02-29) -- "Angel Food Cake"
Copyright (C) 2024 The R Foundation for Statistical Computing
Platform: x86_64-apple-darwin20 (64-bit)

Reasons as Predictors of Workplace:

```
library(tidyverse)

workplace_vars <- c("Car_Street", "Diverse_Escort", "Client_Hotel",
                    "online", "club", "brothel", "studio", "own_apartment")

reason_dummies <- grep("^Grund_", names(data), value = TRUE)

model_results_workplace <- map(workplace_vars, function(wp) {
  form <- as.formula(
    paste(wp, "~", paste(reason_dummies, collapse = " + "))
  )

  glm(form, data = data, family = binomial)
})
model_summaries_workplace <- lapply(model_results_workplace, summary)
model_summaries_workplace
```

(Model Output can be seen in Table 5)
