# Supplementary material for: Quality of life, mental health, and socio-demographic differences across sex work settings: implications for specialized healthcare and support services
Source: Front Public Health. 2025 Dec 4;13:1703735. doi: 10.3389/fpubh.2025.1703735 (PMC12711543; doi:10.3389/fpubh.2025.1703735)
Supplement: Supplementary file 9 [file Supplementary_file_9.pdf]

R version 4.3.3 (2024-02-29) -- "Angel Food Cake"
Copyright (C) 2024 The R Foundation for Statistical Computing
Platform: x86_64-apple-darwin20 (64-bit)

Code:

```
library(tidyverse)
library(haven)

bedarf_vars <- paste0("Bedarf", sprintf("%02d", 1:9))

for (i in 1:12) {
  dummy_var <- paste0("Bedarf_option_", sprintf("%02d", i))
  data <- data %>%
    mutate(
      !!dummy_var := apply(select(., all_of(bedarf_vars)), 1, function(row) {
        if (i %in% row) 1 else 2
      }) %>% factor(levels = c(2, 1), labels = c("nein", "ja"))
    )
}

workplace_vars <- c("Car_Street", "Diverse_Escort", "Client_Hotel",
                    "online", "club", "brothel", "studio", "own_apartment")

data <- data %>% mutate(across(all_of(workplace_vars), ~ relevel(as.factor(.), ref = "2")))
bedarf_dummy_vars <- paste0("Bedarf_option_", sprintf("%02d", 1:12))
data <- data %>% mutate(across(all_of(bedarf_dummy_vars), ~ relevel(as.factor(.), ref = "nein")))

model_results_glm <- map(bedarf_dummy_vars, function(bed) {
  cat("------------------------------------------------------\n")
  cat("Modell für Bedarf:", bed, "\n")

  formula_glm <- as.formula(paste(bed, "~", paste(workplace_vars, collapse = " + ")))

  tryCatch({
    model <- glm(formula_glm, data = data, family = binomial(link = "logit"))
    print(summary(model))
    return(model)
  }, error = function(e) {
    cat("Fehler bei", bed, ":", e$message, "\n")
    return(NULL)
  })
  cat("\n")
    •   })
```

Workplace	Bedarf_item	Estimate	Std_Error	z_value	p_value	OR_effect
Car_Street	A home/ safe place	2,31	0,297	7,77	7,77E-15	10,07
Car_Street	Protection from physical attacks	1,28	0,5	2,56	0,0105	3,6
Car_Street	Assistance in exiting the business	1,788	0,497	3,601	0,000317	5,98
Car_Street	Therapy to quit drugs and alcohol	1,524	0,525	2,905	0,00367	4,59
Car_Street	Medical support	1,315	0,485	2,714	0,00664	3,73
Car_Street	Attorney/ legal support	1,294	0,498	2,596	0,00942	3,65

Car_Street	Residence permit/ legal status	0,334	0,56	0,596	0,551	1,4
Car_Street	Better and safer working conditions	1,85	0,48	3,853	0,000117	6,36
Car_Street	Recognition of sex work as a normal occupation/work activity	-1,61	0,481	-3,344	0,000826	0,2
Car_Street	Another job/(another) education	1,46	0,485	3,013	0,00259	4,31
Car_Street	Professional interpreters	0,91	0,574	1,586	0,113	2,49
Car_Street	I don't need anything	-0,505	0,569	-0,888	0,375	0,6
Diverse_Escort	A home/ safe place	-1,112	0,346	-3,214	0,00131	0,33
Diverse_Escort	Protection from physical attacks	-0,719	0,598	-1,203	0,229	0,49
Diverse_Escort	Assistance in exiting the business	-0,405	0,578	-0,701	0,483	0,67
Diverse_Escort	Therapy to quit drugs and alcohol	0,046	0,601	0,077	0,939	1,05
Diverse_Escort	Medical support	0,045	0,555	0,081	0,935	1,05
Diverse_Escort	Attorney/ legal support	-0,057	0,574	-0,1	0,921	0,94
Diverse_Escort	Residence permit/ legal status	-0,936	0,702	-1,333	0,183	0,39
Diverse_Escort	Better and safer working conditions	0,096	0,545	0,177	0,859	1,1
Diverse_Escort	Recognition of sex work as a normal occupation/work activity	1,254	0,556	2,255	0,0241	3,5
Diverse_Escort	Another job/(another) education	-0,217	0,558	-0,388	0,698	0,81
Diverse_Escort	Professional interpreters	-1,29	0,796	-1,62	0,105	0,28
Diverse_Escort	I don't need anything	-0,607	0,641	-0,947	0,343	0,54
Client_Hotel	A home/ safe place	1,034	0,28	3,7	0,000216	2,81
Client_Hotel	Protection from physical attacks	1,356	0,504	2,689	0,00716	3,88
Client_Hotel	Assistance in exiting the business	1,152	0,489	2,357	0,0184	3,16
Client_Hotel	Therapy to quit drugs and alcohol	1,821	0,581	3,135	0,00172	6,18
Client_Hotel	Medical support	1,631	0,482	3,385	0,000713	5,11
Client_Hotel	Attorney/ legal support	1,413	0,503	2,806	0,00501	4,11
Client_Hotel	Residence permit/ legal status	0,469	0,542	0,864	0,388	1,6
Client_Hotel	Better and safer working conditions	1,183	0,457	2,586	0,0097	3,26

Client_Hotel	Recognition of sex work as a normal occupation/work activity	0,416	0,445	0,935	0,35	1,52
Client_Hotel	Another job/(another) education	0,706	0,464	1,522	0,128	2,03
Client_Hotel	Professional interpreters	0,769	0,585	1,314	0,189	2,16
Client_Hotel	I don’t need anything	-1,741	0,533	-3,268	0,00108	0,18
online	A home/ safe place	-0,913	0,315	-2,898	0,00375	0,4
online	Protection from physical attacks	-1,306	0,576	-2,266	0,0235	0,27
online	Assistance in exiting the business	-0,956	0,549	-1,742	0,0815	0,38
online	Therapy to quit drugs and alcohol	-1,027	0,605	-1,698	0,0896	0,36
online	Medical support	-0,49	0,518	-0,946	0,344	0,61
online	Attorney/ legal support	0,104	0,526	0,198	0,843	1,11
online	Residence permit/ legal status	-2,313	0,866	-2,671	0,00756	0,1
online	Better and safer working conditions	-0,677	0,508	-1,333	0,183	0,51
online	Recognition of sex work as a normal occupation/work activity	0,98	0,506	1,937	0,0527	2,67
online	Another job/(another) education	-0,575	0,519	-1,107	0,268	0,56
online	Professional interpreters	-1,186	0,713	-1,663	0,0962	0,31
online	I don’t need anything	0,292	0,557	0,524	0,6	1,34
club	A home/ safe place	-1,097	0,573	-1,916	0,0554	0,33
club	Protection from physical attacks	0,261	0,922	0,283	0,777	1,3
club	Assistance in exiting the business	-0,138	0,934	-0,147	0,883	0,87
club	Therapy to quit drugs and alcohol	0,555	0,939	0,591	0,554	1,74
club	Medical support	0,309	0,898	0,344	0,731	1,36
club	Attorney/ legal support	0,216	0,922	0,235	0,815	1,24
club	Residence permit/ legal status	-0,531	1,106	-0,48	0,631	0,59
club	Better and safer working conditions	0,544	0,886	0,614	0,539	1,72
club	Recognition of sex work as a normal occupation/work activity	0,943	0,913	1,033	0,302	2,57

club	Another job/(another) education	0,113	0,903	0,125	0,9	1,12
club	Professional interpreters	-0,255	1,109	-0,23	0,818	0,78
club	I don't need anything	-0,826	1,103	-0,749	0,454	0,44
brothel	A home/ safe place	-0,086	0,424	-0,202	0,84	0,92
brothel	Protection from physical attacks	0,314	0,715	0,439	0,66	1,37
brothel	Assistance in exiting the business	-0,934	0,783	-1,193	0,233	0,39
brothel	Therapy to quit drugs and alcohol	-1,429	0,963	-1,483	0,138	0,24
brothel	Medical support	0,045	0,699	0,065	0,948	1,05
brothel	Attorney/ legal support	0,082	0,722	0,114	0,909	1,09
brothel	Residence permit/ legal status	0,197	0,795	0,248	0,804	1,22
brothel	Better and safer working conditions	0,409	0,679	0,602	0,547	1,5
brothel	Recognition of sex work as a normal occupation/work activity	-0,931	0,676	-1,379	0,168	0,39
brothel	Another job/(another) education	-0,116	0,704	-0,165	0,869	0,89
brothel	Professional interpreters	0,169	0,831	0,203	0,839	1,18
brothel	I don't need anything	1,904	0,704	2,706	0,00681	6,71
studio	A home/ safe place	-0,553	0,31	-1,782	0,0748	0,58
studio	Protection from physical attacks	-0,333	0,534	-0,623	0,533	0,72
studio	Assistance in exiting the business	-1,664	0,583	-2,853	0,00434	0,19
studio	Therapy to quit drugs and alcohol	-1,073	0,604	-1,777	0,0755	0,34
studio	Medical support	-0,466	0,514	-0,906	0,365	0,63
studio	Attorney/ legal support	-0,282	0,53	-0,531	0,596	0,75
studio	Residence permit/ legal status	0,571	0,57	1,002	0,316	1,77
studio	Better and safer working conditions	-0,79	0,506	-1,559	0,119	0,45
studio	Recognition of sex work as a normal occupation/work activity	0,989	0,501	1,974	0,0484	2,69
studio	Another job/(another) education	-0,736	0,52	-1,417	0,157	0,48
studio	Professional interpreters	-0,243	0,622	-0,391	0,696	0,78

studio	I don't need anything	-0,756	0,594	-1,272	0,204	0,47
own_apartment	A home/ safe place	0,496	0,402	1,233	0,218	1,64
own_apartment	Protection from physical attacks	0,166	0,694	0,239	0,811	1,18
own_apartment	Assistance in exiting the business	0,52	0,673	0,773	0,44	1,68
own_apartment	Therapy to quit drugs and alcohol	0,079	0,739	0,107	0,915	1,08
own_apartment	Medical support	0,653	0,656	0,994	0,32	1,92
own_apartment	Attorney/ legal support	-0,073	0,704	-0,104	0,917	0,93
own_apartment	Residence permit/ legal status	-0,061	0,8	-0,076	0,939	0,94
own_apartment	Better and safer working conditions	0,678	0,648	1,047	0,295	1,97
own_apartment	Recognition of sex work as a normal occupation/work activity	-0,058	0,648	-0,089	0,929	0,94
own_apartment	Another job/(another) education	0,073	0,67	0,109	0,913	1,08
own_apartment	Professional interpreters	-0,659	0,948	-0,695	0,487	0,52
own_apartment	I don't need anything	-1,234	0,941	-1,312	0,19	0,29
